# SMIntegration: A web tool for comprehensive spatial metabolomics and transcriptomics integrated analysis and visualization

**DOI:** 10.1093/gigascience/giag033

**Published:** 2026-03-24

**Authors:** Haoke Deng, Xiaolian Ning, Xun Lin, Liang Zong, Shanqiao Zheng, Yun Zhao, Jing Wang, Lingyun Chen, Jin Zi, Zhanlong Mei

**Affiliations:** Department of Mass Spectrometry, BGI, No. 146, Beishan Road, Yantian District, Shenzhen 518083, Guangdong Province, China; BGI Center, No. 9, Yunhua Road, Yantian District, Shenzhen 518083, Guangdong Province, China; Department of Mass Spectrometry, BGI, No. 146, Beishan Road, Yantian District, Shenzhen 518083, Guangdong Province, China; Department of Mass Spectrometry, BGI, No. 146, Beishan Road, Yantian District, Shenzhen 518083, Guangdong Province, China; Department of Mass Spectrometry, BGI, No. 146, Beishan Road, Yantian District, Shenzhen 518083, Guangdong Province, China; Department of Mass Spectrometry, BGI, No. 146, Beishan Road, Yantian District, Shenzhen 518083, Guangdong Province, China; Department of Mass Spectrometry, BGI, No. 146, Beishan Road, Yantian District, Shenzhen 518083, Guangdong Province, China; BGI Center, No. 9, Yunhua Road, Yantian District, Shenzhen 518083, Guangdong Province, China; Department of Mass Spectrometry, BGI, No. 146, Beishan Road, Yantian District, Shenzhen 518083, Guangdong Province, China; Department of Mass Spectrometry, BGI, No. 146, Beishan Road, Yantian District, Shenzhen 518083, Guangdong Province, China

**Keywords:** spatial multi-omics, spatial pattern analysis, spatial differential analysis, gene–metabolite co-localization, differential expression network

## Abstract

Current tools for spatial omics analysis often face challenges in performing integrated transcriptomics and metabolomics analysis, in-depth biological interpretation, and user-friendly operation. To address this, we developed SMIntegration, the first web-based graphical platform designed specifically for integrated spatial metabolomics and transcriptomics analysis. Built with R/Shiny and deployed using Docker containerization, the platform provides a complete integration workflow, starting from pre-processed spatial features through to functional annotation. Its core functions include (1) automated and interactive spatial registration; (2) cross-modal spatial pattern recognition; (3) flexible differential analysis of genes and mass features based on clustering results, user-defined regions, or cell type annotations; and (4) group-specific gene–metabolite network construction and interactive visualization. Using adjacent mouse brain coronal sections (Stereo-seq transcriptomics and AFADESI-MS metabolomics) as an example, SMIntegration successfully identified both the periaqueductal gray and subcommissural organ, which were missed by single-modality clustering. Cell type analysis revealed an association between astrocyte-enriched GABA metabolism and *Slc6a11*, while a comparison between the cornu ammonis region and the midbrain periaqueductal gray dissected glutamatergic and endogenous cannabinoid signaling pathway modules. With a zero-code interface, SMIntegration enables a wide range of researchers to deeply explore gene–metabolite interaction mechanisms within microenvironments during development, homeostasis, and disease.

## Introduction

Spatial omics technologies have revolutionized molecular biology by enabling localization of molecular information within tissue sections [1]. Spatial multi-omics, integrating transcriptomics, proteomics, and metabolomics, was recognized by Nature in 2022 as a “Technology to Watch” [2]. Integrating spatial transcriptomics and metabolomics is particularly important as it links gene expression (genotype) with metabolic products (phenotype), revealing mechanisms in development, disease, and therapy. Recent studies highlight this power: Sun et al. [3] profiled gastric cancer metabolic remodeling, while Vicari et al. [4] developed a protocol for simultaneous profiling on a single slice. Such approaches enable spatial clustering comparisons [3, 5], identification of synergistic gene–metabolite modules [4, 6], and cross-region interaction analyses [3, 6]. These insights clarify how spatially defined genes regulate the metabolic microenvironment, advancing understanding of tissue development and disease.

Despite progress, current computational tools face three main limitations. First, modality compatibility is limited: platforms such as SpatialGlue [7] and Giotto [8] mainly address transcriptomics–proteomics, and SpaTrio [9] links single-cell multi-omics with spatial transcriptomics. No standardized pipeline exists for spatial transcriptomics and spatial metabolomics integration. Second, biological interpretation remains shallow. SODB [10] allows data loading but not deep analysis; MIIT [11] supports registration but not interaction studies. Third, accessibility is poor: tools such as MISO [12] and SOAPy [13] require programming expertise, while recent machine learning methods [14] are complex and lack GUIs. These gaps hinder research on how gene regulation shapes spatial metabolism, emphasizing the need for multimodal, analytical, and user-friendly platforms.

To address this, we developed SMIntegration, the first GUI platform for joint spatial metabolomics–transcriptomics analysis. It provides a streamlined downstream integration pipeline, lowering the technical barriers. Core functions include (1) Spatial pattern analysis to identify co-varying features across omics; (2) differential analysis based on clustering, user-defined ROIs, or cell types, with integrated functional annotation; and (3) network analysis and visualization to construct differential expression genes (DEG)/differential abundant mass features (DAM) correlation networks and explore spatial co-localization. Validated on mouse brain data, SMIntegration integrates both modalities, identifies fine brain structures, reveals astrocyte and oligodendrocyte networks, and uncovers mechanisms of synaptic plasticity and pain regulation, demonstrating strong potential for systems-level studies.

## Methods

### Software implementation and architecture

SMIntegration is a web-based GUI implemented in R (v4.4.2) using Shinyproxy and Docker. To ensure the reliability of the integrated analytical workflow, we have implemented a systematic validation protocol. This includes (1) comprehensive inline documentation for all core analytical scripts on GitHub; and (2) a standalone, dual-layer validation suite comprising modular unit tests for core computational modules (e.g., spatial normalization, clustering, and differential expression) and regression tests for figure reproducibility. The complete computational workflow is archived in WorkflowHub [15]. The cloud platform (128 CPUs, 1,000 GB RAM) is available online. Runtime benchmarks are in Supplementary Table S1. For very large datasets, local deployment is recommended. Source code is publicly available, with documentation, tutorials, and example datasets accessible from the help interface (Supplementary Fig. S1).

### Data preparation and spatial registration

SMIntegration is specifically designed as a downstream integration platform. To maintain methodological flexibility and accommodate diverse experimental designs, primary raw data processing—such as peak detection for mass spectrometry imaging or initial analysis for spatial transcriptomics—is handled by specialized external tools. These steps are computationally intensive and highly dependent on instrumentation and user-defined parameters; thus, performing them externally ensures scalability for concurrent users on our cloud-based platform and preserves methodological rigor.

SMIntegration input requires a processed spatial metabolomics and transcriptomics data matrix. Since resolutions differ, higher-resolution data should be aggregated to match the lower (e.g., binning 500 nm transcriptomics by 100 to 50 μm metabolomics) [16]. To facilitate seamless integration, SMIntegration features a dedicated spatial registration module implemented using the RNiftyReg package [17], which provides an R interface to the NiftyReg library [18, 19] and is part of the TractoR framework [20]. This module supports both linear (block-matching) and non-linear (free-form deformation) transformations to align the two modalities. Detailed tutorials for this registration module are provided in Supplementary File 1. Users also have the option to perform coregistration externally using the Python-based SpatialData ecosystem [21]; the aligned data can then be imported into SMIntegration in a compatible format for subsequent integrated analysis. Two input formats are supported: (1) text matrices containing feature name, spatial *x*/*y* coordinates, and values (Supplementary Fig. S2A); (2) Seurat objects with coordinates and abundance in designated slots (Supplementary Fig. S2B). Upload requirements are detailed on the help page.

### Data upload and visualization

**Figure 1:**
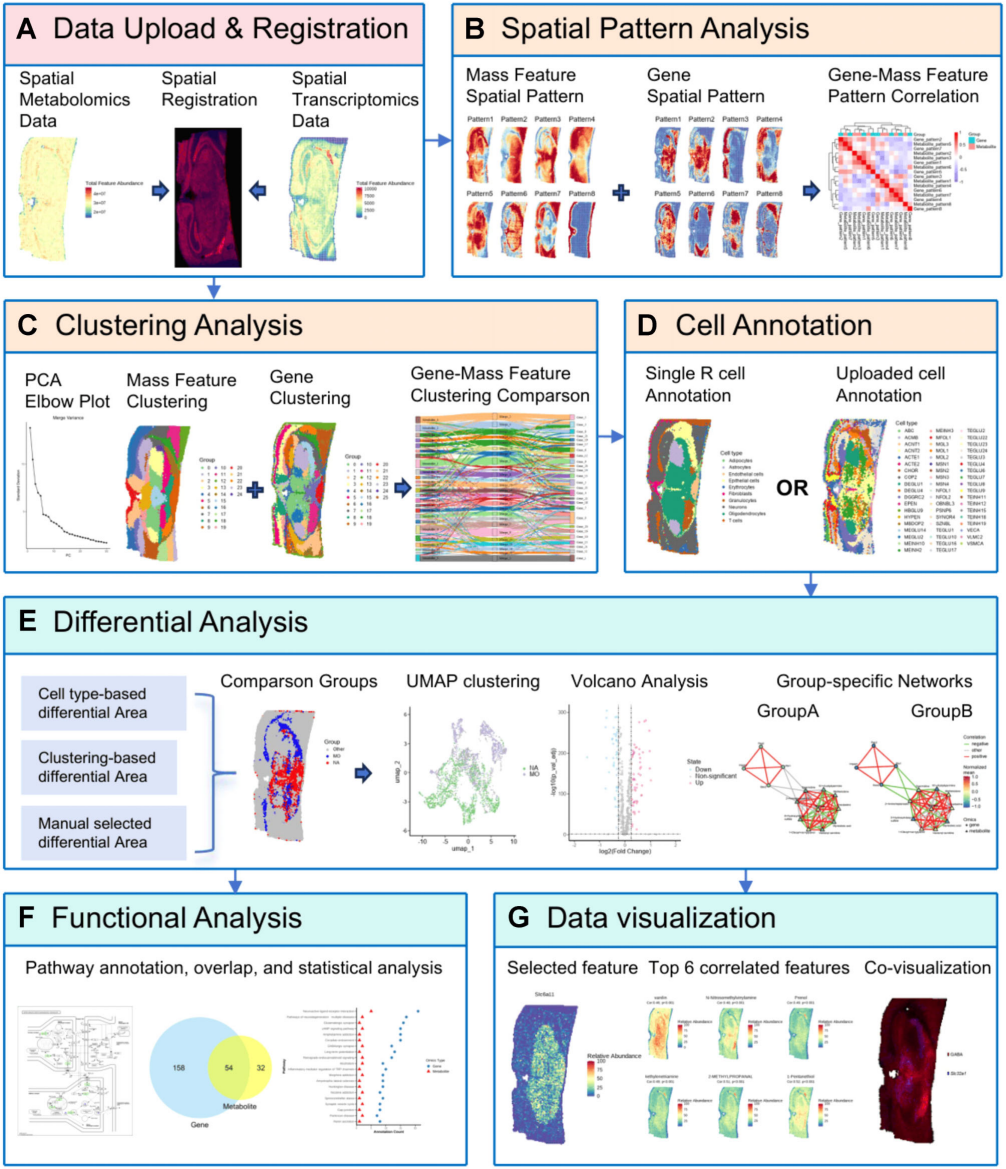
Overall architecture of SMIntegration. (A) Data upload and registration. (B) Spatial pattern analysis, identifying features with specific spatial patterns. (C) Clustering analysis. (D) Cell type annotation analysis. (E) Differential analysis, allowing the selection of corresponding differential features through three different methods. (F) Functional association analysis of differential features. (G) Imaging visualization.

On the Overall Distribution Panel, users can upload datasets or use built-in test data (Supplementary Fig. S3). The system performs format checks, retains overlapping pixels, and generates abundance maps for both omics (Fig. 1A).

### Core analysis modules

#### Spatial expression pattern recognition

This module applies SpaGene [22] to detect spatially variable (SV) features using a model-free, graph-based approach. Specifically, SpaGene constructs a k-nearest neighbor (k-NN) graph based on spatial coordinates and identifies high-expression subnetworks for each gene and metabolite. The spatial pattern strength is then quantified using Earth mover’s distance (EMDg), which measures the distance between the observed high-expression distribution on the spatial graph and a null distribution derived from random permutations. Features with significant EMDg values are identified as SV features. Subsequently, non-negative matrix factorization (NMF) partitions these features into distinct spatial modules based on their pattern similarity (Fig. 1B). To quantify cross-omics associations, the platform employs Moran’s I [23], a measure of spatial autocorrelation, to evaluate the consistency of distribution patterns between identified gene and metabolite modules. Users can browse features within modules (Supplementary Fig. S4).

#### Pixel-level spatial clustering

Five clustering methods are available (Fig. 1C): Louvain (LV), LM, SLM [24], k-means after principal component analysis (PCA), and k-means after Uniform Manifold Approximation and Projection (UMAP) [25, 26]. To assist users in objective parameter selection, PCA is implemented as an initial visualization step to capture major spatial variation across many features. Users can leverage PCA variance plots to estimate the underlying complexity of the spatial domains and thus justify the selection of the number of clusters for subsequent analysis (Supplementary File 2). The pre-processing pipeline follows a standardized workflow to handle the sparsity and technical variance of spatial data: (1) Normalization, where users can choose Total Ion Current (TIC) or root mean squared (RMS) normalization to account for pixel-wise technical variation; (2) transformation, such as LogNormalize to stabilize variance; and (3) scaling and variable feature selection (top 2,000 genes/mass features). This modular design allows users to customize each step or skip them if the input data has been pre-processed. Integrated data combines both modalities by pixel coordinates. This integration algorithm operates by concatenating the pre-processed, scaled feature matrices from both transcriptomics and metabolomics into a unified multimodal matrix. By treating each spatially registered pixel as a shared observation containing both gene and mass feature dimensions, the platform enables joint dimensionality reduction and clustering to uncover synchronized spatial domains. A Sankey diagram compares clustering concordance (Supplementary Fig. S5).

#### Cell type annotation

Cell types are annotated for transcriptomics using SingleR [27] based on reference datasets (MouseRNAseqData, HumanPrimaryCellAtlasData). Since the transcriptomics and metabolomics modalities are spatially registered, SMIntegration transfers these cell-type labels to the corresponding metabolomics pixels based on their overlapping spatial coordinates (Fig. 1D). This allows the definition of cell-type-specific regions of interest (ROIs) for subsequent cross-modal differential analysis. Users may also upload custom annotations (Supplementary Fig. S6).

#### Differential analysis

This analysis consists of two steps: ROI selection and differential testing. ROIs can be defined in three ways (Fig. 1E): interactive selection (manually drawing on metabolomics ion maps or transcriptomics expression maps, Supplementary Fig. S7A), clustering-based selection (Supplementary Fig. S7B), and cell type-based selection (Supplementary Fig. S7C). After defining ROIs, users specify groups (e.g., Region A versus Region B). Each pixel is treated as an independent sample, and Seurat’s FindMarkers function [24] with a Wilcoxon rank-sum test identifies DEGs and DAMs. Results are Bonferroni-corrected [28], with default thresholds of |log2FC| > 0.26 and adjusted *P* < 0.05. The threshold of 0.26 corresponds to a ∼1.2-fold change, which is comparable to standard defaults in pipelines like Seurat. This allows for the detection of subtle molecular gradients and fine-scale heterogeneity in the tissue. Notably, this parameter is fully customizable, allowing users to apply more stringent filters depending on their specific biological questions and sample heterogeneity. In addition to univariate analysis, UMAP visualization highlights expression differences across ROIs, and users can view spatial distributions of identified DEGs or DAMs (Supplementary Fig. S8).

#### Group-specific network construction of differential features

This module reveals spatial co-expression relationships between DEGs and DAMs under specific biological conditions (Fig. 1E). For each comparison group, differential genes and mass features are first selected, and Spearman correlation coefficients are calculated using pixel-level data. Pairs meeting adjusted *P*-value < 0.01 and |*r*| > 0.6 are retained as network edges. By enforcing the same node layout across groups (Supplementary Fig. S9), users can intuitively observe changes in gene–metabolite correlation patterns, facilitating identification of candidate group-specific gene–metabolite association patterns.

#### Functional association and annotation

This module integrates and interprets biological functions of DEGs and DAMs (Fig. 1F). It performs pathway mapping (e.g., to KEGG pathways, version 106.0) and quantifies the number of DEGs and DAMs co-annotated to each pathway. Overly broad global pathways (e.g., “Metabolic pathways”) are excluded. Pathway nodes are color-coded to indicate their up- or down-regulation, allowing users to visualize the spatial distribution of all annotated DEGs and DAMs for any given pathway (Supplementary Fig. S10).

#### Spatial imaging visualization

SMIntegration provides spatial visualization tools for exploring distribution patterns (Fig. 1G). It supports single-feature imaging to generate spatial maps for any gene or metabolite, feature co-localization to display the top six positively and negatively correlated genes and mass features for a selected feature (Supplementary Fig. S11), and multi-feature visualization, where two to three features can be mapped to RGB channels to generate pseudo-color composite images (Supplementary Fig. S12). Together, these functions offer an intuitive means to explore spatial multi-omics relationships.

### Example data and validation

SMIntegration includes datasets from adjacent coronal brain sections of a 7-week-old male mouse. Spatial metabolomics data were acquired by AFADESI-MS (50 μm resolution) and processed with Cardinal [29]. Metabolite identification was performed using the SManalyst platform [30] based on monoisotopic mass matching yielding 13,707 pixels and 560 annotated mass features. It is important to note that this approach constitutes Level 3 annotation according to current metabolomics reporting standards [31]. Therefore, all subsequent references to mass features in the context of these data should be interpreted with caution, as they represent putatively annotated features rather than definitively identified compounds. Spatial transcriptomics data from Stereo-seq were binned to 50 μm, resulting in 14,605 pixels and 10,000 highly variable genes. The two modalities were registered using SpatialData (Supplementary Fig. S13), followed by KNN interpolation and filtering, yielding 14,530 valid pixels. A downsampled demo dataset (500 genes and 500 annotated mass features) is also provided, allowing users to quickly test platform functions via the “Use demo data” option in the Overall Distribution Analysis panel.

## Results and discussion

### Integrated spatial pattern analysis reveals covarying molecular landscapes

**Figure 2:**
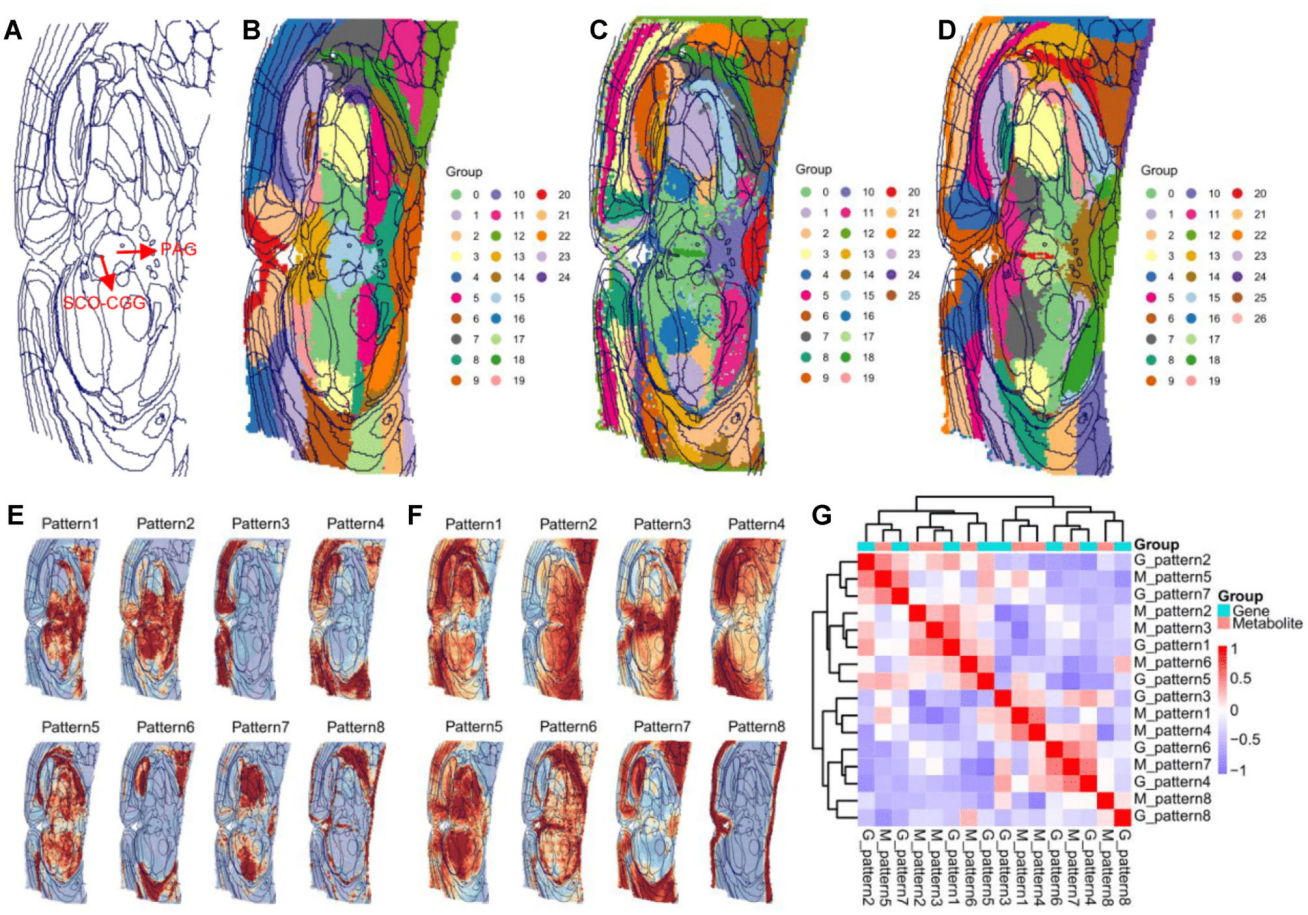
Integrated multi-omics clustering identifies conserved spatial domains and correlated molecular patterns. (A) Schematic of mouse brain structure (atlas from ABA_Mouse_CCFv3_2017_25um.cutlas). (B) Spatial metabolomics clustering. (C) Spatial transcriptomics clustering. (D) Integrated two-omics clustering map. (E) Spatial pattern identification from spatial transcriptomics data. (F) Spatial pattern identification from spatial metabolomics data. (G) Spatial correlation between spatial metabolomics and spatial transcriptomics patterns. (The spatial pattern maps in B–F include brain region outlines for easier identification.)

SMIntegration identifies spatially consistent regions and molecular patterns by offering a variety of clustering algorithms and spatial pattern recognition methods. Joint clustering of integrated spatial metabolomics and transcriptomics data demonstrates improved spatial domain identification. We matched our mouse brain imaging data to the Allen Mouse Brain Reference Atlas [32] using DeepSlice [33] to identify the closest matching atlas plate, and then registered this atlas plate to our experimental images using QuickNII [34], and Fig. 2A shows the brain structure of the mouse after registration. While separate spatial metabolomics clustering could only identify the periaqueductal gray (PAG) (Fig. 2B), and separate transcriptomics clustering could only identify the subcommissural organ (SCO) (Fig. 2C), the joint clustering of both spatial metabolomics and transcriptomics data accurately identified both regions simultaneously (Fig. 2D). This highlights the potential of integrating different modalities to resolve fine spatial heterogeneity. The relationship between the clustering results of different modalities is visualized through a Sankey diagram (Supplementary Fig. S5), which demonstrates the unique information and degree of correspondence contributed by each omics layer to the spatial stratification.

In addition to spatial clustering, SMIntegration uses the SpaGene method to identify molecular expression patterns. Figure 2E and F display the identified spatial expression patterns for genes and mass features, while Fig. 2G shows the correlation between these spatial patterns. Although the overall clustering distributions exhibit similarities, the specific patterns differ. Some patterns appear to be complementary between modalities, such as metabolite pattern 2 and gene pattern 4 (*r* = −0.607). However, conserved cross-modal patterns were also found, such as metabolite pattern 3 and gene pattern 2 (*r* = 0.565), both of which are enriched in the midbrain region (Fig. 2E and F). We performed functional annotation analysis on the conserved pattern pair (Supplementary Fig. S14). The results revealed co-annotation of both patterns in key pathways such as synaptic vesicle cycle and neuroactive ligand-receptor interaction. This finding indicates that the co-localized module is deeply involved in the regulation of synaptic signaling in the midbrain region. It is noteworthy that the cAMP signaling pathway was also annotated. This pathway represents a classic intracellular signaling cascade that translates neurotransmitter receptor activation into changes in neuronal excitability and gene expression and is closely associated with synaptic plasticity [35].

The above analysis demonstrates the powerful capability of the SMIntegration platform in identifying spatially co-localized multi-omic modules. By recognizing coordinated spatial patterns of genes and mass features, it can be directly linked to functional synergy. This finding offers a potential molecular basis for investigating region-specific mechanisms of motor control and reward, highlighting the unique value of integrated spatial multi-omics analysis.

### Cell-specific metabolite analysis

**Figure 3:**
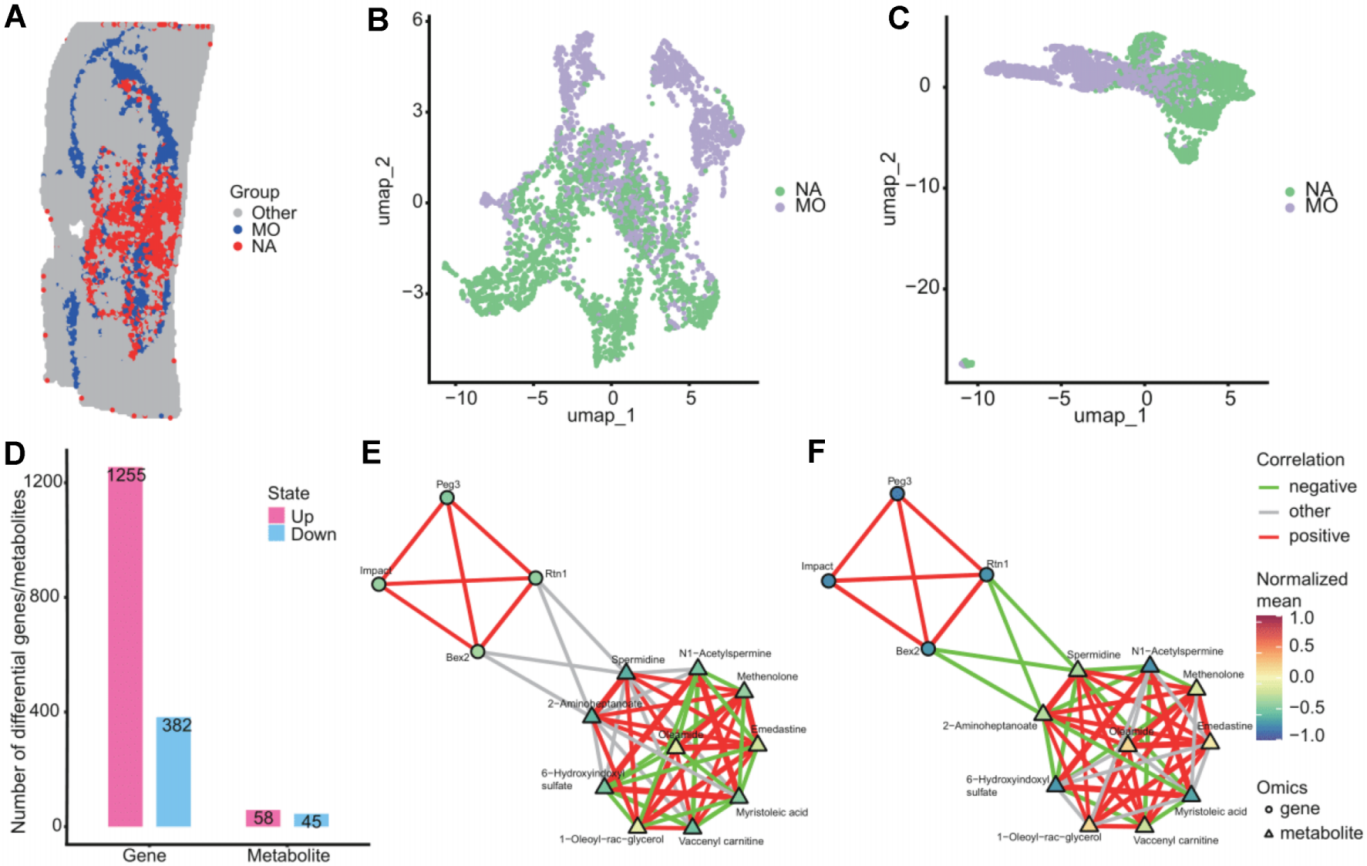
Cell type-based differential analysis reveals cell-enriched mass features and genes. (A) Group comparison selection based on cell types, with the experimental group being non-telencephalon astrocytes (NA) and the control group being mature oligodendrocytes (MO). (B) UMAP analysis of genes in the MO and NA regions. (C) UMAP analysis of mass features in the MO and NA regions. (D) Number of differentially expressed genes and mass features in MO and NA. (E) Correlation network between differential mass features and genes in the NA region. (F) Correlation network between differential mass features and genes in the MO region.

Deciphering gene expression and metabolite abundance changes within specific cell types is crucial for a deeper understanding of cellular function. However, spatial metabolomics data itself lacks direct cell type annotation capabilities. SMIntegration effectively addresses this challenge through its integrated workflow of coordinate-based cell-type assignment and pixel registration. By projecting SingleR-identified labels from transcriptomics onto spatially aligned metabolomics pixels, the platform allows each pixel to be treated as a cell-type-specific sample (Fig. 1D). We demonstrate this by comparing two functionally distinct glial cell populations in the mouse brain: regions dominated by non-telencephalon astrocytes (NA) versus regions dominated by mature oligodendrocytes (MO) (Fig. 3A; Supplementary Fig. S7C). UMAP analysis showed clear differences in metabolite (Fig. 3B) and gene (Fig. 3C) expression between these two cell types.

To systematically pinpoint biological associations from the high-dimensional data, we applied a parallel discovery logic. Through differential screening, SMIntegration identified 103 differential mass features, with 58 upregulated in NA and 45 upregulated in MO. In terms of genes, 1,255 genes were highly expressed in the NA region, while 382 were highly expressed in the MO region (Fig. 3D). To determine the functional synergy between these two lists, the platform’s “Functional Association” module was utilized to perform automated co-annotation analysis. Notably, the gene *Slc6a11* and the metabolite gamma-aminobutyric acid (GABA) were algorithmically flagged as they both converged on the GABAergic synapse pathway (Supplementary Fig. S15A). Both were upregulated in the NA region (Supplementary Fig. S15B and C). *Slc6a11* encodes a sodium-dependent transporter [36], and its absence can lead to GABA accumulation and an imbalance in neuronal excitability, affecting cognitive function [37]. These differential results are consistent with the cellular functions of astrocytes, which play a role in GABA synthesis and transmission.

Cell-type-specific metabolic regulatory networks provide a new perspective for dissecting the mechanisms of metabolic-gene synergistic interactions. We constructed correlation networks for these differential features in the NA and MO regions separately. In the NA region, no significant correlation was observed between *Bex2* and spermidine (Fig. 3E), whereas in the MO region, decreased expression of the *Bex2* gene and upregulation of spermidine were detected, showing a negative correlation between them (Fig. 3F). *Bex2* has anti-apoptotic and pro-proliferative properties [38], while the effect of spermidine on the immune system is dose-dependent, with higher doses being anti-inflammatory and lower doses enhancing cytotoxic immune function [39]. This phenomenon reveals that the correlation between the molecules *Bex2* and spermidine is highly specific to cellular function: The significant negative correlation observed in the MO region, where decreased *Bex2* expression coincides with increased spermidine levels, points to the potential presence of a finely tuned inhibitory relationship—potentially mediated by spermidine accumulation—targeting cell proliferation and apoptosis. In contrast, the lack of correlation in the NA region may indicate a functional decoupling between the two. Such specificity in interaction is likely closely related to the immune microenvironment or neural activity demands of the cell population. These results reflect the heterogeneity of transcriptomics and metabolomics at the cell type level, validating the platform’s utility in precisely dissecting cell-specific molecular networks.

### Differential expression between brain regions reveals functional insights

**Figure 4:**
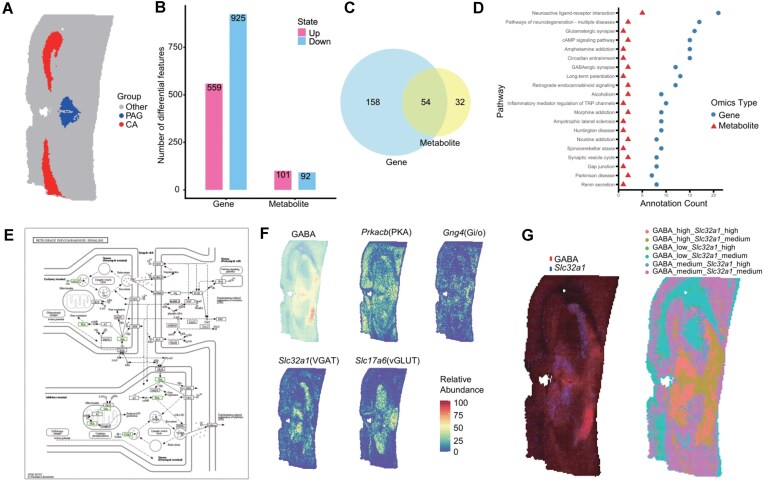
Interactive spatial selection unveils region-specific multi-omics functional pathways. (A) Group comparison selection based on brain regions, with the experimental group as cornu ammonis (CA) and the control group as periaqueductal gray (PAG). (B) Number of differentially expressed genes and mass features in the CA and PAG regions. (C) Number of annotated pathways for differential genes and mass features in the CA and PAG regions. (D) Pathway annotation for differential features in the CA and PAG regions. (E) Pathway Retrograde endocannabinoid signaling annotated with mass features and genes in the PAG region. (F) Abundance distribution maps of enriched genes and mass features in the PAG region. (G) Spatial co-imaging (left) and co-expression (right) of *Slc32a1* and GABA.

Beyond supporting differential analysis based on cell type annotations, SMIntegration’s flexible interactive selection feature (Fig. 1E) also allows researchers to directly select any region of interest to define comparison groups. We used the comparison between the cornu ammonis (CA) region and the midbrain PAG region in a mouse brain coronal section (Supplementary Fig. S16; Fig. 4A) as an example to demonstrate SMIntegration’s flexible differential analysis to reveal regulatory networks in different brain microenvironments. Differential analysis identified 1,484 differential genes (Fig. 4B), corresponding to 212 pathways, and 193 differential mass features, corresponding to 86 pathways. There were 54 pathways shared by both differential genes and mass features (Fig. 4C).

Figure 4D shows the pathways containing these differential features. Genes and mass features highly expressed in the CA region were annotated to pathways such as glutamatergic synapse, neuroactive ligand-receptor interaction, and long-term potentiation/depression. In particular, in the activated glutamatergic synapse pathway (Supplementary Fig. S17A), l-glutamic acid (Supplementary Fig. S17B) and *Grin2a* (Supplementary Fig. S17C) were upregulated. Glutamate is the main excitatory neurotransmitter in the hippocampus, and its metabolic flux directly affects synaptic plasticity. The gene *Grin2a* encodes the NMDAR, an ionotropic glutamate receptor. The synergistic action of these pathways forms the molecular basis of hippocampus-dependent learning and memory [40].

Pathways containing DEGs and DAMs highly expressed in the PAG region point to its core functions: pain modulation and defensive responses. In the addiction-related neuroadaptations pathway (Fig. 4E), both the metabolite GABA and the gene *Slc32a1* were significantly upregulated (Fig. 4F). *Slc32a1* encodes the vesicular transporter VGAT, which loads GABA into synaptic vesicles, and GABA is an inhibitory tonic [41]. Studies have shown that the analgesic effect of exogenous cannabinoids (e.g., Δ9-THC) in the PAG is achieved by activating CB1 receptors to modulate vesicular release patterns and reduce the probability of GABA release [41, 42]. Furthermore, multi-feature imaging and spatial co-expression analysis also demonstrated the consistent high expression of *Slc32a1* and GABA in the midbrain region, predominantly the PAG (Fig. 4G).

## Conclusion

SMIntegration is the first zero-code platform for integrated spatial metabolomics and transcriptomics, unifying spatial pattern recognition, differential analysis, network construction, and functional annotation. It enables intuitive exploration of gene–metabolite interactions and spatial heterogeneity, as demonstrated in the mouse brain by revealing region- and cell type-specific networks and key processes such as GABA/glutamate balance and cannabinoid signaling. Future extensions will include additional omics and machine learning-based modules, further advancing spatial multi-omics research in both fundamental and translational biology.

## Institutional review board statement

The animal study protocol was approved by the Institutional Review Board of BGI (protocol code BGI-IRB A25004 and date of approval 21 February 2025).

## Availability of source code and requirements

Project name: SMIntegration

Project homepage: https://github.com/mzlab-research/SMIntegration

Operating system(s): Platform independent (MacOS, Linux, Windows)

Programming language: R

Other requirements: None

License: MIT license

RRID:SCR_027925

biotoolsID: smintegration

Web Server: https://metax.genomics.cn/app/SMIntegration

Docker Image: https://hub.docker.com/r/mzlabresearch/smintegration

## Additional files

**Supplementary File 1**. Spatial metabolomics and transcriptomics data registration tutorial.

**Supplementary File 2**. Clustering Parameter Selection Tutorial: Using PCA for Data-Driven Clustering in SMIntegration.

**Supplementary Fig. S1**. Screenshot of the SMIntegration tutorial interface.

**Supplementary Fig. S2**. Schematic diagram of data input formats. **A**. Text Matrix Format: Requires metabolomics/transcriptomics data to be submitted as a “feature-pixel” matrix, with each column containing the metabolite/gene name, spatial coordinates (x/y), and feature value. **B**. Seurat Object Format: Requires both spatial metabolomics and transcriptomics data to share identical structures. The feature-pixel matrix is stored in the Spatial$counts slot (rows represent features and columns represent pixels), while spatial coordinates are stored in the meta.data slot.

**Supplementary Fig. S3**. Screenshot of the data upload interface.

**Supplementary Fig. S4**. Screenshot of the spatial pattern analysis interface.

**Supplementary Fig. S5**. Screenshot of the spatial clustering interface.

**Supplementary Fig. S6**. Cell type annotation. **A**. Screenshot of the cell type annotation interface. **B**. Requirements for user-defined annotation file format: Must be a text matrix with three columns: spatial x/y coordinates and cell type.

**Supplementary Fig. S7**. Screenshot of the differential analysis region selection interface. **A**. Use the lasso tool to select ROI, then assign it as the experimental or control group. **B**. Select clustering classes, then assign them as the experimental or control group. **C**. Select cell classes, then assign them as the experimental or control group.

**Supplementary Fig. S8**. Screenshot of the differential analysis interface.

**Supplementary Fig. S9**. Screenshot of the group-specific network interface.

**Supplementary Fig. S10**. Screenshot of the functional association analysis interface.

**Supplementary Fig. S11**. Screenshot of the single-feature visualization interface.

**Supplementary Fig. S12**. Screenshot of the multi-feature visualization interface.

**Supplementary Fig. S13**. Mouse brain data registration workflow. **A**. Total abundance imaging of spatial transcriptomics and spatial metabolomics data before registration. **B**. Total abundance imaging of spatial transcriptomics and spatial metabolomics data after registration.

**Supplementary Fig. S14**. Annotated pathways in the conserved metabolite pattern 3 and gene expression pattern 2.

**Supplementary Fig. S15**. Annotation of enriched features in non-telencephalic astrocytes within the GABAergic synapse pathway. **A**. Molecular annotation in the GABAergic synapse pathway. **B**. The spatial distribution of Gamma-aminobutyric acid (GABA) abundance. **C**. The spatial distribution of *Slc6a11* expression level.

**Supplementary Fig. S16**. Schematic diagram of manual selection for CA vs. PAG.

**Supplementary Fig. S17**. Annotation of enriched features in CA region within the glutamatergic synapse pathway. **A**. Molecular annotation in the glutamatergic synapse pathway. **B**. The spatial distribution of L-Glutamic acid abundance. **C**. The spatial distribution of *Grin2a* expression level.

**Supplementary Table S1**. Execution time and memory consumption across datasets of varying sizes.

## Supplementary Material

giag033_Supplemental_Files

giag033_Authors_Response_To_Reviewer_Comments_original_submission

giag033_GIGA-D-25-00440_original_submission

giag033_GIGA-D-25-00440_revision_1

giag033_Reviewer_1_Report_original_submission Reviewer 1 -- 11/21/2025

giag033_Reviewer_2_Report_original_submission Reviewer 2 -- 12/12/2025

giag033_Reviewer_2_Report_revision_1 Reviewer 2 -- 3/7/2026

giag033_Reviewer_3_Report_original_submission Reviewer 3 -- 12/15/2025

giag033_Reviewer_3_Report_revision_1 Reviewer 3 -- 3/13/2026

## Data Availability

All resources described in this study are publicly available. The raw spatial metabolomics and spatial transcriptomics data from mouse brain tissue, as well as the derived processed peak intensity tables, have been deposited in the China National Center for Bioinformation (CNCB) OMIX database. The spatial metabolomics and transcriptomics data are accessible under accession number OMIX011674 [43]. The processed data is available in GitHub repository [44].
