# Supplementary material for: SMIntegration: A web tool for comprehensive spatial metabolomics and transcriptomics integrated analysis and visualization
Source: Gigascience. 2026 Mar 24;15:giag033. doi: 10.1093/gigascience/giag033 (PMC13159472; doi:10.1093/gigascience/giag033)
Supplement: giag033_Supplemental_Files [file giag033_supplemental_files.zip › Figure_S1.pdf]

SMIntegration: Spatial Multi-omics Integration Platform

SMIntegration is an innovative open-source platform for integrated analysis of spatial transcriptomics and metabolomics data. It addresses critical challenges in spatial multi-omics by providing:

- **Comprehensive integrated spatial analysis:** Joint analysis pipeline from initial visualization to biological interpretation
- **Multimodal pattern discovery:** Multiple algorithms for identifying spatially co-varying regions and gene-metabolite modules
- **Flexible differential analysis:** Comparative analysis supporting multiple region definition strategies
- **Inter-group network comparison:** Construction and comparison of condition-specific correlation networks for differential molecules (DEGs/DAMs)
- **Interactive visualization:** Dynamic exploration of spatial distributions and co-localization patterns

Core Analytical Modules

Overall Distribution Analysis

Initial data visualization of spatial distributions

Spatial Pattern Analysis

Identification of spatially co-varying gene-metabolite modules

Clustering Analysis and Cell Annotation

Spatial domain detection with multiple algorithms and automated cell type mapping

Differential Analysis

DEGs/DAMs discovery and region-specific network construction

Functional Association Analysis

Pathway annotation and cross-omics functional integration

Data Visualization

Advanced spatial imaging including co-localization and multi-feature views

Analysis Workflow

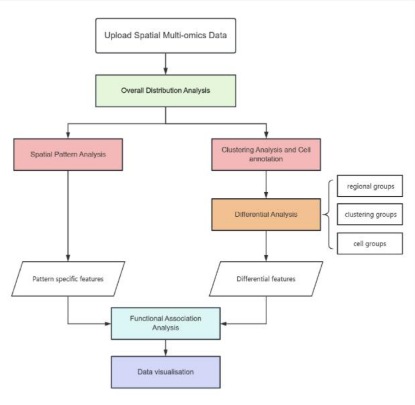

Figure: End-to-end analysis pipeline reflecting the main menu structure

Typical Analysis Workflows

Workflow 1: Comprehensive Spatial Profiling

1. Upload data in **Overall Distribution Analysis**
2. Identify patterns in **Spatial Pattern Analysis**
3. Perform **Clustering and Cell Annotation**
4. Compare regions in **Differential Analysis**
5. Interpret results in **Functional Association Analysis**
6. Visualize findings in **Data Visualization**

Workflow 2: Targeted Region Comparison

1. Define ROIs in **Differential Analysis > Comparison Group Selection**
2. Identify DEGs/DAMs in **Differential Analysis > Differential Screening**
3. Build networks in **Differential Analysis > Group-Specific Network**
4. Interpret results in **Functional Association Analysis**

Demo Datasets

Preprocessed mouse brain data demonstrating key features:

- **Tissue:** Coronal section
- **Transcriptomics:** 500 HVGs from Stereo-seq (0.5μm → 50μm binned)
- **Metabolomics:** 500 HVMs from AFAI-MSI (positive mode)
- **Preprocessing:** Full spatial registration and identification

Data Preparation Requirements

Critical Preprocessing Steps:

- **Resolution Harmonization:** Aggregate higher-resolution data (e.g., bin100 for 500nm→50μm conversion)
- **Spatial Registration:** Two options available:
  - Option A (Built-in Tool): Check 'Perform Registration' in the Upload tab. Supports manual orientation adjustment (Rotate/Flip) followed by automatic alignment, powered by RNiftyReg. Download Registration Tutorial PDF
  - Option B (External Tools): Align coordinates using SpatialData (scripts provided on GitHub).
- **Metabolite Identification:** Requires annotated metabolite names (not m/z values)

Input File Specifications

Option 1: Seurat Object Format (Recommended)

You need to upload two separate Seurat objects (.rds files):

Spatial Metabolomics Object

Component	Content	Slot Name
Assay	Metabolite intensity matrix	Spatial
Meta Data	Spatial coordinates	x, y in meta.data
Active Assay	Must be named 'Spatial'	active.assay

Spatial Transcriptomics Object

Component	Content	Slot Name
Assay	Gene expression matrix	Spatial
Meta Data	Spatial coordinates	x, y in meta.data
Active Assay	Must be named 'Spatial'	active.assay

Download metab Seurat demo | Download trans Seurat demo

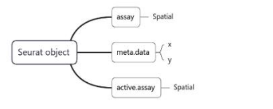

Important Note:

For both formats, the assay name must be 'Spatial' in each Seurat object. The platform expects this naming convention to correctly identify the data modality.

Option 2: Text Matrix Format

You need to upload two separate files:

Spatial Metabolomics File

Column	Description	Example
1	Metabolite Name	metabolite 1
2	X Coordinate	123
3	Y Coordinate	134
4	Intensity	2.0

Spatial Transcriptomics File

Column	Description	Example
1	Gene ID	gene 1
2	X Coordinate	123
3	Y Coordinate	134
4	MIDCount	2

Download demo spatial metabolomics data | Download demo spatial transcriptomics data

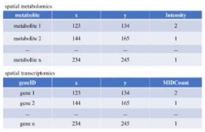

spatial metabolomics

metabolite	x	y	Intensity
metabolite 1	123	134	2
metabolite 2	144	165	1
...	...	...	...
metabolite n	234	245	1

spatial transcriptomics

geneID	x	y	MIDCount
gene 1	123	134	2
gene 2	144	165	1
...	...	...	...
gene n	234	245	1

Additional Resources

Source Code & Local Installation Guide

Technical Support

For immediate assistance:

- **Documentation:** Comprehensive guides available in Help section
- **Email:** denghaoke@genomics.cn
