# Supplementary material for: SMIntegration: A web tool for comprehensive spatial metabolomics and transcriptomics integrated analysis and visualization
Source: Gigascience. 2026 Mar 24;15:giag033. doi: 10.1093/gigascience/giag033 (PMC13159472; doi:10.1093/gigascience/giag033)
Supplement: giag033_Supplemental_Files [file giag033_supplemental_files.zip › Figure_S10.pdf]

Spatial Multi-omics Integration Platform (SMIntegration) ≡

- Tutorial
- Overall Distribution Analysis
- Spatial Pattern Analysis
- Clustering Analysis and Cell Annotation
- Differential Analysis
- Functional Association Analysis
- Data Visualization

Step5: Functional Association Analysis

This module integrates differential or pattern-specific features to elucidate biological mechanisms through three complementary approaches:

1. **Pathway Overlap Analysis:** Count the number of pathways co-annotated by differentially expressed genes/metabolites or pattern-specific genes/metabolites, and those annotated solely by either genes or metabolites.
2. **Pathway Annotation Analysis:** Visualize pathways with the highest number of annotated differential/pattern-specific features.
3. **Pathway-Centric Feature Integration:** Integrate pathway topology with spatial distributions of key molecules

By analyzing pathways co-annotated by spatially significant features, we uncover critical biological mechanisms underlying tissue phenotypes. The platform supports two feature input types:

- **Differential features:** Top 300 DEGs/DAMs (by adjusted p-value) from differential analysis
- **Pattern-specific features:** Top 300 features per spatial module (by correlation score)

Default KEGG annotation is provided. For custom pathways, select 'Upload annotation data'.

Analysis Parameters

Select data for functional association analysis:

Differential features

Select up/down-regulated differential features for analysis

up-regulated and down-regulated features

Annotation source

Built-in KEGG database

Note: Pathway mapping scales with feature count. Please avoid duplicate submissions.

Launch Functional Analysis

Annotation Summary

Export Results

number_of_annotated_pathways	Count_max	Count_min
212	14.00	1.00

Pathway Overlap Analysis

Venn diagram showing shared and modality-specific KEGG pathway annotations.

Pathway Annotation Overlap

Intersection of pathways annotated by input features:

- Left circle: Gene-annotated pathways
- Right circle: Metabolite-annotated pathways
- Overlap: Co-annotated pathways

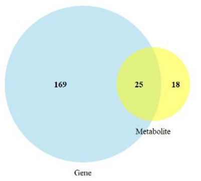

Download image

Pathway Annotation Analysis

Dot plot visualizing pathways with the most mapped features (Top 20 by Count).

Pathway Annotation Count

Filter by pathway type:

Gene-metabolite co-annotated pathways

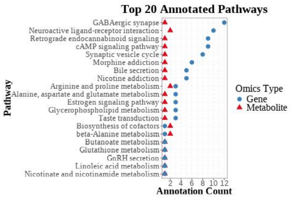

Download image Export data

Pathway-Centric Feature Integration

Co-annotation network mapping of genes and metabolites within biological pathways. Select pathways below to explore:

Pathway Topology Mapping

Annotation type:

Co-annotated pathways

Select pathway:

GABAergic synapse

Download pathway Export features

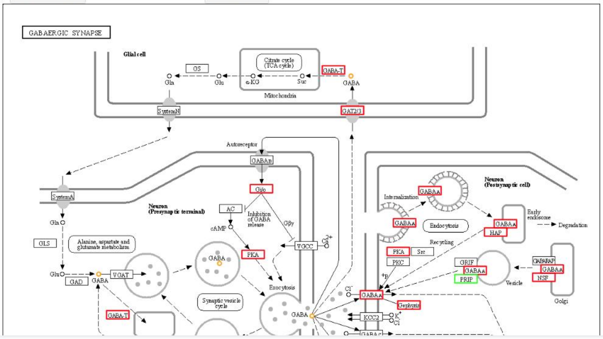

Pathway Feature Table: Metabolites

Key metabolites in selected pathway:

metabolite	KEGG.ID	State	p_val_adj	log2.Fold.Change.
gamma-Aminobutyric acid	C00334	Up	0.00	0.58

Pathway Feature Table: Gene

Key genes in selected pathway:

gene	KEGG.ID	State	p_val_adj	log2.Fold.Change.
Slc6a11	K05044	Up	0.00	1.29
Hap1	K04647	Up	0.00	1.52
Gng3	K04540	Up	0.00	0.87
Gabra1	K05175	Up	0.00	1.01
Nsf	K06027	Up	0.00	0.87
Gphn	K15376	Up	0.00	0.43
Gabrg2	K05186	Up	0.00	0.99
Prkacb	K04345	Up	0.00	0.72
Abat	K13524	Up	0.00	0.65

Spatial Activity Visualization

Examine spatial distributions of key pathway components:

Metabolite Spatial Distribution

Select pathway metabolite:

gamma-Aminobutyric acid

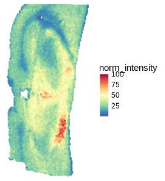

Download image Export data

Gene Spatial Distribution

Select pathway gene:

Slc6a11

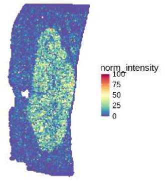

Download image Export data
