# Supplementary material for: SMIntegration: A web tool for comprehensive spatial metabolomics and transcriptomics integrated analysis and visualization
Source: Gigascience. 2026 Mar 24;15:giag033. doi: 10.1093/gigascience/giag033 (PMC13159472; doi:10.1093/gigascience/giag033)
Supplement: giag033_Supplemental_Files [file giag033_supplemental_files.zip › Figure_S11.pdf]

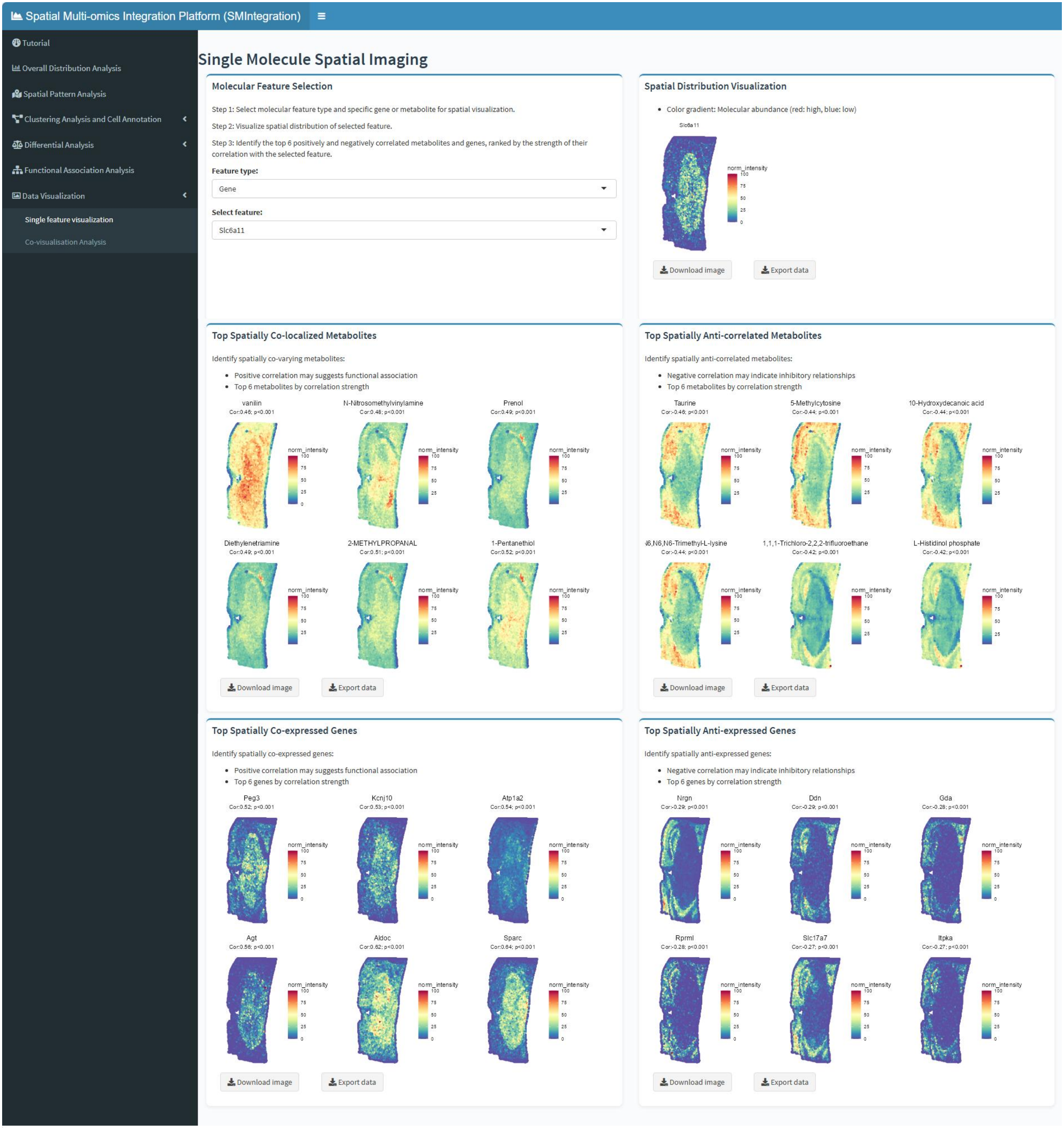
Spatial Multi-omics Integration Platform (SMIntegration)
Tutorial
Overall Distribution Analysis
Spatial Pattern Analysis
Clustering Analysis and Cell Annotation
Differential Analysis
Functional Association Analysis
Data Visualization
Single feature visualization
Co-visualisation Analysis
Single Molecule Spatial Imaging
Molecular Feature Selection
Step 1: Select molecular feature type and specific gene or metabolite for spatial visualization.
Step 2: Visualize spatial distribution of selected feature.
Step 3: Identify the top 6 positively and negatively correlated metabolites and genes, ranked by the strength of their correlation with the selected feature.
Feature type:
Gene
Select feature:
Slc6a11
Spatial Distribution Visualization
Color gradient: Molecular abundance (red: high, blue: low)
Slc6a11
norm_intensity
Download image
Export data
Top Spatially Co-localized Metabolites
Identify spatially co-varying metabolites:
Positive correlation may suggests functional association
Top 6 metabolites by correlation strength
vanilin
Cor:0.46; p<0.001
N-Nitrosomethylvinylamine
Cor:0.48; p<0.001
Prenol
Cor:0.49; p<0.001
Diethylenetriamine
Cor:0.49; p<0.001
2-METHYLPROPANAL
Cor:0.51; p<0.001
1-Pentanethiol
Cor:0.52; p<0.001
Download image
Export data
Top Spatially Anti-correlated Metabolites
Identify spatially anti-correlated metabolites:
Negative correlation may indicate inhibitory relationships
Top 6 metabolites by correlation strength
Taurine
Cor:-0.46; p<0.001
5-Methylcytosine
Cor:-0.44; p<0.001
10-Hydroxydecanoic acid
Cor:-0.44; p<0.001
N6,N6,N6-Trimethyl-L-lysine
Cor:-0.44; p<0.001
1,1,1-Trichloro-2,2,2-trifluoroethane
Cor:-0.42; p<0.001
L-Histidinol phosphate
Cor:-0.42; p<0.001
Download image
Export data
Top Spatially Co-expressed Genes
Identify spatially co-expressed genes:
Positive correlation may suggests functional association
Top 6 genes by correlation strength
Peg3
Cor:0.52; p<0.001
Kcnj10
Cor:0.53; p<0.001
Atp1a2
Cor:0.54; p<0.001
Agt
Cor:0.56; p<0.001
Aldoc
Cor:0.62; p<0.001
Sparc
Cor:0.64; p<0.001
Download image
Export data
Top Spatially Anti-expressed Genes
Identify spatially anti-expressed genes:
Negative correlation may indicate inhibitory relationships
Top 6 genes by correlation strength
Nrgn
Cor:-0.29; p<0.001
Ddn
Cor:-0.29; p<0.001
Gda
Cor:-0.28; p<0.001
Rprml
Cor:-0.28; p<0.001
Slc17a7
Cor:-0.27; p<0.001
Itpka
Cor:-0.27; p<0.001
Download image
Export data
