# Supplementary material for: SMIntegration: A web tool for comprehensive spatial metabolomics and transcriptomics integrated analysis and visualization
Source: Gigascience. 2026 Mar 24;15:giag033. doi: 10.1093/gigascience/giag033 (PMC13159472; doi:10.1093/gigascience/giag033)
Supplement: giag033_Supplemental_Files [file giag033_supplemental_files.zip › Figure_S12.pdf]

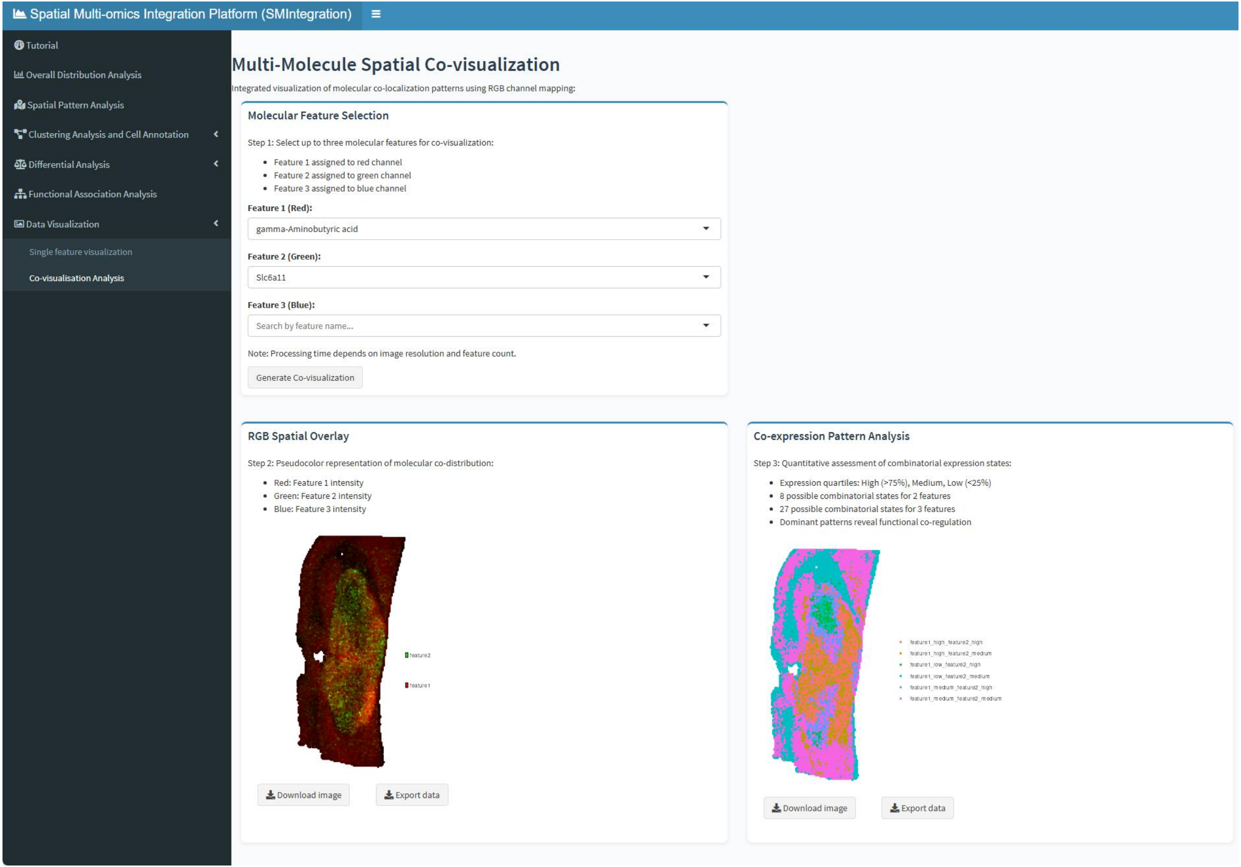
Spatial Multi-omics Integration Platform (SMIntegration)
Tutorial
Overall Distribution Analysis
Spatial Pattern Analysis
Clustering Analysis and Cell Annotation
Differential Analysis
Functional Association Analysis
Data Visualization
Single feature visualization
Co-visualisation Analysis
Multi-Molecule Spatial Co-visualization
Integrated visualization of molecular co-localization patterns using RGB channel mapping:
Molecular Feature Selection
Step 1: Select up to three molecular features for co-visualization:
Feature 1 assigned to red channel
Feature 2 assigned to green channel
Feature 3 assigned to blue channel
Feature 1 (Red):
gamma-Aminobutyric acid
Feature 2 (Green):
Slc6a11
Feature 3 (Blue):
Search by feature name...
Note: Processing time depends on image resolution and feature count.
Generate Co-visualization
RGB Spatial Overlay
Step 2: Pseudocolor representation of molecular co-distribution:
Red: Feature 1 intensity
Green: Feature 2 intensity
Blue: Feature 3 intensity
feature2
feature1
Download image
Export data
Co-expression Pattern Analysis
Step 3: Quantitative assessment of combinatorial expression states:
Expression quartiles: High (>75%), Medium, Low (<25%)
8 possible combinatorial states for 2 features
27 possible combinatorial states for 3 features
Dominant patterns reveal functional co-regulation
feature1_high_feature2_high
feature1_high_feature2_medium
feature1_low_feature2_high
feature1_low_feature2_medium
feature1_medium_feature2_high
feature1_medium_feature2_medium
Download image
Export data
