# Supplementary material for: SMIntegration: A web tool for comprehensive spatial metabolomics and transcriptomics integrated analysis and visualization
Source: Gigascience. 2026 Mar 24;15:giag033. doi: 10.1093/gigascience/giag033 (PMC13159472; doi:10.1093/gigascience/giag033)
Supplement: giag033_Supplemental_Files [file giag033_supplemental_files.zip › Figure_S16.pdf]

Spatial Multi-omics Integration Platform (SMIntegration)

- Tutorial
- Overall Distribution Analysis
- Spatial Pattern Analysis
- Clustering Analysis and Cell Annotation
- Differential Analysis
  - » Comparison Group Selection
  - » Differential Screening and Visualization
  - » Group-Specific Network
- Functional Association Analysis
- Data Visualization

Step4: Differential Analysis

Region of Interest (ROI) Definition

Define spatial regions for comparative analysis using three flexible approaches:

1. **Cluster-based:** Select domains from spatial clustering results
2. **Cell type-based:** Leverage annotated cell types for region definition
3. **Interactive selection:** Manually delineate regions using lasso or rectangular tools

After defining ROIs, click 'Add as treatment group' or 'Add as control group'. Use 'Clear selection' to reset. Finalize with 'Finish Selection' for downstream analysis.

ROI Selection Parameters

ROI definition method:

Cluster-based

Base visualization modality:

Merge

Define regions and assign to treatment and control groups:

Select clusters as treatment group:

1 26

Select clusters as control group:

17

Clear selection | Finish selection

ROI Assignment Summary

Group	Region	X_Min	X_Max	Y_Min	Y_Max	Point_Count
treatment	Cluster_1_26	92.00	119.00	65.00	244.00	1158
control	Cluster_17	109.00	133.00	141.00	174.00	419

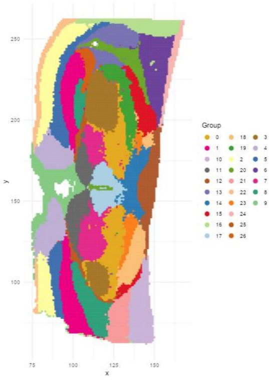

Spatial Region Assignment

Final ROI assignment for differential analysis:

- **Treatment:** Experimental regions (e.g., disease foci)
- **Control:** Reference regions (e.g., healthy tissue)

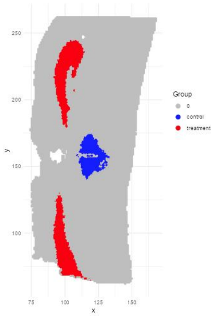

Export ROI assignments | Download image
