# Supplementary material for: SMIntegration: A web tool for comprehensive spatial metabolomics and transcriptomics integrated analysis and visualization
Source: Gigascience. 2026 Mar 24;15:giag033. doi: 10.1093/gigascience/giag033 (PMC13159472; doi:10.1093/gigascience/giag033)

A

spatial metabolomics

metabolite	x	y	Intensity
metabolite 1	123	134	2
metabolite 2	144	165	1
...	...	...	...
metabolite n	234	245	1

spatial transcriptomics

geneID	x	y	MIDCount
gene 1	123	134	2
gene 2	144	165	1
...	...	...	...
gene n	234	245	1

B

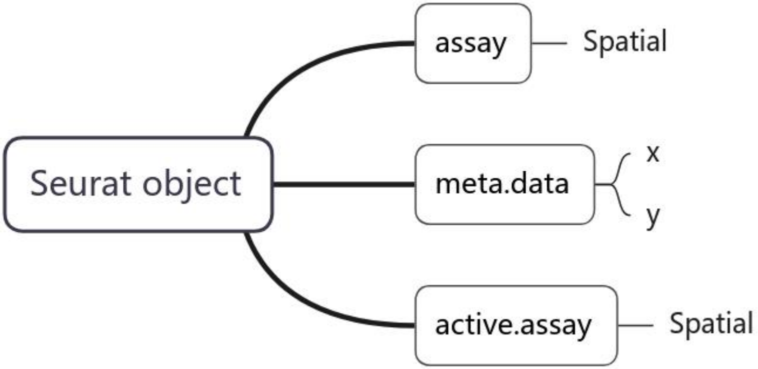

Supplement: giag033_Supplemental_Files [file giag033_supplemental_files.zip › Figure_S2.pdf]
