# Supplementary material for: SMIntegration: A web tool for comprehensive spatial metabolomics and transcriptomics integrated analysis and visualization
Source: Gigascience. 2026 Mar 24;15:giag033. doi: 10.1093/gigascience/giag033 (PMC13159472; doi:10.1093/gigascience/giag033)
Supplement: giag033_Supplemental_Files [file giag033_supplemental_files.zip › Figure_S3.pdf]

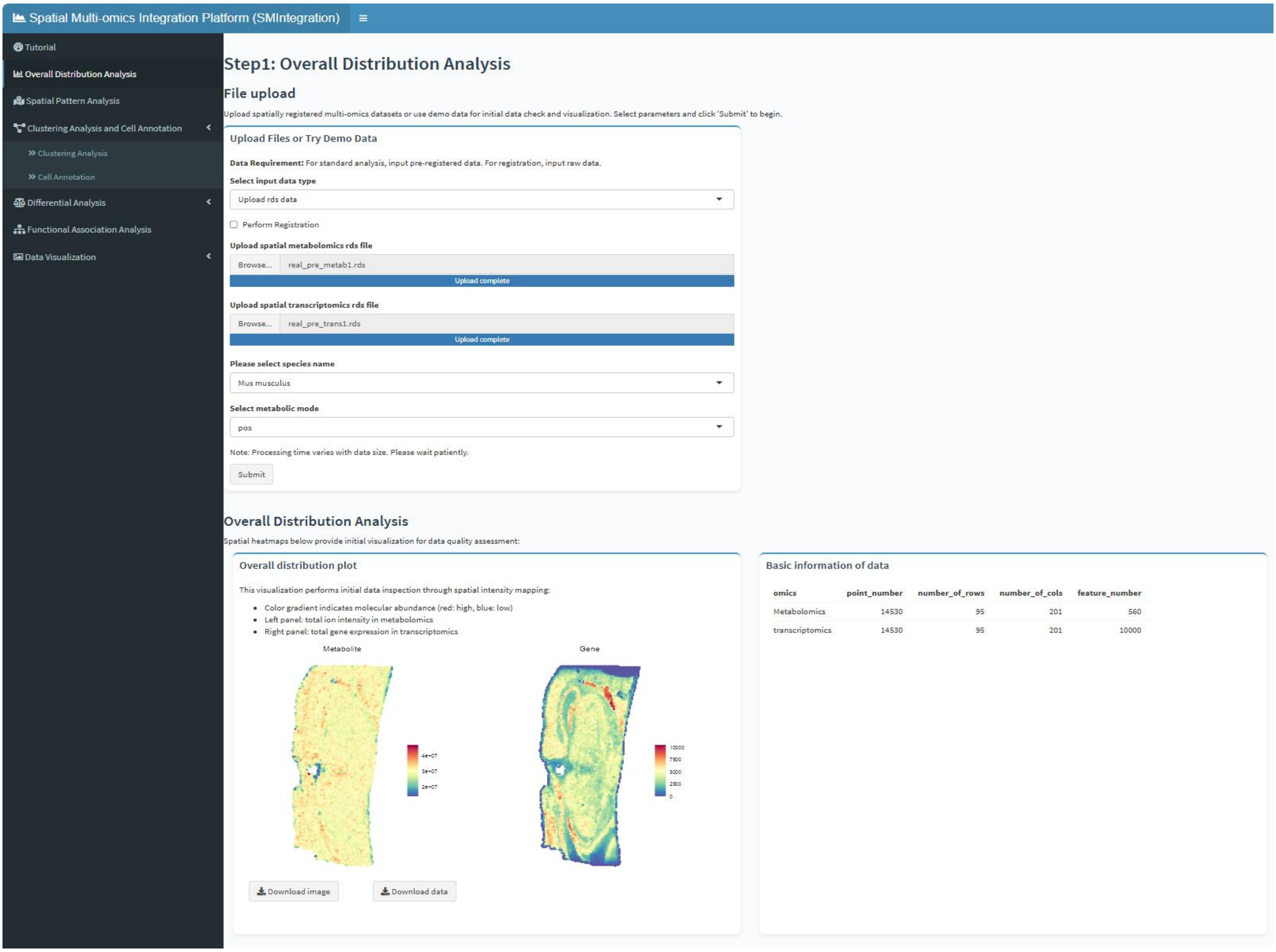
Spatial Multi-omics Integration Platform (SMIntegration)
Tutorial
Overall Distribution Analysis
Spatial Pattern Analysis
Clustering Analysis and Cell Annotation
Clustering Analysis
Cell Annotation
Differential Analysis
Functional Association Analysis
Data Visualization
Step1: Overall Distribution Analysis
File upload
Upload spatially registered multi-omics datasets or use demo data for initial data check and visualization. Select parameters and click 'Submit' to begin.
Upload Files or Try Demo Data
Data Requirement: For standard analysis, input pre-registered data. For registration, input raw data.
Select input data type
Upload rds data
Perform Registration
Upload spatial metabolomics rds file
Browse...
real_pre_metab1.rds
Upload complete
Upload spatial transcriptomics rds file
Browse...
real_pre_trans1.rds
Upload complete
Please select species name
Mus musculus
Select metabolic mode
pos
Note: Processing time varies with data size. Please wait patiently.
Submit
Overall Distribution Analysis
Spatial heatmaps below provide initial visualization for data quality assessment:
Overall distribution plot
This visualization performs initial data inspection through spatial intensity mapping:
Color gradient indicates molecular abundance (red: high, blue: low)
Left panel: total ion intensity in metabolomics
Right panel: total gene expression in transcriptomics
Metabolite
4e+07
3e+07
2e+07
Gene
10000
7500
5000
2500
0
Download image
Download data
Basic information of data
omics point_number number_of_rows number_of_cols feature_number
Metabolomics 14530 95 201 560
transcriptomics 14530 95 201 10000
