# Supplementary material for: SMIntegration: A web tool for comprehensive spatial metabolomics and transcriptomics integrated analysis and visualization
Source: Gigascience. 2026 Mar 24;15:giag033. doi: 10.1093/gigascience/giag033 (PMC13159472; doi:10.1093/gigascience/giag033)
Supplement: giag033_Supplemental_Files [file giag033_supplemental_files.zip › Figure_S4.pdf]

Spatial Multi-omics Integration Platform (SMIntegration)

- Tutorial
- Overall Distribution Analysis
- Spatial Pattern Analysis
- Clustering Analysis and Cell Annotation
- Differential Analysis
- Functional Association Analysis
- Data Visualization

Step2: Spatial Pattern Analysis

This module employs the SpaGene algorithm to identify spatially co-varying molecular modules and discover cross-modal pattern correlations.

- Step 1: Construct k-nearest neighbor spatial networks for each omics layer
- Step 2: Identify spatially variable features by comparing actual Earth mover's distance (EMDg) against a null distribution from 500 random permutations
- Step 3: Cluster significant features into spatial modules
- Step 4: Quantify cross-omics pattern correlations using Moran's I (spdep)
- Step 5: Screen pattern-specific features

Click 'Start spatial pattern analysis' to initiate the workflow.

Note: Computational time varies with feature count. Please avoid duplicate submissions.

Start spatial pattern analysis

Spatial Pattern Modules

Identified spatial pattern modules for metabolites (left) and genes (right).

Metabolite Spatial Pattern Modules

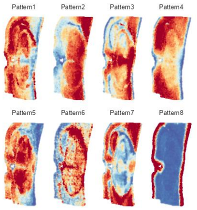

Download image

Gene Spatial Pattern Modules

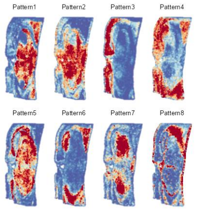

Download image

Cross-Omics Pattern Correlation

Moran's I correlation matrix quantifying spatial co-variation between metabolite and gene pattern modules. Significance levels: *p<0.05, **p<0.01, ***p<0.001.

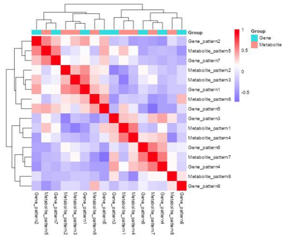

Download image Export correlation matrix

Pattern-Specific Feature Classification

Features are assigned to spatial modules based on spatial correlation:

- Correlation ≥0.5: Assigned to best-matching module
- Correlation <0.5: Categorized as 'unclassified'

Metabolite Distribution Across Spatial Modules

Barplot showing metabolite counts per spatial pattern module after correlation thresholding.

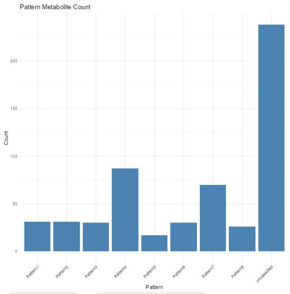

Download image Export metabolite assignments

Gene Distribution Across Spatial Modules

Barplot showing gene counts per spatial pattern module after correlation thresholding.

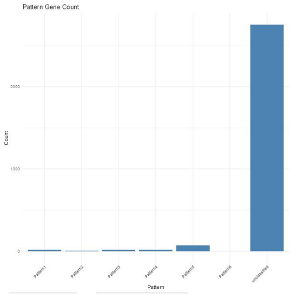

Download image Export gene assignments

Spatial Distribution of Pattern-Specific Features

Visualize spatial expression patterns of top module-associated features. Select modules and features below:

The top 300 features per module (ranked by correlation) are designated as 'pattern-specific features' for functional analysis.

Metabolite Spatial Distribution

Select metabolite spatial module:

Pattern3

Select metabolite:

gamma-Aminobutyric acid

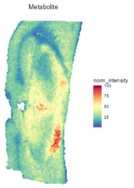

Download image Export intensity data

Gene Spatial Distribution

Select gene spatial module:

Pattern2

Select gene:

Slc6a11

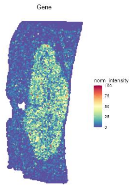

Download image Export expression data
