# Supplementary material for: SMIntegration: A web tool for comprehensive spatial metabolomics and transcriptomics integrated analysis and visualization
Source: Gigascience. 2026 Mar 24;15:giag033. doi: 10.1093/gigascience/giag033 (PMC13159472; doi:10.1093/gigascience/giag033)
Supplement: giag033_Supplemental_Files [file giag033_supplemental_files.zip › Figure_S5.pdf]

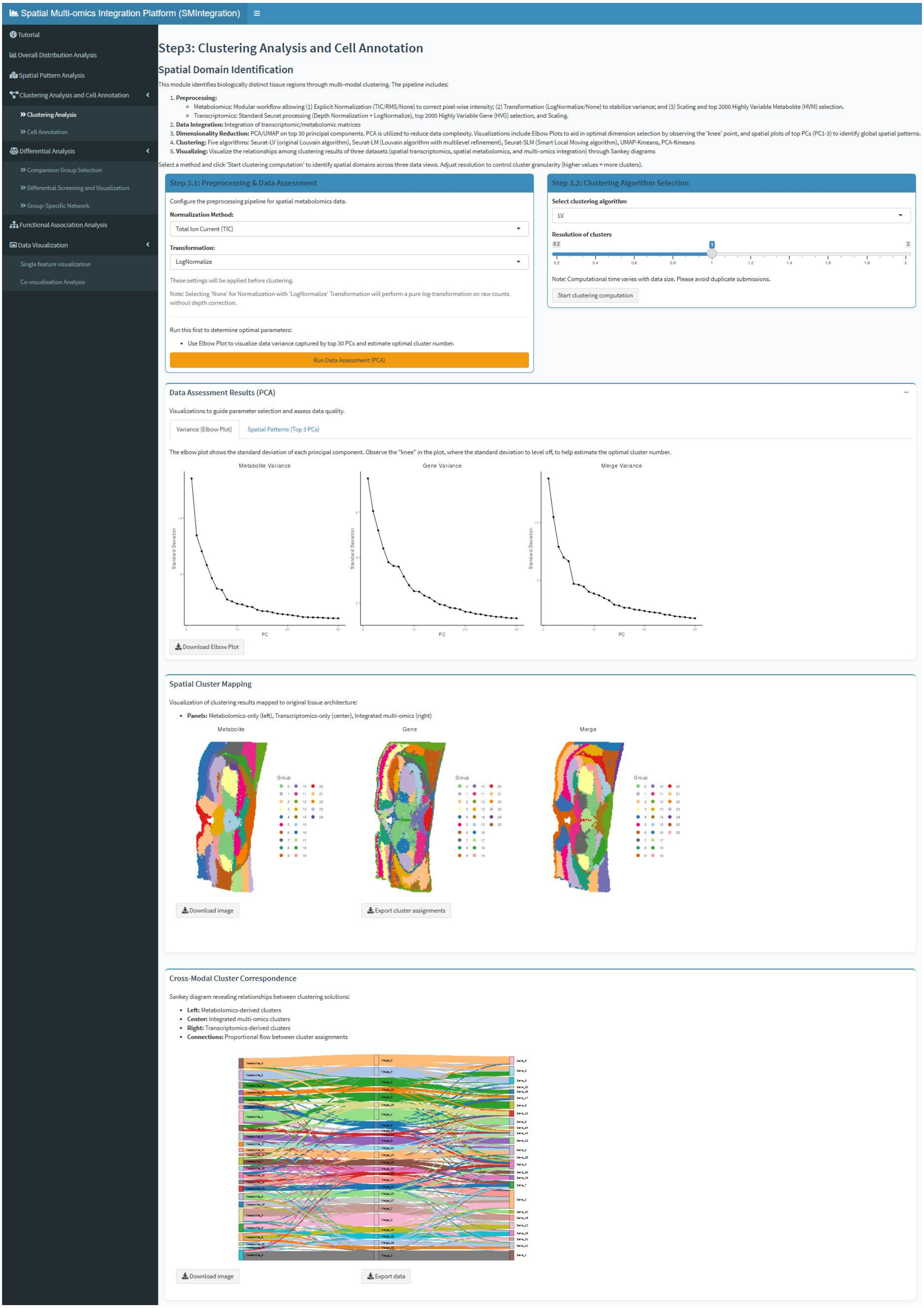

Spatial Multi-omics Integration Platform (SMIntegration)
Tutorial
Overall Distribution Analysis
Spatial Pattern Analysis
Clustering Analysis and Cell Annotation
Clustering Analysis
Cell Annotation
Differential Analysis
Comparison Group Selection
Differential Screening and Visualization
Group-Specific Network
Functional Association Analysis
Data Visualization
Single feature visualization
Co-visualisation Analysis
Step3: Clustering Analysis and Cell Annotation
Spatial Domain Identification
This module identifies biologically distinct tissue regions through multi-modal clustering. The pipeline includes:
1. Preprocessing:
Metabolomics: Modular workflow allowing (1) Explicit Normalization (TIC/RMS/None) to correct pixel-wise intensity; (2) Transformation (LogNormalize/None) to stabilize variance; and (3) Scaling and top 2000 Highly Variable Metabolite (HVM) selection.
Transcriptomics: Standard Seurat processing (Depth Normalization + LogNormalize), top 2000 Highly Variable Gene (HVG) selection, and Scaling.
2. Data Integration: Integration of transcriptomic/metabolomic matrices
3. Dimensionality Reduction: PCA/UMAP on top 30 principal components. PCA is utilized to reduce data complexity. Visualizations include Elbow Plots to aid in optimal dimension selection by observing the 'knee' point, and spatial plots of top PCs (PC1-3) to identify global spatial patterns.
4. Clustering: Five algorithms: Seurat-LV (original Louvain algorithm), Seurat-LM (Louvain algorithm with multilevel refinement), Seurat-SLM (Smart Local Moving algorithm), UMAP-Kmeans, PCA-Kmeans
5. Visualizing: Visualize the relationships among clustering results of three datasets (spatial transcriptomics, spatial metabolomics, and multi-omics integration) through Sankey diagrams
Select a method and click 'Start clustering computation' to identify spatial domains across three data views. Adjust resolution to control cluster granularity (higher values = more clusters).
Step 3.1: Preprocessing & Data Assessment
Configure the preprocessing pipeline for spatial metabolomics data.
Normalization Method:
Total Ion Current (TIC)
Transformation:
LogNormalize
These settings will be applied before clustering.
Note: Selecting 'None' for Normalization with 'LogNormalize' Transformation will perform a pure log-transformation on raw counts without depth correction.
Run this first to determine optimal parameters:
Use Elbow Plot to visualize data variance captured by top 30 PCs and estimate optimal cluster number.
Run Data Assessment (PCA)
Step 3.2: Clustering Algorithm Selection
Select clustering algorithm
LV
Resolution of clusters
0.2
1
2
0.2 0.4 0.6 0.8 1 1.2 1.4 1.6 1.8 2
Note: Computational time varies with data size. Please avoid duplicate submissions.
Start clustering computation
Data Assessment Results (PCA)
Visualizations to guide parameter selection and assess data quality.
Variance (Elbow Plot)
Spatial Patterns (Top 3 PCs)
The elbow plot shows the standard deviation of each principal component. Observe the "knee" in the plot, where the standard deviation to level off, to help estimate the optimal cluster number.
Metabolite Variance
Gene Variance
Merge Variance
Standard Deviation
PC
Download Elbow Plot
Spatial Cluster Mapping
Visualization of clustering results mapped to original tissue architecture:
Panels: Metabolomics-only (left), Transcriptomics-only (center), Integrated multi-omics (right)
Metabolite
Gene
Merge
Group
Download image
Export cluster assignments
Cross-Modal Cluster Correspondence
Sankey diagram revealing relationships between clustering solutions:
Left: Metabolomics-derived clusters
Center: Integrated multi-omics clusters
Right: Transcriptomics-derived clusters
Connections: Proportional flow between cluster assignments
Download image
Export data
