# Supplementary material for: SMIntegration: A web tool for comprehensive spatial metabolomics and transcriptomics integrated analysis and visualization
Source: Gigascience. 2026 Mar 24;15:giag033. doi: 10.1093/gigascience/giag033 (PMC13159472; doi:10.1093/gigascience/giag033)
Supplement: giag033_Supplemental_Files [file giag033_supplemental_files.zip › Figure_S8.pdf]

Spatial Multi-omics Integration Platform (SMIntegration)

- Tutorial
- Overall Distribution Analysis
- Spatial Pattern Analysis
- Clustering Analysis and Cell Annotation
- Differential Analysis
  - Comparison Group Selection
  - Differential Screening and Visualization
  - Group-Specific Network
- Functional Association Analysis
- Data Visualization

Step4: Differential Analysis

Differential Feature Identification

Identify differentially expressed genes (DEGs) and differentially abundant metabolites (DAMs) using Seurat's FindMarkers function:

- Wilcoxon rank-sum test with Bonferroni correction
- Thresholds: |log2FC| > 0.26 & adjusted p-value < 0.05
- Pixel-level analysis treating each spatial spot as independent sample

Set thresholds below and click 'Start differential screening'.

Metabolomics |log2FC| threshold:

0.26

Transcriptomics |log2FC| threshold:

0.26

Metabolomics FDR threshold:

0.05

Transcriptomics FDR threshold:

0.05

Note: Computation scales with feature count. Please avoid duplicate submissions.

Start differential screening

Differential Feature Summary

Barplot showing significantly upregulated (red) and downregulated (blue) features:

- Height indicates feature counts
- Annotations show total differential features per modality

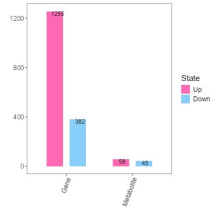

Download image | Export differential features

Differential Feature Characterization

Multi-dimensional visualization of differential features:

UMAP: Metabolite

- Points represent spatial spots
- Color indicates treatment (red) vs control (blue) groups
- Separation distance reflects group dissimilarity

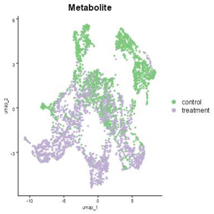

Download image | Export coordinates

UMAP: Gene

- Points represent spatial spots
- Color indicates treatment (red) vs control (blue) groups
- Separation distance reflects group dissimilarity

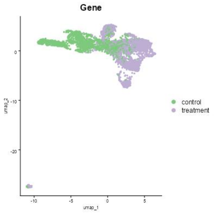

Download image | Export coordinates

Volcano Plot: Metabolite

- X-axis: log2 fold change (effect size)
- Y-axis: -log10(adjusted p-value) (significance)
- Red: significantly upregulated features
- Blue: significantly downregulated features

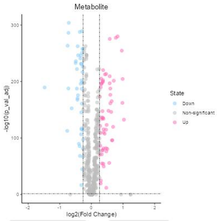

Download image | Export data

Volcano Plot: Gene

- X-axis: log2 fold change (effect size)
- Y-axis: -log10(adjusted p-value) (significance)
- Red: significantly upregulated features
- Blue: significantly downregulated features

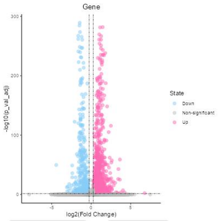

Download image | Export data

Spatial Distribution of Differential Features

Examine spatial expression patterns of significant features:

Differential Metabolite Distribution

Select DAM to visualize:

gamma-Aminobutyric acid

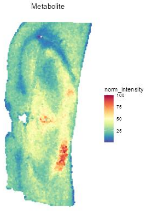

Download image | Export intensity data

Differential Gene Distribution

Select DEG to visualize:

Slc6a11

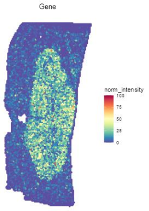

Download image | Export expression data
