# Supplementary material for: SMIntegration: A web tool for comprehensive spatial metabolomics and transcriptomics integrated analysis and visualization
Source: Gigascience. 2026 Mar 24;15:giag033. doi: 10.1093/gigascience/giag033 (PMC13159472; doi:10.1093/gigascience/giag033)
Supplement: giag033_Supplemental_Files [file giag033_supplemental_files.zip › Figure_S9.pdf]

Spatial Multi-omics Integration Platform (SMIntegration) ≡

- Tutorial
- Overall Distribution Analysis
- Spatial Pattern Analysis
- Clustering Analysis and Cell Annotation
- Differential Analysis
  - » Comparison Group Selection
  - » Differential Screening and Visualization
  - » Group-Specific Network
- Functional Association Analysis
- Data Visualization

# Step4: Differential Analysis

## Group-Specific Correlation Networks

Reveal spatial co-expression relationships between top differential features:

- Constructs separate networks for each group
- Computes Spearman correlations using group-specific pixels
- Multiple testing correction: Applies Bonferroni adjustment to p-values
- Filters: |r| > 0.6 & adjusted p-value < 0.01

By default, top 20 differential features per modality (by p-value) are included.

**Correlation threshold (|r|):**

0.5

**adjusted p-value threshold:**

0.05

**Features per modality:**

10

**Network layout:**

Kamada-Kawai (kk)

**Display node labels:**

yes

**Node size:**

6

**Label size:**

3

### Treatment Group Network

- **Edges:** Red: positive correlation, Green: negative correlation
- **Nodes Shapes:** Triangles: metabolites, Circles: genes
- **Node Colors:** Feature abundance gradient (blue-low to red-high)

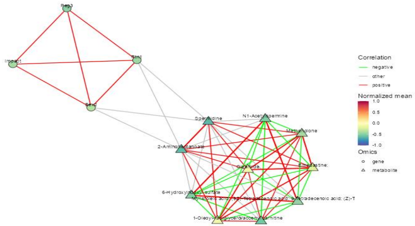


Download image

Export network

### Control Group Network

- Identical node layout for direct comparison
- Edge differences indicate altered regulatory relationships
- Node color differences reflect abundance changes

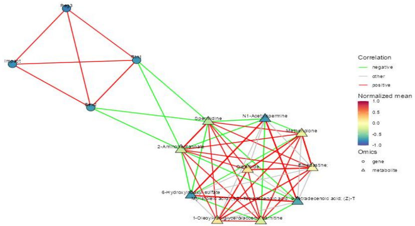


Download image

Export network
