# Supplementary material for: SMIntegration: A web tool for comprehensive spatial metabolomics and transcriptomics integrated analysis and visualization
Source: Gigascience. 2026 Mar 24;15:giag033. doi: 10.1093/gigascience/giag033 (PMC13159472; doi:10.1093/gigascience/giag033)
Supplement: giag033_Supplemental_Files [file giag033_supplemental_files.zip › Supplementary_File_1.pdf]

Spatial Registration Module Tutorial

This tutorial guides you through the process of spatially registering your metabolomics and transcriptomics data within the SMIntegration platform. Accurate registration is crucial for integrating multi-omics data and ensuring meaningful downstream analysis.

Overview of the Registration Process

The registration module allows for both manual orientation adjustments (flipping, rotation, translation) and automatic intensity-based alignment using RNiftyReg.

Step-by-Step Guide

Step 1: Navigate to the Upload Tab

1. Open the SMIntegration application.

   Navigate to the **"Overall Distribution Analysis"** module in the sidebar, and then find the **"Upload"** sub-tab.

   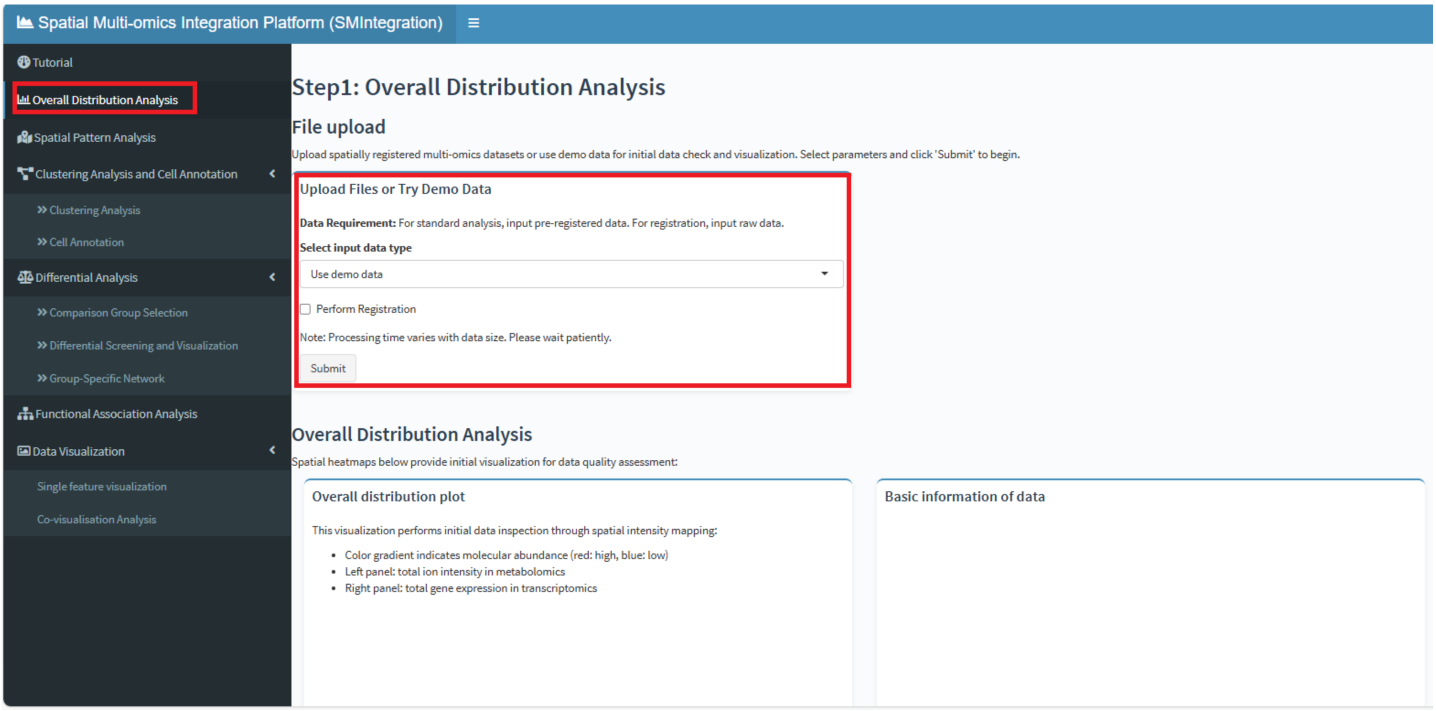

   Screenshot: SMIntegration interface with the "Upload" tab highlighted.

Step 2: Enable Registration

1. Within the "Upload" tab, locate the **"Perform Registration"** checkbox.

Check this box to enable the spatial registration functionalities. This will reveal the registration controls and preview panels.

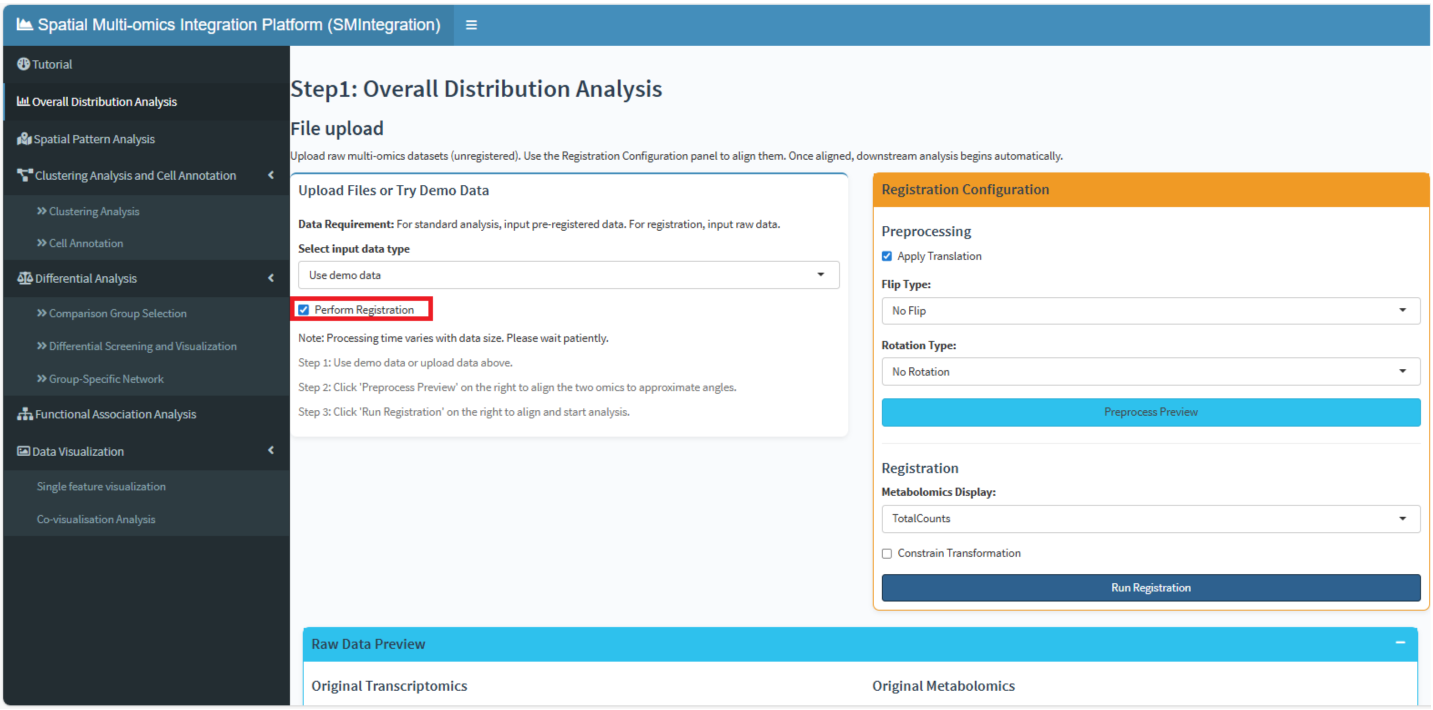

Screenshot: "Upload" tab with "Perform Registration" checkbox checked.

Step 3: Upload Your Data or Select Demo Data

For your own data:

- Choose your data format (e.g., "Upload txt data" or "Upload rds data") from the "Data Selection" dropdown.
- Use the file input fields (`Upload spatial metabolomics...` and `Upload spatial transcriptomics...`) to upload your metabolomics and transcriptomics files.
- After uploading your files, the "Raw Data Preview" will automatically update.

For demo data:

- Select "Use demo data" from the "Data Selection" dropdown.
- Click the **"Submit"** button (if already loaded, it might not be strictly necessary, but it ensures data is properly loaded).

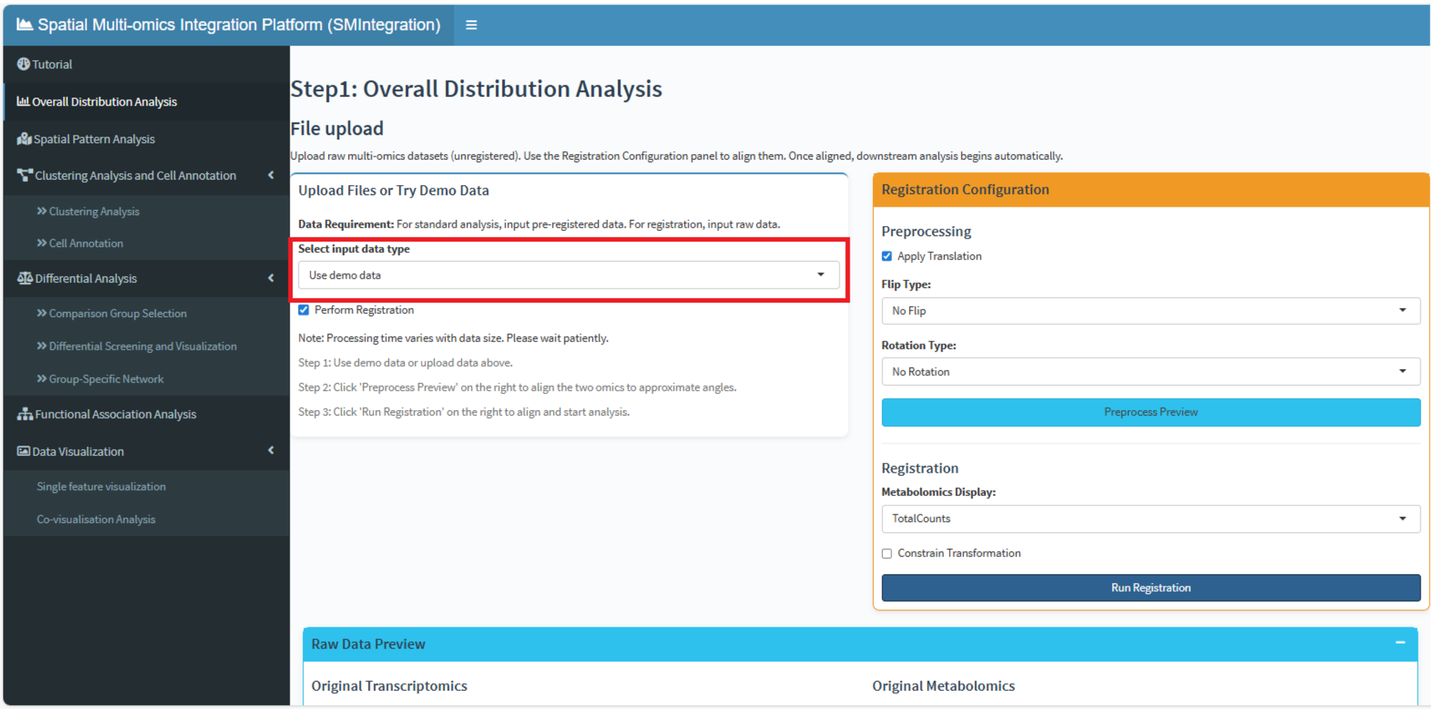

Screenshot: "Upload" tab showing file input fields with uploaded files, or "Use demo data" selected.

Step 4: Raw Data Preview and Manual Adjustments

After your data is loaded (either uploaded files or demo data), the "Raw Data Preview" panels will become active. These panels display your original metabolomics and transcriptomics spatial distributions.

Review Raw Data Preview:

- Observe the "Original Transcriptomics" and "Original Metabolomics" plots.
- For metabolomics, you can switch between "TIC" (Total Ion Count) or select a "specific m/z channel" for visualization.

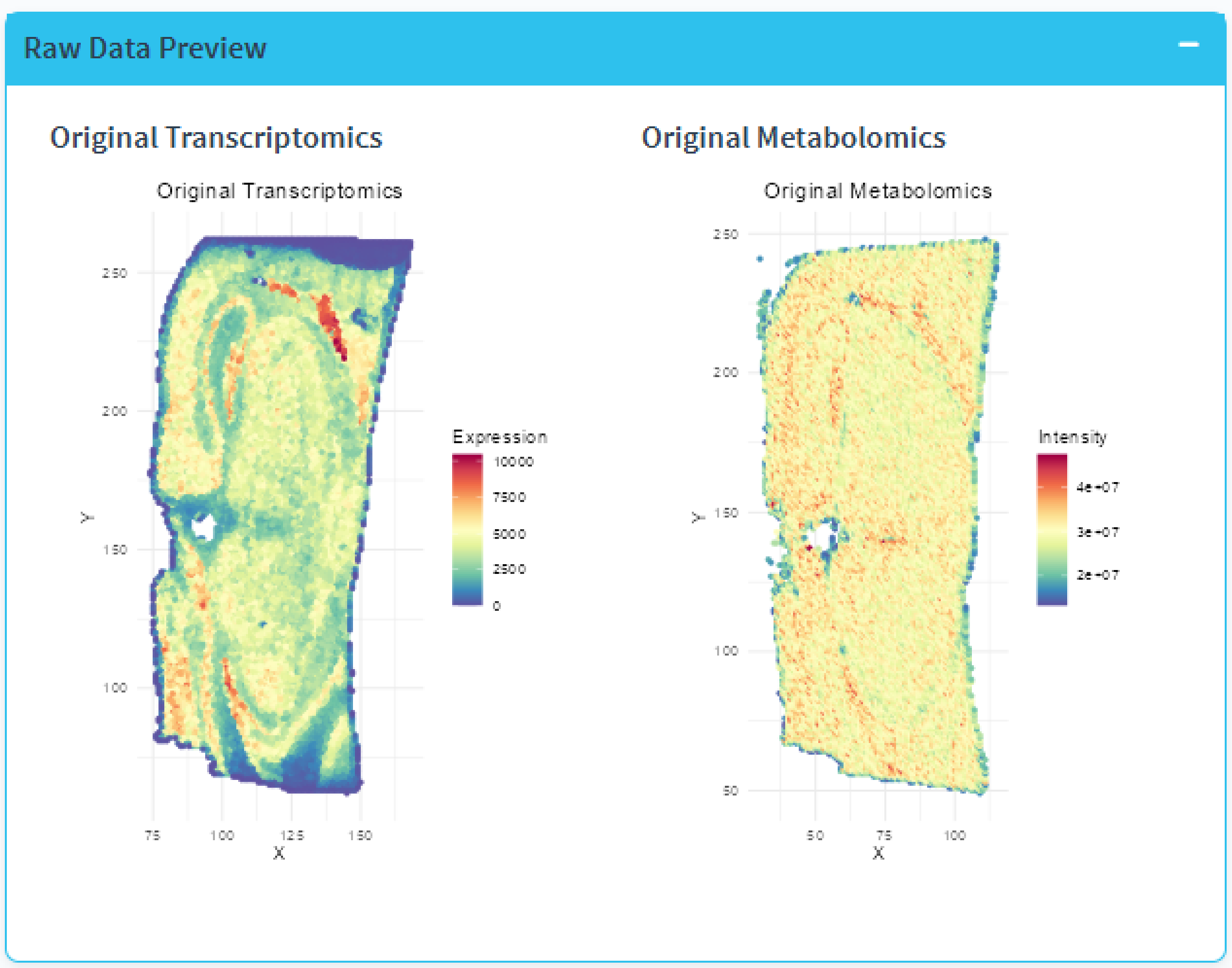

Screenshot: "Raw Data Preview" showing original metabolomics and transcriptomics plots.

Apply Manual Adjustments:

- If your data modalities are misaligned or oriented incorrectly, use the manual adjustment options:
  - **Flip Type:** Choose "vertical" or "horizontal" to flip the metabolomics data.
  - **Rotate Type:** Select "90 Degree CCW", "180 Degree CCW", or "270 Degree CCW" to rotate the metabolomics data counterclockwise.
  - **Translate (checkbox):** Check this to automatically translate the metabolomics data to align its minimum X/Y coordinates with the transcriptomics data.
- If the images appear visually aligned already, you can proceed with the default parameters and click the **"Preview Preprocessing"** button.
- After making adjustments, click the **"Preview Preprocessing"** button to see the effect on the "Preprocessed Metabolomics" plot.

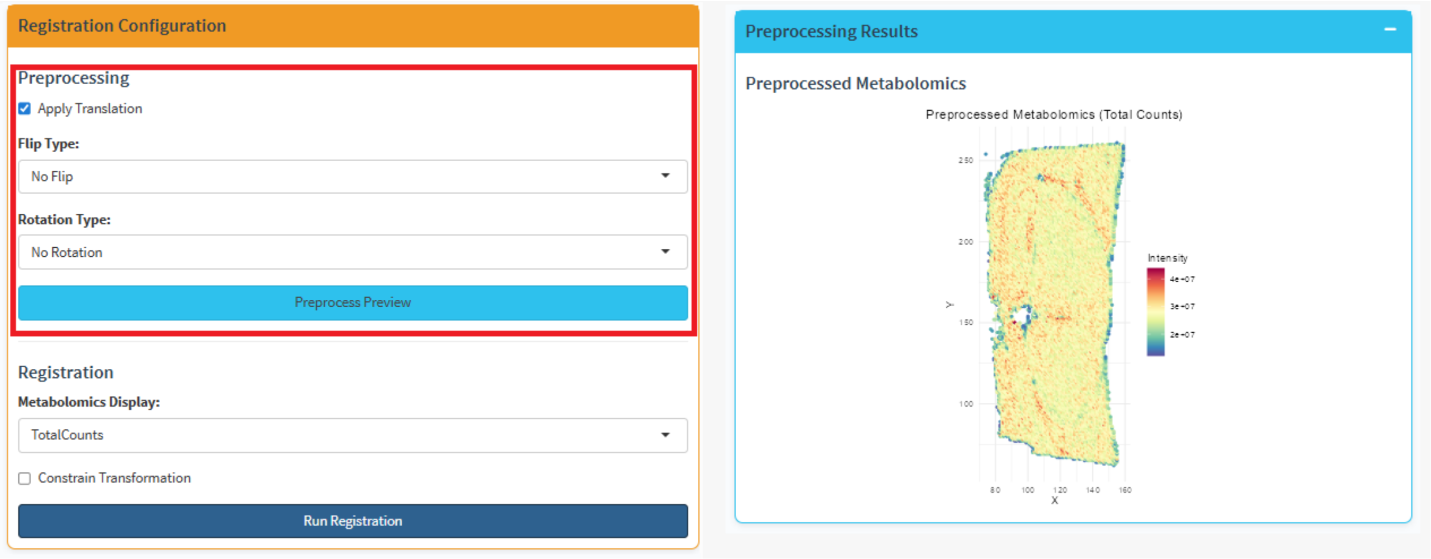

Screenshot: Manual adjustment controls (Flip, Rotate, Translate) and the "Preprocessed Metabolomics" plot.

Step 5: Run Automatic Registration

Once you are satisfied with the manual alignment, proceed to automatic registration:

Click the **"Run Registration"** button.

- The system will perform an intensity-based alignment using RNiftyReg. A progress bar will indicate the calculation status.
- For the first attempt, it is recommended to run the registration with the default settings.

Adjust **"Registration Parameters"** (if needed):

- **Constrain Transform (checkbox):** Enables a more robust transformation that checks for large rotations.
- **Max Rotation (degrees):** Adjust if your modalities have large rotational differences (e.g., >10 degrees).

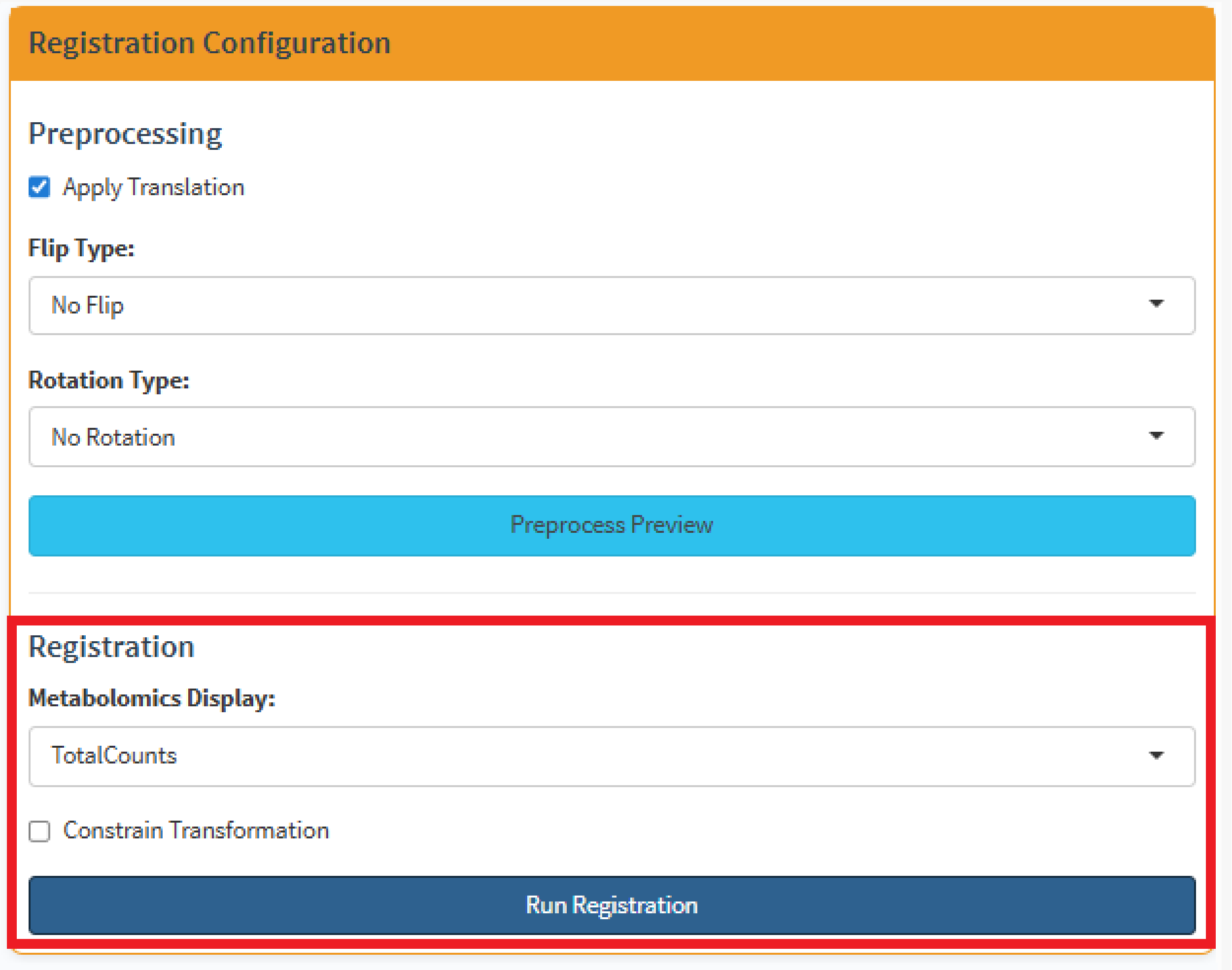

Screenshot: "Run Registration" button and associated parameters.

Step 6: Review Registration Results

After the registration process is complete, new plots and information will appear in the "Registration Results" section:

- **Overlay Plot:** An RGB overlay plot showing the aligned metabolomics (blue channel) on top of the transcriptomics data (red channel). This visualizes the quality of the alignment.

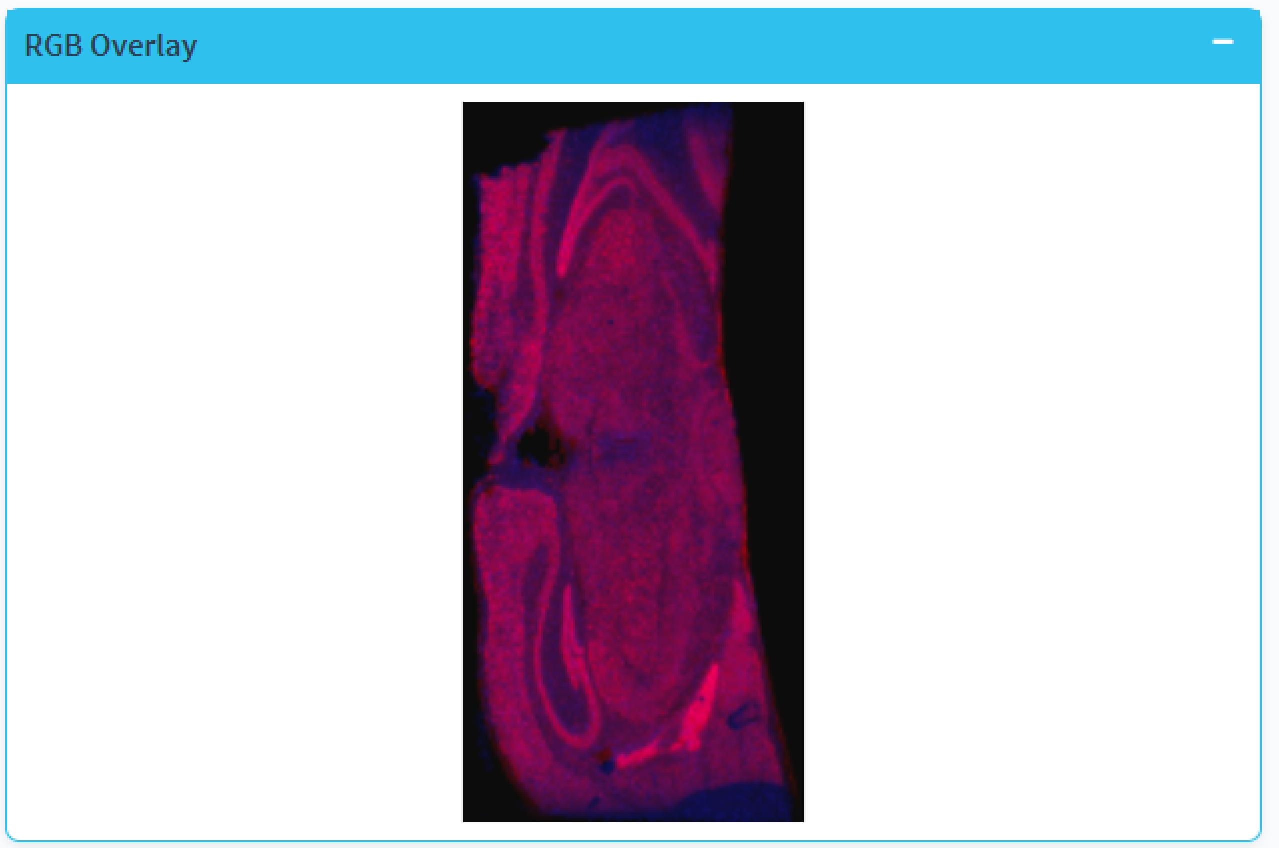

Screenshot: The RGB overlay plot.

Aligned Metabolomics Plot: The spatially registered metabolomics data. You can switch between "TIC" or a "specific m/z channel" to view its aligned distribution.

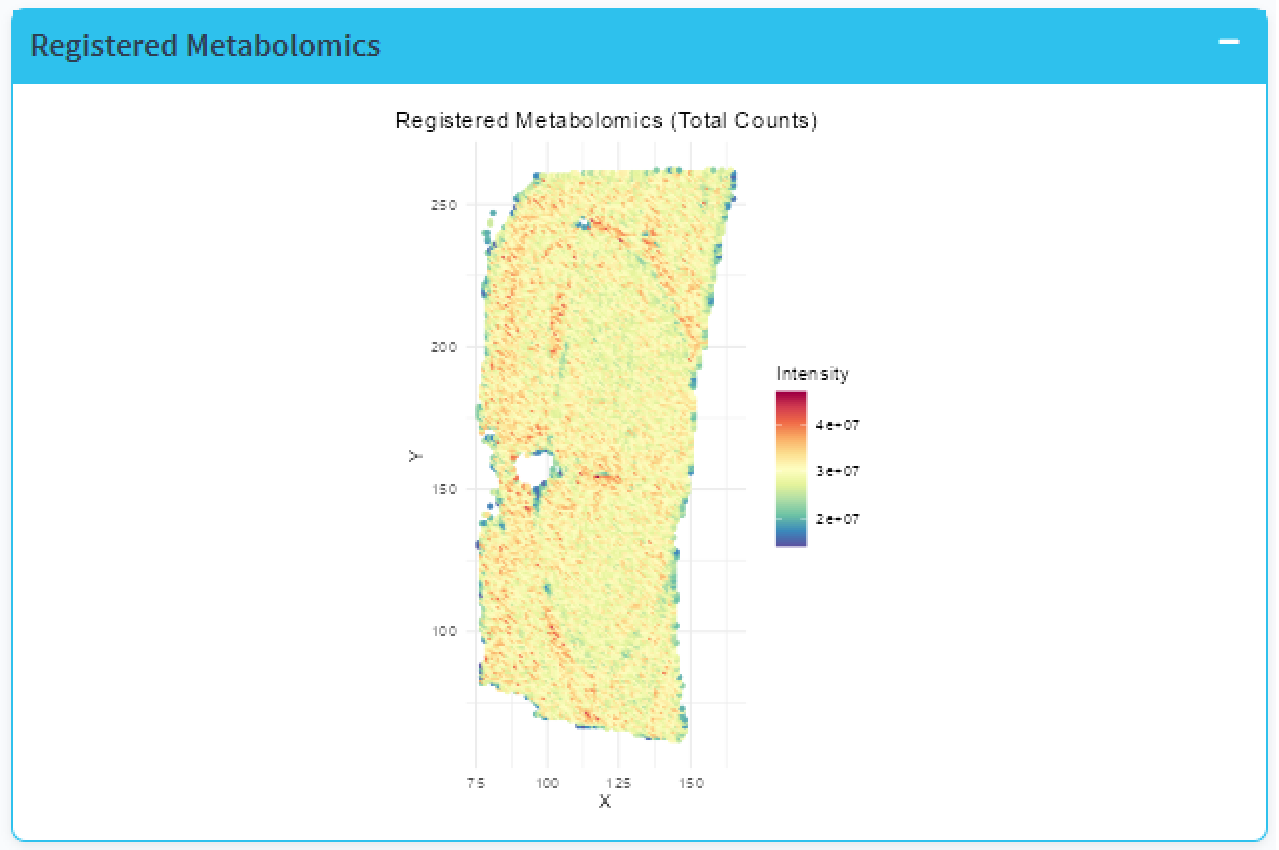

Screenshot: The "Aligned Metabolomics" plot.

Registration Quality Metrics: The "Quality Metrics" panel will display quantitative metrics such as Correlation and MSE (Mean Squared Error) to assess the alignment quality.

Quality Metrics

```
=== Data Information ===
Transcriptomics Spots: 14530
Metabolomics Spots: 13706
Preprocessing: Done
Registration: Done
Correlation: 0.784
MSE: 0.03015
Aligned Spots (Raw): 13639
Intersected Spots: 13605
```

Download All Registration Plots (ZIP)

Screenshot: "Quality Metrics" panel showing registration quality metrics.

Comparison Plot: A 2x2 grid showing the original and registered data for both modalities, allowing for a comprehensive visual comparison.

Comparison Matrix

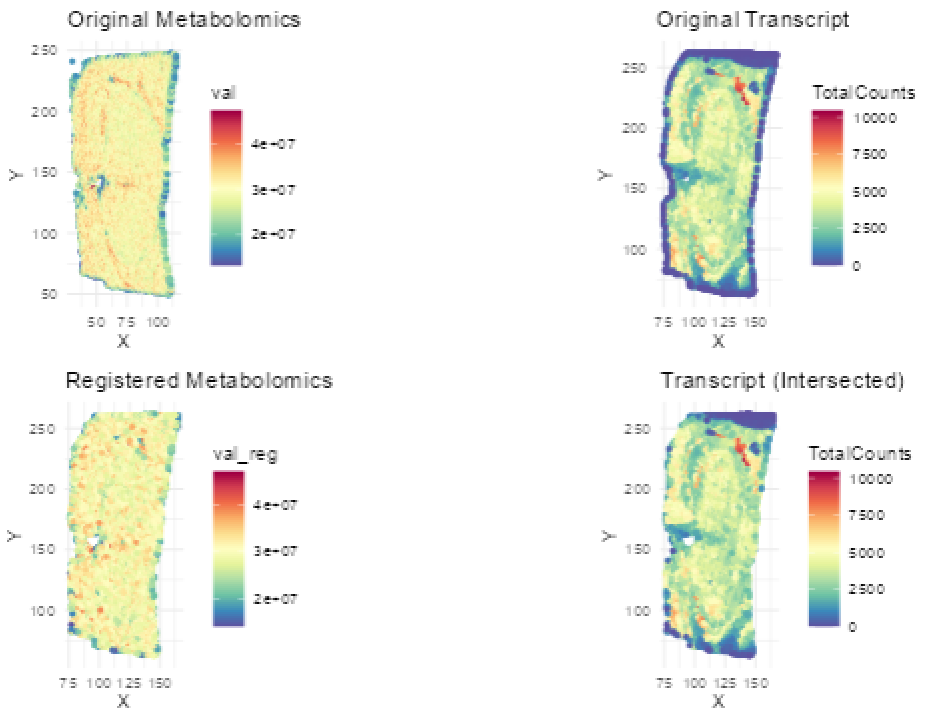

Screenshot: The 2x2 comparison plot.

You can download all registration-related plots using the provided download buttons.

Next Steps

Once registration is complete, the `data_rds` object (containing your aligned metabolomics and transcriptomics data) will be ready for downstream analysis in subsequent modules of SMIntegration.
