# Supplementary material for: SMIntegration: A web tool for comprehensive spatial metabolomics and transcriptomics integrated analysis and visualization
Source: Gigascience. 2026 Mar 24;15:giag033. doi: 10.1093/gigascience/giag033 (PMC13159472; doi:10.1093/gigascience/giag033)
Supplement: giag033_Supplemental_Files [file giag033_supplemental_files.zip › Supplementary_File_2.pdf]

Parameter Optimization Guide: Using PCA to Select Clustering Parameters

This guide demonstrates how to utilize the Principal Component Analysis (PCA) tools within **SMIntegration** to make informed, data-driven decisions for clustering parameters (e.g., the number of clusters *K* or resolution). We use the mouse brain dataset as an example to illustrate this workflow.

Step 1: Evaluating Data Dimensionality with PCA Elbow Plots

The first step in clustering optimization is determining the appropriate number of Principal Components (PCs) to use. This decision is crucial because it defines the feature space in which clustering algorithms operate.

SMIntegration generates a PCA Elbow Plot for each modality (Transcriptomics, Metabolomics, and Merged). The "Elbow Plot" displays the standard deviation (or variance explained) of each PC.

Interpretation Guide:

1. **Identify the "Knee":** Look for the point where the curve bends and begins to level off. For complex tissues, this point may be less distinct.
2. **Signal vs. Noise:** Early PCs capture major structural variations. Later PCs (before the plateau) often encode fine-grained biological heterogeneity.
3. **Selection:** Select a number of PCs that includes these subtle variations but stops before the curve completely flattens (noise).

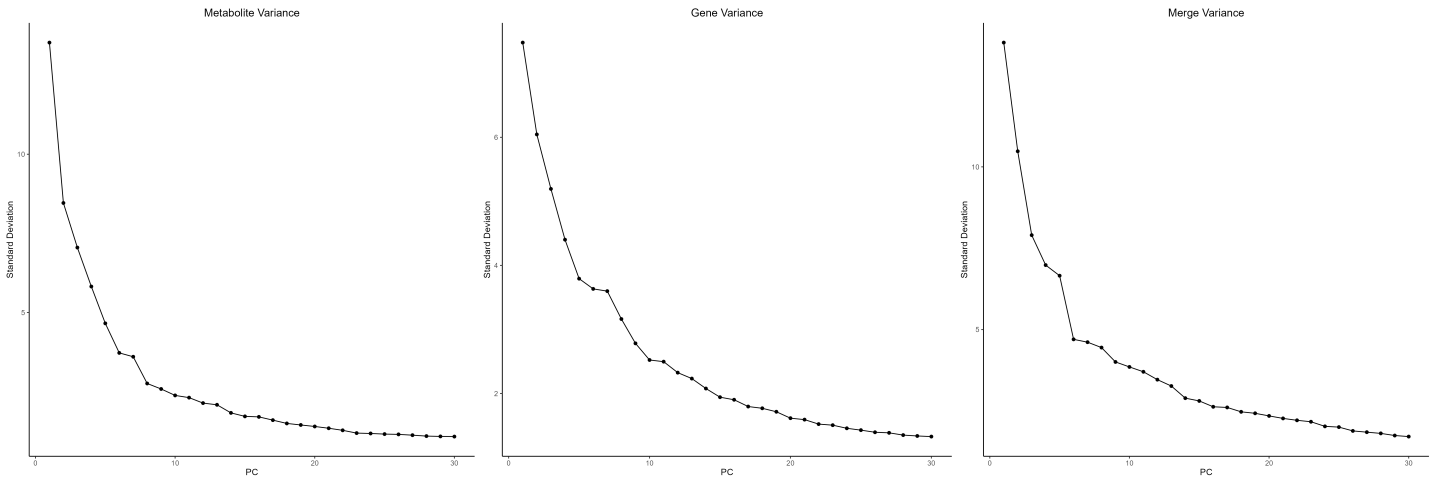

Figure 1: PCA Elbow Plots showing the standard deviation of the top 30 Principal Components. While the initial PCs capture gross variance, the curve begins to flatten around PC 20-25. We selected this range to ensure that subtle but biologically relevant spatial heterogeneity is retained for downstream clustering.

Step 2: Determining Clustering Parameters

Based on the complexity revealed by PCA, the strategy for setting parameters depends on the chosen algorithm:

Option A: K-means Methods (PCA-Kmeans, UMAP-Kmeans)

For these algorithms, you directly specify the number of clusters (*K*).

> **Strategy:** Start with a *K* value that matches the number of distinct anatomical regions you visually identified in the Spatial PCA patterns.
>
> **Example:** If PCA reveals ~20 distinct spatial gradients, test *K* values around 20-25.

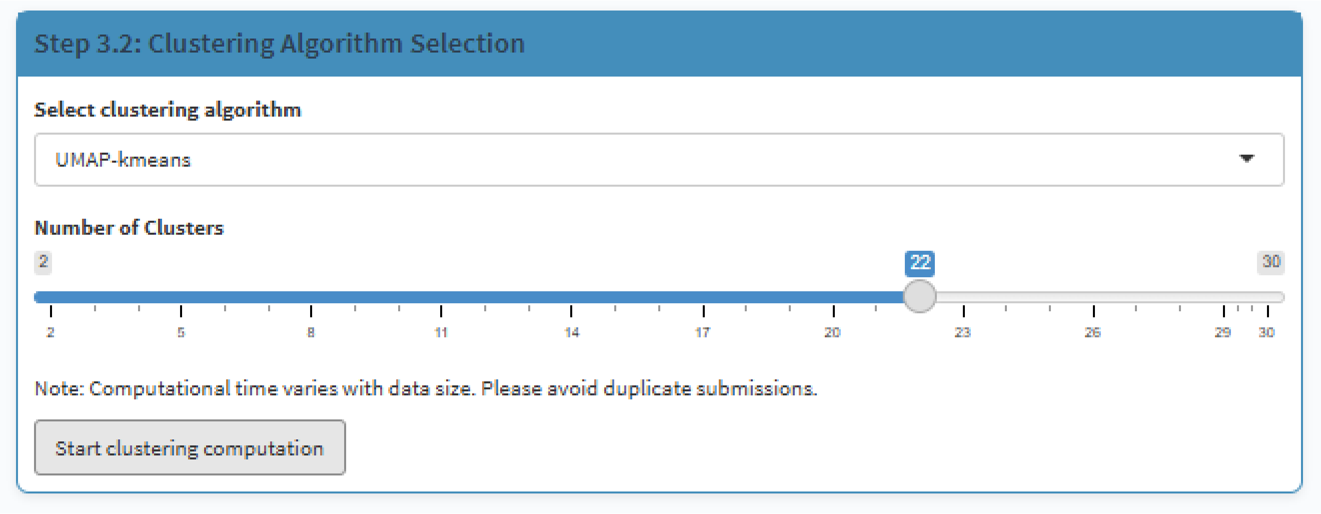

Figure 2: Impact of varying K in PCA-Kmeans. Increasing K from 5 to 25 reveals finer sub-structures.

Option B: Graph-based Methods (Louvain, SLM, LM)

For these algorithms, you adjust the **Resolution** parameter.

> **Strategy:** Higher resolution yields more clusters.
>
> **Guidance:**
>
> > **0.1 - 0.5:** For detecting broad tissue domains.
> >
> > **0.6 - 1.2:** For resolving fine sub-structures.
> >
> > **> 1.2:** For capturing very subtle heterogeneity (e.g., layers).
>
> Use the PCA complexity as a gauge: highly complex data supports higher resolutions.

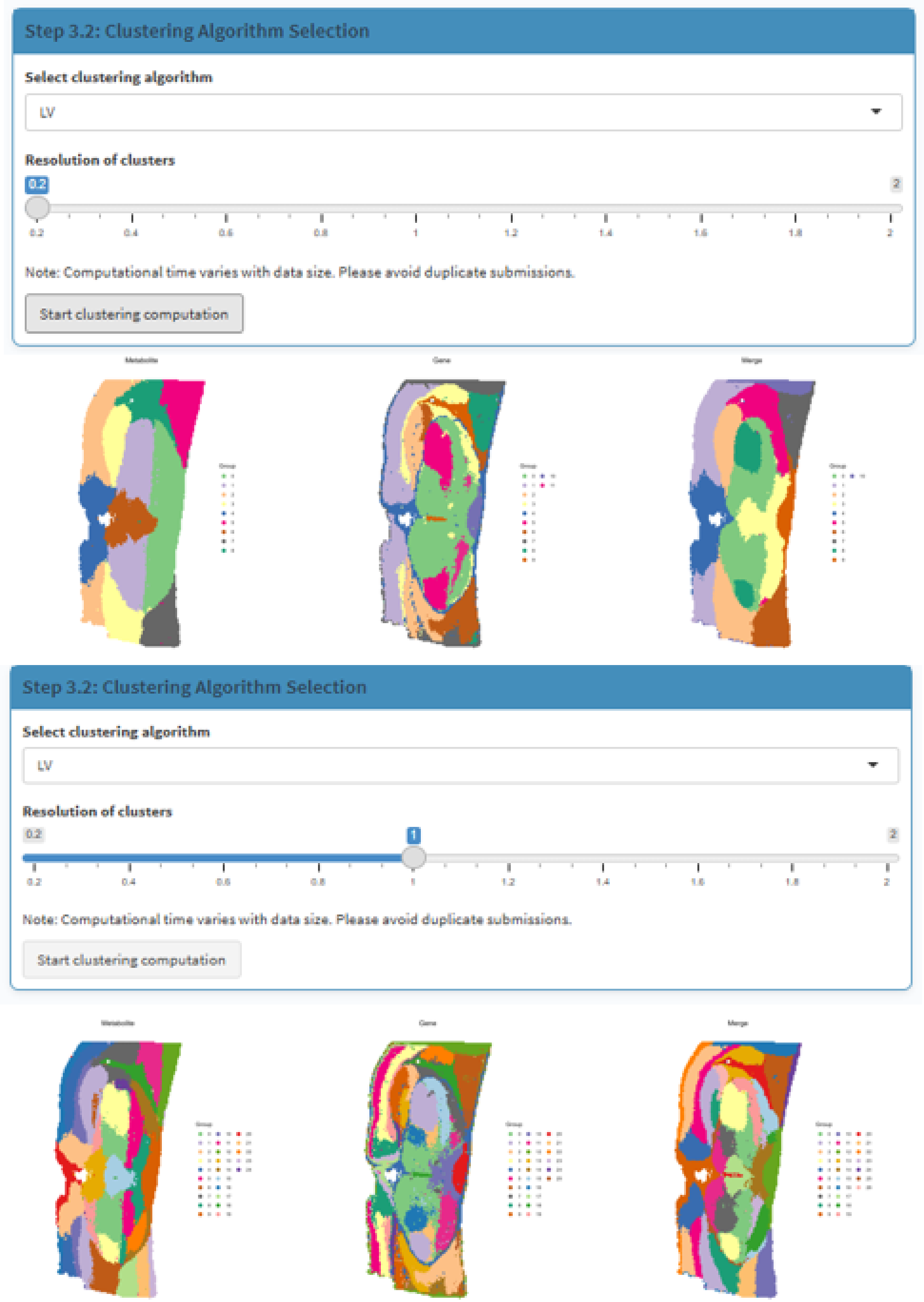

Figure 3: Impact of varying Resolution in Louvain clustering. Higher resolution (e.g., 1.0) captures more detailed spatial domains compared to low resolution (0.2).

Step 3: Final Clustering Results

After optimizing the parameters (e.g., setting *Resolution=1.0* for Louvain based on the assessment), we performed the final spatial clustering.

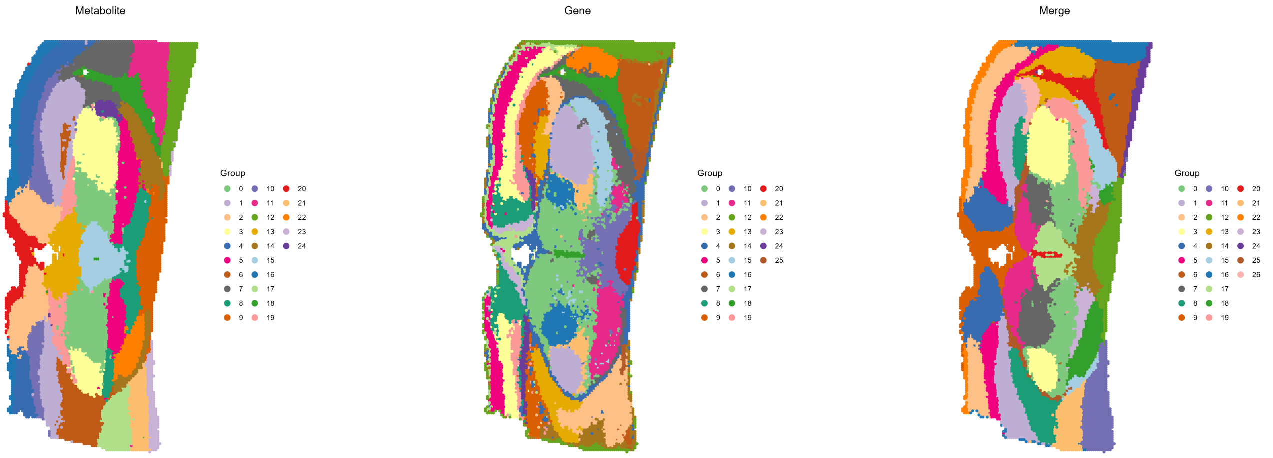

Figure 4: Final spatial clustering results for the mouse brain dataset. The integrated analysis successfully delineates fine anatomical structures.
