# Supplementary figures and images for: SMIntegration: A web tool for comprehensive spatial metabolomics and transcriptomics integrated analysis and visualization
Source: Gigascience. 2026 Mar 24;15:giag033. doi: 10.1093/gigascience/giag033 (PMC13159472; doi:10.1093/gigascience/giag033)

**A**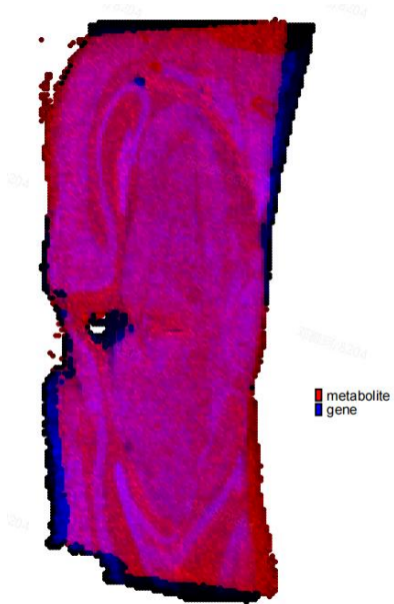**B**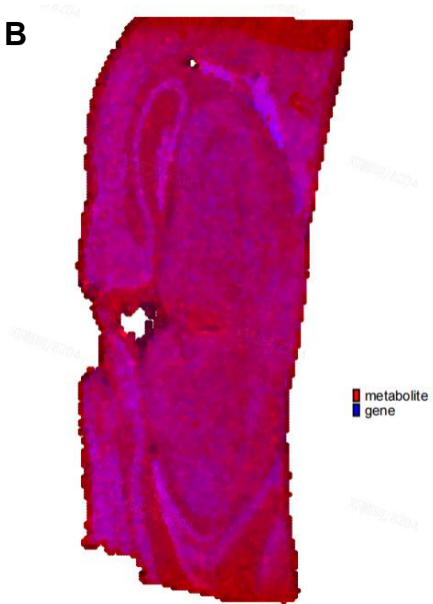

Supplement: giag033_Supplemental_Files [file giag033_supplemental_files.zip › Figure_S13.pdf]

Pathway

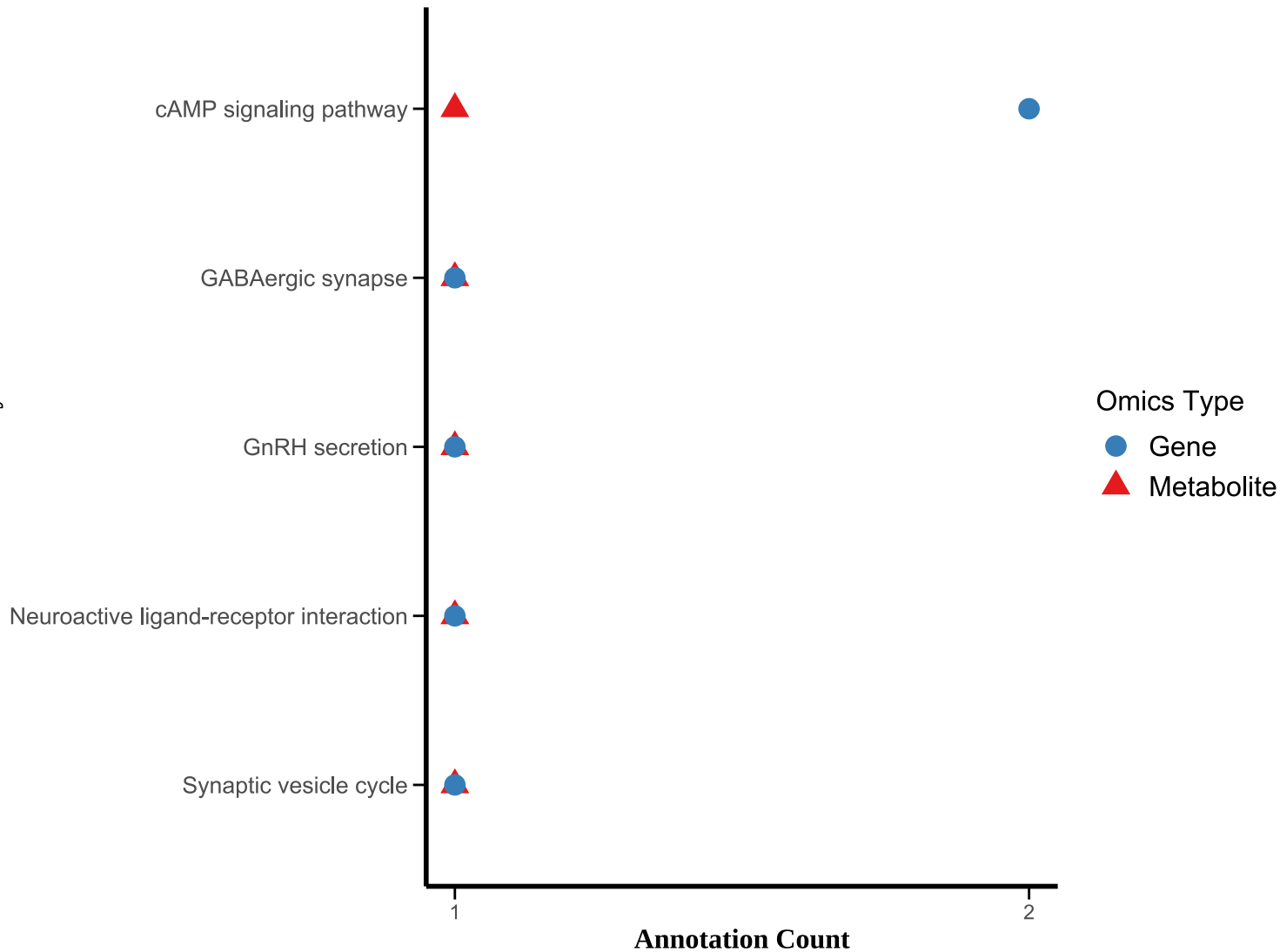

Supplement: giag033_Supplemental_Files [file giag033_supplemental_files.zip › Figure_S14.pdf]

**A**

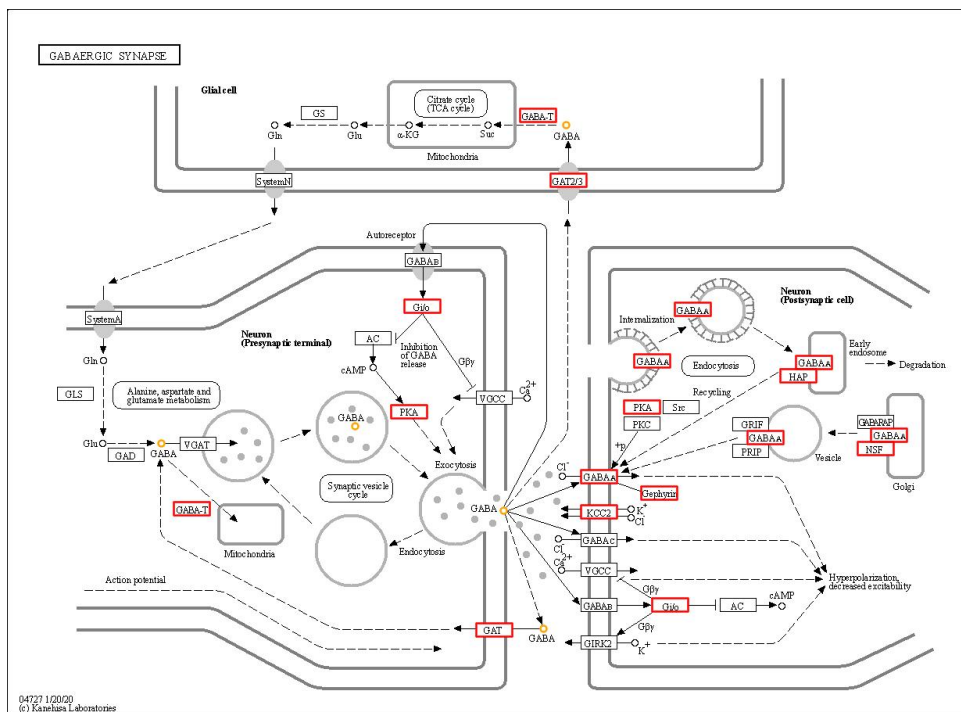

**B**

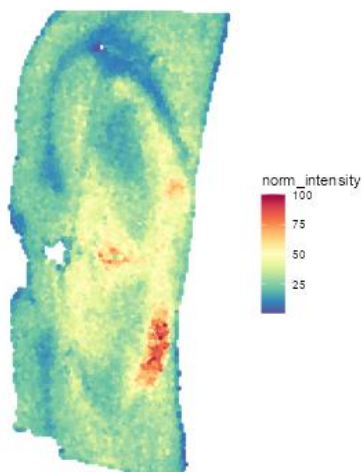

**C**

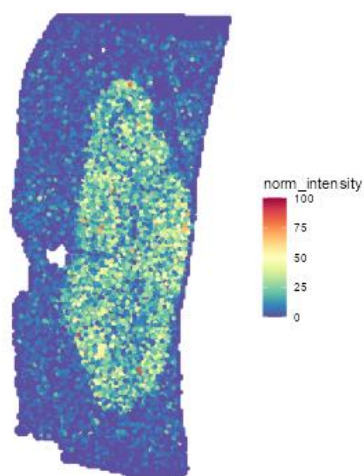

Supplement: giag033_Supplemental_Files [file giag033_supplemental_files.zip › Figure_S15.pdf]

# B

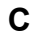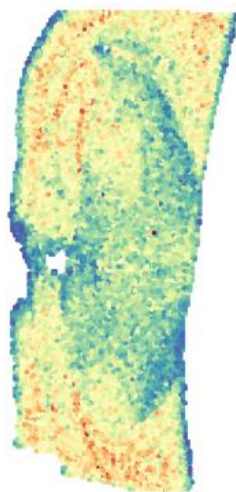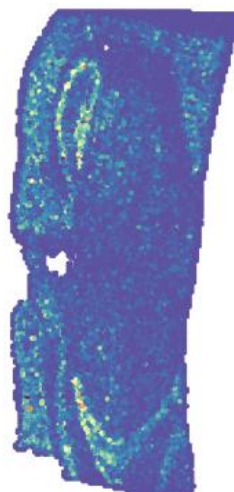

Supplement: giag033_Supplemental_Files [file giag033_supplemental_files.zip › Figure_S17.pdf]

A

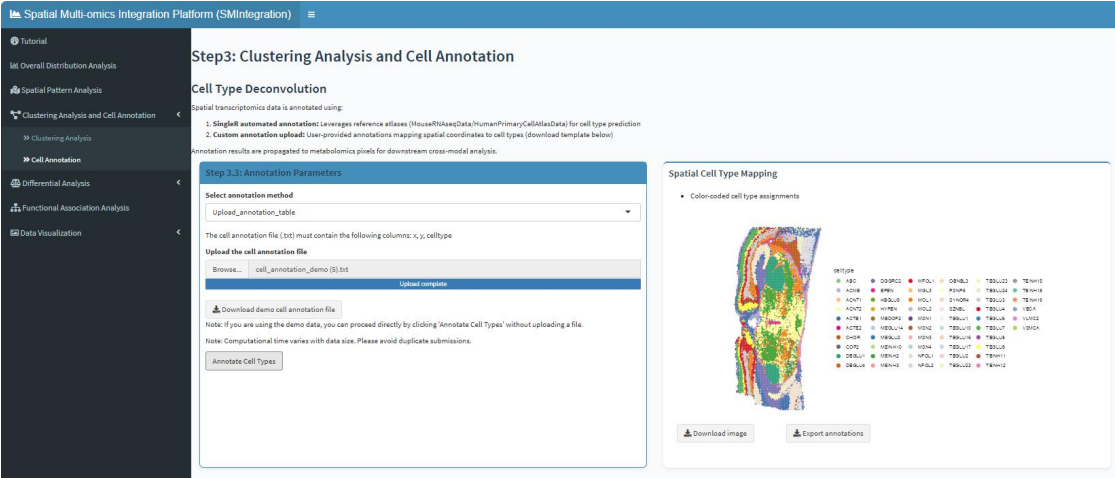

B

| x   | y   | celltype |
|-----|-----|----------|
| 74  | 177 | TEGLU24  |
| 74  | 178 | ACTE1    |
| 75  | 120 | ACTE1    |
| 75  | 121 | ACTE1    |
| ... | ... | ...      |
| 168 | 261 | ACTE1    |

Supplement: giag033_Supplemental_Files [file giag033_supplemental_files.zip › Figure_S6.pdf]

A

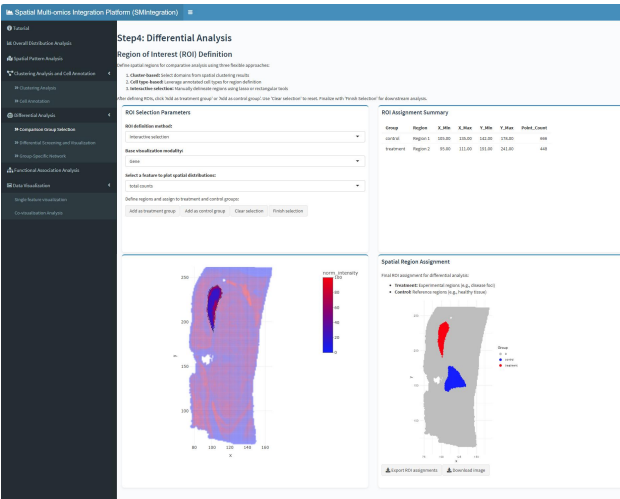

B

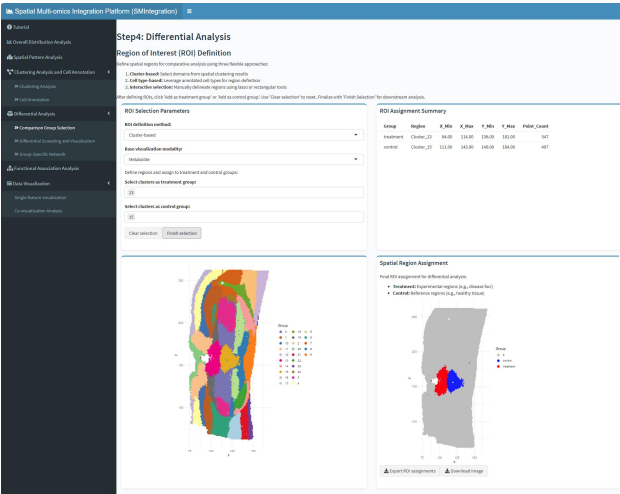

C

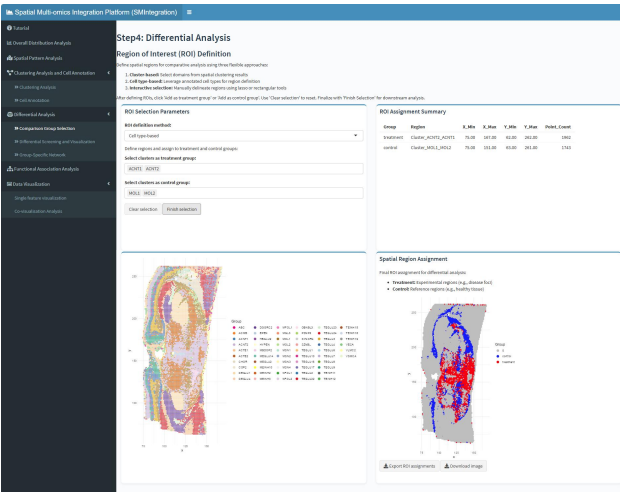

Supplement: giag033_Supplemental_Files [file giag033_supplemental_files.zip › Figure_S7.pdf]
