# Supplementary material for: SMIntegration: A web tool for comprehensive spatial metabolomics and transcriptomics integrated analysis and visualization
Source: Gigascience. 2026 Mar 24;15:giag033. doi: 10.1093/gigascience/giag033 (PMC13159472; doi:10.1093/gigascience/giag033)
Supplement: giag033_GIGA-D-25-00440_original_submission [file giag033_giga-d-25-00440_original_submission.pdf]

GigaScience

SMIntegration: A Web Tool for Comprehensive Spatial Metabolomics and Transcriptomics Integrated Analysis and Visualization

--Manuscript Draft--

**Manuscript Number:**	GIGA-D-25-00440
**Full Title:**	SMIntegration: A Web Tool for Comprehensive Spatial Metabolomics and Transcriptomics Integrated Analysis and Visualization
**Article Type:**	Technical Note
**Funding Information:**	the National Key R&D Program of China (2021YFA0805100) — Not applicable<br>Major Scientific and Technological Special Project of Guizhou Province (KCXFZ20240903093925033) — Not applicable
**Abstract:**	Current tools for spatial omics analysis often face challenges in performing integrated transcriptomics and metabolomics analysis, in-depth biological interpretation, and user-friendly operation. To address this, we developed SMIntegration, the first web-based graphical platform designed specifically for integrated spatial metabolomics and transcriptomics analysis. Built with R/Shiny and deployed using Docker containerization, the platform provides a complete analytical workflow from data preprocessing to functional annotation. Its core functions include: (i) cross-modal spatial pattern recognition; (ii) flexible differential analysis of genes and metabolites based on clustering results, user-defined regions, or cell type annotations; and (iii) group-specific gene-metabolite network construction and interactive visualization. Using adjacent mouse brain coronal sections (Stereo-seq transcriptomics and AFADESI-MS metabolomics) as an example, SMIntegration successfully identified both the periaqueductal gray and subcommissural organ, which were missed by single-modality clustering. Cell type analysis revealed an association between astrocyte-enriched GABA metabolism and Slc6a11, while a comparison between the cornu ammonis region and the midbrain periaqueductal gray dissected glutamatergic and endogenous cannabinoid signaling pathway modules. With a zero-code interface, SMIntegration enables a wide range of researchers to deeply explore gene-metabolite interaction mechanisms within microenvironments during development, homeostasis, and disease.
**Corresponding Author:**	Zhanlong Mei, Ph.D.<br>BGI-Shenzhen: BGI Group<br>Shenzhen, Guangdong CHINA
**Corresponding Author Secondary Information:**	
**Corresponding Author's Institution:**	BGI-Shenzhen: BGI Group
**Corresponding Author's Secondary Institution:**	
**First Author:**	Haoke Deng
**First Author Secondary Information:**	
**Order of Authors:**	Haoke Deng
	Xiaolian Ning
	Xun Lin
	Liang Zong
	Shanqiao Zheng
	Yun Zhao
	Jing Wang

Powered by Editorial Manager® and ProduXion Manager® from Aries Systems Corporation

	Lingyun Chen
	Jin Zi
	Zhanlong Mei
**Order of Authors Secondary Information:**	
**Additional Information:**	
**Question**	**Response**
Are you submitting this manuscript to a special series or article collection?	No
**Experimental design and statistics**<br><br>Full details of the experimental design and statistical methods used should be given in the Methods section, as detailed in our Minimum Standards Reporting Checklist. Information essential to interpreting the data presented should be made available in the figure legends.<br><br>Have you included all the information requested in your manuscript?	Yes
**Resources**<br><br>A description of all resources used, including antibodies, cell lines, animals and software tools, with enough information to allow them to be uniquely identified, should be included in the Methods section. Authors are strongly encouraged to cite Research Resource Identifiers (RRIDs) for antibodies, model organisms and tools, where possible.<br><br>Have you included the information requested as detailed in our Minimum Standards Reporting Checklist?	Yes
**Availability of data and materials**<br><br>All datasets and code on which the conclusions of the paper rely must be either included in your submission or deposited in publicly available repositories (where available and ethically	Yes

Powered by Editorial Manager® and ProduXion Manager® from Aries Systems Corporation

appropriate), referencing such data using a unique identifier in the references and in the “Availability of Data and Materials” section of your manuscript.<br><br>Have you have met the above requirement as detailed in our Minimum Standards Reporting Checklist?	
GigaScience has policies and guidelines in place for the use of generative AI-writing tools such as ChatGPT. If you have used such writing tools to assist with writing the manuscript this must be declared and cited in the text. Authors should not list AI-writing tools and other AI-assisted technologies as an author or co-author and should acknowledge that they are fully responsible for text generated or refined by AI-writing tools.<p><br><br>A summary of use (particularly in the introduction or among methods) needs to be included at the end of the paper, and the outputs should also be included as a supplementary file hosted in GigaDB or other open repositories. Please read our guidelines for more information. <p><br><br>By submitting to GigaScience, you are aware of the journal's AI-writing tools policy, and if you have declared use of such tools below, you have acknowledged this where appropriate in your manuscript and have made a summary of use and outputs available. <p><br><b>AI-assisted writing tools have been used in the preparation of this manuscript?	Yes

Powered by Editorial Manager® and ProduXion Manager® from Aries Systems Corporation

manuscript

Click here to access/download;Manuscript;gpb-manuscript.docx

Title page

Article Title

SMIntegration: A Web Tool for Comprehensive Spatial Metabolomics and Transcriptomics Integrated Analysis and Visualization

Author(s) Names

Haoke Deng[1#], Xiaolian Ning[2#], Xun Lin[1], Liang Zong[1], Shanqiao Zheng[1], Yun Zhao[1], Jing Wang[1], Lingyun Chen[2], Jin Zi[1*], Zhanlong Mei[1*]

Author(s) Address Information

[1] *BGI, Shenzhen 518083, China*

[2] *BGI Research, Shenzhen 518083, China*

Symbol

[#] Equal contribution.

[*] Corresponding author(s).

E-mail: denghaoke@genomics.cn (Deng H), ningxiaolian@bgitechsolutions.com(Ning X) , linxun@genomics.cn (Lin X), zongliang@genomics.cn(Liang Z), zhengshanqiao@genomics.cn (Zheng S), zhaoyun@genomics.cn (Zhao Y), wangjing@genomics.cn(Wang J), chenlingyun@genomics.cn (Chen L), zij@genomics.cn (Zi J), meizhanlong@genomics.cn (Mei Z)

Running title: *Deng H et al / SMIntegration for Spatial Multi-omics*

[a]ORCID: 0000-0002-1381-4818.

[b]ORCID: 0009-0002-1213-4754.

[c]ORCID: 0009-0006-0169-6005.

[d]ORCID: 0000-0003-3751-4198.

[e]ORCID: 0009-0008-1685-8059.
[f]ORCID: 0000-0001-7363-9487.
[g]ORCID: 0009-0009-8545-4573.
[h]ORCID: 0000-0001-5869-842X.
[i]ORCID: 0000-0002-1891-1393.
[j]ORCID: 0000-0003-2203-2495.

Total word counts (from “Introduction” to “Conclusions” or “Materials and methods”): 2664

Total figures: 4

Total tables: 0

Total supplementary figures: 17

Total supplementary tables: 1

Total supplementary files: 20

Abstract page

Abstract

Current tools for spatial omics analysis often face challenges in performing integrated transcriptomics and metabolomics analysis, in-depth biological interpretation, and user-friendly operation. To address this, we developed SMIntegration, the first web-based graphical platform designed specifically for integrated spatial metabolomics and transcriptomics analysis. Built with R/Shiny and deployed using Docker containerization, the platform provides a complete analytical workflow from data preprocessing to functional annotation. Its core functions include: (i) cross-modal spatial pattern recognition; (ii) flexible differential analysis of genes and metabolites based on clustering results, user-defined regions, or cell type annotations; and (iii) group-specific gene-metabolite network construction and interactive visualization. Using adjacent mouse brain coronal sections (Stereo-seq transcriptomics and AFADESI-MS metabolomics) as an example, SMIntegration successfully identified both the periaqueductal gray and subcommissural organ, which were missed by single-modality clustering. Cell type analysis revealed an association between astrocyte-enriched GABA metabolism and *Slc6a11*, while a comparison between the cornu ammonis region and the midbrain periaqueductal gray dissected glutamatergic and endogenous cannabinoid signaling pathway modules. With a zero-code interface, SMIntegration enables a wide range of researchers to deeply explore gene-metabolite interaction mechanisms within microenvironments during development, homeostasis, and disease.

KEYWORDS: Spatial multi-omics; Spatial pattern analysis; Spatial differential analysis; Gene-metabolite co-localization; Differential expression network

Text body page

Introduction

Spatial omics technologies have revolutionized molecular biology by enabling localization of molecular information within tissue sections [1]. Spatial multi-omics, integrating transcriptomics, proteomics, and metabolomics, was recognized by Nature in 2022 as a "Technology to Watch" [2]. Integrating spatial transcriptomics and metabolomics is particularly important as it links gene expression (genotype) with metabolic products (phenotype), revealing mechanisms in development, disease, and therapy. Recent studies highlight this power: Sun et al. [3] profiled gastric cancer metabolic remodeling, while Vicari et al. [4] developed a protocol for simultaneous profiling on a single slice. Such approaches enable spatial clustering comparisons [3,5], identification of synergistic gene–metabolite modules [4,6], and cross-region interaction analyses [3,6]. These insights clarify how spatially defined genes regulate the metabolic microenvironment, advancing understanding of tissue development and disease.

Despite progress, current computational tools face three main limitations. First, modality compatibility is limited: platforms like SpatialGlue [7] and Giotto [8] mainly address transcriptomics–proteomics, and SpaTrio [9] links single-cell multi-omics with spatial transcriptomics. No standardized pipeline exists for transcriptomics–metabolomics integration. Second, biological interpretation remains shallow. SODB [10] allows data loading but not deep analysis; MIIT [11] supports registration but not interaction studies. Third, accessibility is poor: tools like MISO [12] and SOAPy [13] require programming expertise, while recent machine learning methods [14] are complex and lack GUIs. These gaps hinder research on how gene regulation shapes spatial metabolism, emphasizing the need for multimodal, analytical, and user-friendly platforms.

To address this, we developed SMIntegration, the first GUI platform for joint spatial metabolomics–transcriptomics analysis. It provides a complete pipeline from preprocessing to functional annotation, lowering technical barriers. Core functions

include: (1) Spatial pattern analysis to identify co-varying features across omics; (2) Differential analysis based on clustering, user-defined ROIs, or cell types, with integrated functional enrichment; and (3) Network analysis and visualization to construct differential expression genes (DEG)/ differential abundant metabolites (DAM) correlation networks and explore spatial co-localization. Validated on mouse brain data, SMIntegration integrates both modalities, identifies fine brain structures, reveals astrocyte and oligodendrocyte networks, and uncovers mechanisms of synaptic plasticity and pain regulation, demonstrating strong potential for systems-level studies.

Methods

Software Implementation and Architecture

SMIntegration is a web-based GUI implemented in R (v4.4.2) using Shinyproxy and Docker, ensuring scalability and portability. Users access it via browser. The cloud platform (128 CPUs, 1000 GB RAM) is available at https://metax.genomics.cn/app/smintegration. Runtime benchmarks are in Table S1. For very large datasets, local deployment is recommended. Source code is on GitHub (https://github.com/mzlab-research/SMIntegration.git), with documentation, tutorials, and example datasets accessible from the help interface (Figure S1).

Data Preparation and Spatial Registration

Input requires spatially registered metabolomics and transcriptomics data. Since resolutions differ, higher-resolution data should be aggregated to match the lower (e.g., binning 500 nm transcriptomics by 100 to 50 μm metabolomics). Registration can be performed using SpatialData [15], which supports linear and affine transformations. A tutorial and scripts are provided in Supplementary File 1 and GitHub (https://github.com/mzlab-research/SMIntegration/tree/main/spatialdata). Two input formats are supported: (1) text matrices containing feature name, spatial x/y coordinates, and values (Figure S2A); (2) Seurat objects with coordinates and abundance in designated slots (Figure S2B). Upload requirements are detailed on the help page.

Data Upload and Visualization

On the Overall Distribution Panel, users can upload datasets or use built-in test data (Figure S3). The system performs format checks, retains overlapping pixels, and generates abundance maps for both omics (**Figure 1A**).

Core Analysis Modules

Spatial Expression Pattern Recognition

This module applies SpaGene [16] to detect spatially variable features. High-expression subnetworks are quantified using Earth mover's distance (EMDg) and compared with random permutations. Non-negative matrix factorization (NMF) partitions features into modules (**Figure 1B**). Cross-omics associations are evaluated using Moran's I [17]. Users can browse features within modules (Figure S4).

Pixel-level Spatial Clustering

Four clustering methods are available (**Figure 1C**): Louvain (LV), LM, SLM [18], and K-means after UMAP [19,20]. Preprocessing includes normalization (LogNormalize), variable feature selection (top 2000 genes/metabolites), and scaling. Integrated data combines both modalities by pixel coordinates. A Sankey diagram compares clustering concordance (Figure S5).

Cell Type Annotation

Cell types are annotated for transcriptomics using SingleR [21] with reference datasets (MouseRNAseqData, HumanPrimaryCellAtlasData), then mapped to metabolomics pixels (**Figure 1D**). Users may also upload custom annotations (Figure S6).

Differential Analysis

This analysis consists of two steps: ROI selection and differential testing. ROIs can be defined in three ways (**Figure 1E**): Interactive Selection (manually drawing on metabolomics ion maps or transcriptomics expression maps, Figure S7A), Clustering-based Selection (Figure S7B), and Cell Type-based Selection (Figure S7C). After defining ROIs, users specify groups (e.g., Region A vs. Region B). Each pixel is treated as an independent sample, and Seurat's FindMarkers function [18] with a Wilcoxon rank-sum test identifies DEGs and DAMs. Results are Bonferroni-corrected [22], with default thresholds of $|log2FC| > 0.26$ and adjusted $p < 0.05$. In addition to univariate

analysis, UMAP visualization highlights expression differences across ROIs, and users can view spatial distributions of identified DEGs or DAMs (Figure S8).

Group-specific Network Construction of Differential Features

This module reveals spatial co-expression relationships between DEGs and DAMs under specific biological conditions (Figure 1E). For each comparison group, differential genes and metabolites are first selected, and Spearman correlation coefficients are calculated using pixel-level data. Pairs meeting adjusted p-value < 0.01 and $|r| > 0.6$ are retained as network edges. By enforcing the same node layout across groups (Figure S9), users can intuitively observe changes in gene–metabolite correlation patterns, facilitating understanding of group-specific regulatory mechanisms.

Functional Association and Enrichment

This module integrates and interprets biological functions of DEGs and DAMs (**Figure 1F**). It performs pathway mapping (e.g., KEGG), then applies Fisher's exact test for co-enrichment analysis to identify pathways synergistically enriched. Key pathway nodes are displayed with up/downregulation, and users can view spatial distribution images of all annotated genes and metabolites in any enriched pathway (Figure S10).

Spatial Imaging Visualization

SMIntegration provides spatial visualization tools for exploring distribution patterns (**Figure 1G**). It supports single-feature imaging to generate spatial maps for any gene or metabolite, feature co-localization to display the top six positively and negatively correlated genes and metabolites for a selected feature (Figure S11), and multi-feature visualization, where two to three features can be mapped to RGB channels to generate pseudo-color composite images (Figure S12). Together, these functions offer an intuitive means to explore spatial multi-omics relationships.

Example Data and Validation

SMIntegration includes datasets from adjacent coronal brain sections of a 7-week-old male mouse. Spatial metabolomics data were acquired by AFADESI-MS (50 μm resolution), processed with Cardinal [23] and identified with SManalyst platform [24], and yielded 13,707 pixels and 560 metabolites. Spatial transcriptomics data from

Stereo-seq were binned to 50 μm, resulting in 14,605 pixels and 10,000 highly variable genes. The two modalities were registered using SpatialData (Figure S13), followed by KNN interpolation and filtering, yielding 14,530 valid pixels. A downsampled demo dataset (500 genes and 500 metabolites) is also provided, allowing users to quickly test platform functions via the "Use demo data" option in the Overall Distribution Analysis panel.

Results and Discussion

Integrated Spatial Pattern Analysis Reveals Covarying Molecular Landscapes

SMIntegration identifies spatially consistent regions and molecular patterns by offering a variety of clustering algorithms and spatial pattern recognition methods. Joint clustering of integrated spatial metabolomics and transcriptomics data demonstrates improved spatial domain identification. We matched our mouse brain imaging data to the Allen Mouse Brain Reference Atlas [25] using DeepSlice [26] to identify the closest matching atlas plate, and then registered this atlas plate to our experimental images using QuickNII [27], and **Figure 2A** shows the brain structure of the mouse after registration. While separate spatial metabolomics clustering could only identify the periaqueductal gray (PAG) (**Figure 2B**), and separate transcriptomics clustering could only identify the subcommissural organ (SCO) (**Figure 2C**), the joint clustering of both spatial metabolomics and transcriptomics data accurately identified both regions simultaneously (**Figure 2D**). This highlights the potential of integrating different modalities to resolve fine spatial heterogeneity. The relationship between the clustering results of different modalities is visualized through a Sankey diagram (Figure S5), which demonstrates the unique information and degree of correspondence contributed by each omics layer to the spatial stratification.

In addition to spatial clustering, SMIntegration uses the SpaGene method to identify molecular expression patterns. **Figures 2E** and **2F** display the identified spatial expression patterns for genes and metabolites, while **Figure 2G** shows the correlation between these spatial patterns. Although the overall clustering distributions exhibit similarities, the specific patterns differ. Some patterns appear to be complementary

between modalities, such as metabolite pattern 2 and gene pattern 4 (r=-0.607). However, conserved cross-modal patterns were also found, such as metabolite pattern 3 and gene pattern 2 (r=0.565), both of which are enriched in the mid-brain region (Figures 2E, 2F). We performed functional enrichment analysis on the conserved pattern pair (Figure S14). The results revealed significant co-enrichment of both patterns in key pathways such as synaptic vesicle cycle and neuroactive ligand-receptor interaction. This finding indicates that the co-localized module is deeply involved in the regulation of synaptic signaling in the midbrain region. It is noteworthy that the cAMP signaling pathway was also enriched. This pathway represents a classic intracellular signaling cascade that translates neurotransmitter receptor activation into changes in neuronal excitability and gene expression and is closely associated with synaptic plasticity [28].

The above analysis demonstrates the powerful capability of the SMIntegration platform in identifying spatially co-localized multi-omic modules. By recognizing coordinated spatial patterns of genes and metabolites, it can be directly linked to functional synergy. This discovery provides a novel molecular framework for investigating region-specific mechanisms of motor control and reward, highlighting the unique value of integrated spatial multi-omics analysis.

Cell-Specific Metabolite Analysis

Deciphering gene expression and metabolite abundance changes within specific cell types is crucial for a deeper understanding of cellular function. However, spatial metabolomics data itself lacks direct cell type annotation capabilities. SMIntegration effectively addresses this challenge through its integrated cell type annotation and piexl registration (Figure 1D), which allows researchers to directly compare metabolic expression differences within regions enriched for different cell types. We demonstrate this by comparing two functionally distinct glial cell populations in the mouse brain: regions dominated by non-telencephalon astrocytes (NA) versus regions dominated by Mature oligodendrocytes (MO) (**Figure 3A**, Figure S7C). UMAP analysis showed clear differences in metabolite (**Figure 3B**) and gene (**Figure 3C**) expression between these two cell types. Through differential screening, SMIntegration identified 103 differential

metabolites, with 58 upregulated in NA and 45 upregulated in MO. In terms of genes, 1313 genes were highly expressed in the NA region, while 427 were highly expressed in the MO region (**Figure 3D**). Notably, the gene *Slc6a11* and the metabolite gamma-Aminobutyric acid (GABA) were jointly annotated to the GABAergic synapse pathway (Figure S15A). Both were upregulated in the NA region (Figure S15B, C). *Slc6a11* encodes a sodium-dependent transporter [29], and its absence can lead to GABA accumulation and an imbalance in neuronal excitability, affecting cognitive function [30]. These differential results are consistent with the cellular functions of astrocytes, which play a role in GABA synthesis and transmission.

Cell-type-specific metabolic regulatory networks provide a new perspective for dissecting the mechanisms of metabolic-gene synergistic interactions. We constructed correlation networks for these differential features in the NA and MO regions separately. In the NA region, no significant correlation was observed between *Bex2* and spermidine (Figure 3E); whereas in the MO region, decreased expression of the *Bex2* gene and upregulation of spermidine were detected, showing a negative correlation between them (Figure 3F). *Bex2* has anti-apoptotic and pro-proliferative properties [31], while the effect of spermidine on the immune system is dose-dependent, with higher doses being anti-inflammatory and lower doses enhancing cytotoxic immune function [32]. This phenomenon reveals that the correlation between the molecules *Bex2* and spermidine is highly specific to cellular function: the significant negative correlation observed in the MO region, where decreased *Bex2* expression coincides with increased spermidine levels, strongly suggests the presence of a finely tuned inhibitory regulation—mediated by spermidine accumulation—targeting cell proliferation and apoptosis. In contrast, the lack of correlation in the NA region may indicate a functional decoupling between the two. Such specificity in interaction is likely closely related to the immune microenvironment or neural activity demands of the cell population. These results reflect the heterogeneity of transcriptomics and metabolomics at the cell type level, validating the platform's utility in precisely dissecting cell-specific molecular networks.

Differential Expression Between Brain Regions Reveals Functional Insights

Beyond supporting differential analysis based on cell type annotations, SMIntegration's flexible interactive selection feature (Figure 1E) also allows researchers to directly select any region of interest to define comparison groups. We used the comparison between the cornu ammonis (CA) region and the mid-brain periaqueductal gray (PAG) region in a mouse brain coronal section (Figure S16, **Figure 4A**) as an example to demonstrate SMIntegration's flexible differential analysis to reveal regulatory networks in different brain microenvironments. Differential analysis identified 1484 differential genes (**Figure 4B**), corresponding to 213 pathways, and 193 differential metabolites, corresponding to 87 pathways. There were 55 pathways shared by both differential genes and metabolites (**Figure 4C**).

Figure 4D shows the pathways significantly enriched with these differential features. Genes and metabolites highly expressed in the CA region were significantly enriched in pathways such as Glutamatergic synapse, Neuroactive ligand-receptor interaction, and Long-term potentiation/depression. In particular, in the activated Glutamatergic synapse pathway (Figure S17A), L-Glutamic acid (Figure S17B) and *Grin2a* (Figure S17C) were upregulated. Glutamate is the main excitatory neurotransmitter in the hippocampus, and its metabolic flux directly affects synaptic plasticity. The gene *Grin2a* encodes the NMDAR, an ionotropic glutamate receptor. The synergistic action of these pathways forms the molecular basis of hippocampus-dependent learning and memory [33].

Pathways significantly enriched with genes and metabolites highly expressed in the PAG region point to its core functions: pain modulation and defensive responses. In the addiction-related neuroadaptations pathway (Figure 4E), both the metabolite gamma-Aminobutyric acid (GABA) and the gene *Slc32a1* were significantly upregulated (**Figure 4F**). *Slc32a1* encodes the vesicular transporter VGAT, which loads GABA into synaptic vesicles, and GABA is an inhibitory tonic [34]. Studies have shown that the analgesic effect of exogenous cannabinoids (e.g., Δ9-THC) in the PAG is achieved by activating CB1 receptors to modulate vesicular release patterns and reduce the probability of GABA release [34,35]. Furthermore, multi-feature imaging and spatial

co-expression analysis also demonstrated the consistent high expression of *Slc32a1* and GABA in the mid-brain region, predominantly the PAG (**Figure 4G**).

Conclusion

SMIntegration is the first zero-code platform for integrated spatial metabolomics and transcriptomics, unifying spatial pattern recognition, differential analysis, network construction, and functional annotation. It enables intuitive exploration of gene–metabolite interactions and spatial heterogeneity, as demonstrated in the mouse brain by revealing region- and cell type–specific networks and key processes such as GABA/Glutamate balance and cannabinoid signaling. Future extensions will include additional omics and machine learning–based modules, further advancing spatial multi-omics research in both fundamental and translational biology.

Institutional Review Board Statement

The animal study protocol was approved by the Institutional Review Board of BGI (protocol code BGI-IRB A25004 and date of approval 21 February 2025)

Data Availability Statement

All resources described in this study are publicly available. The raw spatial metabolomics and spatial transcriptomics data from mouse brain tissue, as well as the derived processed peak intensity tables, have been deposited in the China National Center for Bioinformation (CNCB) OMIX database. The spatial metabolomics and transcriptomics data are accessible under accession number OMIX011674 (https://ngdc.cncb.ac.cn/omix/release/OMIX011674, accessed on 1 September 2025).

Authors' contributions

Author Contributions (CRediT):

HKD: Conceptualization, Methodology, Software, Writing – Original Draft (Methods and Results)

XLN: Investigation, Formal Analysis, Writing – Original Draft (Introduction)

SQZ: Methodology, Data Curation, Software

LZ: Investigation, Resources

JW: Investigation, Resources

YZ: Investigation, Resources

CLY: Investigation, Resources

JZ: Supervision, Conceptualization

ZLM: Conceptualization, Writing – Review & Editing

All authors: Review & Approval of Final Manuscript

Competing interests

Haoke Deng, Xun Lin, Liang Zong, Shanqiao Zheng, Yun Zhao, Jing Wang, Jin Zi and Zhanlong Mei are employees of BGI Genomics. Xiaolian Ning and Lingyun Chen are employees of BGI Research. This paper reflects the views of the scientists, not the company.

Acknowledgments

This research was funded by the National Key R&D Program of China, grant number 2021YFA0805100, and the Sustainable Development Program of Shenzhen Science and Technology Major Program, grant number KCXFZ20240903093925033. During the preparation of this manuscript, the authors used Gemini 2.5 Flash (Experimental) to polish the language. The authors have reviewed and edited the generated content and take full responsibility for the content of this publication.

References

[1] Ståhl PL, Salmén F, Vickovic S, Lundmark A, Navarro JF, Magnusson J, et al. Visualization and analysis of gene expression in tissue sections by spatial transcriptomics. Science 2016; 353: 78−82.

[2] Eisenstein M. Seven technologies to watch in 2022. Nature 2022; 601: 658−61.

[3] Sun C, Wang A, Zhou Y, Chen P, Wang X, Huang J, et al. Spatially resolved multi-omics highlights cell-specific metabolic remodeling and interactions in gastric cancer. Nat Commun 2023; 14: 2692.

[4] Vicari M, Mirzazadeh R, Nilsson A, Shariatgorji R, Bjärterot P, Larsson L, et al. Spatial multimodal analysis of transcriptomes and metabolomes in tissues. Nat Biotechnol 2024; 42:1046–50.

[5] Ravi VM, Will P, Kueckelhaus J, Sun N, Joseph K, Salié H, et al. Spatially resolved multi-omics deciphers bidirectional tumor-host interdependence in glioblastoma. Cancer Cell 2022; 40: 639–55.e13.

[6] Zheng P, Zhang N, Ren D, Yu C, Zhao B, Zhang Y. Integrated spatial transcriptome and metabolism study reveals metabolic heterogeneity in human injured brain. Cell Rep Med 2023; 4: 101057.

[7] Long Y, Ang KS, Sethi R, Liao S, Heng Y, van Olst L, et al. Deciphering spatial domains from spatial multi-omics with SpatialGlue. Nat Methods 2024; 21: 1658–67.

[8] Dries R, Zhu Q, Dong R, Eng CHL, Li H, Liu K, et al. Giotto: a toolbox for integrative analysis and visualization of spatial expression data. Genome Biol 2021; 22: 78.

[9] Yang P, Jin L, Liao J, Jin K, Shao X, Li C, et al. Revealing spatial multimodal heterogeneity in tissues with SpaTrio. Cell Genomics 2023; 3: 100446.

[10] Yuan Z, Pan W, Zhao X, Zhao F, Xu Z, Li X, et al. SODB facilitates comprehensive exploration of spatial omics data. Nat Methods 2023; 20: 387–399.

[11] Wess M, Andersen MK, Midtbust E, Guillem JCC, Viset T, Størkersen Ø, et al. Spatial integration of multi-omics data from serial sections using the novel Multi-Omics Imaging Integration Toolset. Gigascience 2025; 14: giaf035.

[12] Coleman K, Schroeder A, Loth M, Zhang D, Park JH, Sung JY, et al. Resolving tissue complexity by multimodal spatial omics modeling with MISO. Nat Methods 2025; 22: 530–8.

[13] Wang H, Li J, Jing S, Lin P, Qiu Y, Yan X, et al. SOAPy: a Python package to dissect spatial architecture, dynamics, and communication. Genome Biol 2025; 26: 80.

[14] Dexter A, Thomas SA, Steven RT, Robinson KN, Taylor AJ, Elia EA, et al. A New Approach to Large Multiomics Data Integration. Anal Chem 2025; Advance online publication.

[15] Marconato L, Palla G, Yamauchi KA, Virshup I, Heidari E, Treis T, et al. SpatialData: an open and universal data framework for spatial omics. Nat Methods 2025; 22: 58–62.

[16] Liu Q, Hsu CY, Shyr Y. Scalable and model-free detection of spatial patterns and colocalization. Genome Res 2022; 32: 1736–45.

[17] Bivand R, Müller WG, Reder M. Power calculations for global and local Moran’s I. Comput Stat Data Anal 2009; 53: 2859–72.

[18] Stuart T, Butler A, Hoffman P, Hafemeister C, Papalexi E, Mauck WM, et al. Comprehensive Integration of Single-Cell Data. Cell 2019; 177: 1888–902.e21.

[19] Do VH, Canzar S. A generalization of t-SNE and UMAP to single-cell multimodal omics. Genome Biol 2021; 22: 130.

[20] Steinley D. K-means clustering: A half-century synthesis. Br J Math Stat Psychol 2006; 59: 1–34.

[21] Aran D, Looney AP, Liu L, Wu E, Fong V, Hsu A, et al. Reference-based analysis of lung single-cell sequencing reveals a transitional profibrotic macrophage. Nat Immunol 2019; 20: 163–172.

[22] Satija R, Farrell JA, Gennert D, Schier AF, Regev A. Spatial reconstruction of single-cell gene expression data. Nat Biotechnol 2015; 33: 495–502.

[23] Bemis KD, Harry A, Eberlin LS, Ferreira C, Van De Ven SM, Mallick P, et al. Cardinal: An R package for statistical analysis of mass spectrometry-based imaging experiments. Bioinformatics 2015; 31: 2418–20.

[24] Mei Z, Ning X, Deng H, Chen L, Zhao Y, Jin Z. SMAnalyst: A Web Server for Spatial Metabolomic Data Analysis and Annotation. Preprints 2025; 2025091621.

[25] Wang Q, Ding SL, Li Y, Royall J, Feng D, Lesnar P, et al. The Allen Mouse Brain Common Coordinate Framework: A 3D Reference Atlas. Cell 2020; 181: 936–53.e20.

[26] Carey H, Pegios M, Martin L, Saleeba C, Turner AJ, Everett NA, et al. DeepSlice: rapid fully automatic registration of mouse brain imaging to a volumetric atlas. Nat Commun 2023; 14: 5884.

[27] Puchades MA, Csucs G, Ledergerber D, Leergaard TB, Bjaalie JG. Spatial registration of serial microscopic brain images to three-dimensional reference atlases with the QuickNII tool. PLoS One 2019; 14: e0216796.

[28] Kandel ER. The molecular biology of memory: CAMP, PKA, CRE, CREB-1, CREB-2, and CPEB. Mol Brain 2012; 5:14.

[29] Pramod AB, Foster J, Carvelli L, Henry LK. SLC6 transporters: Structure, function, regulation, disease association and therapeutics. Mol Aspects Med 2013; 34: 197–219.

[30] Dikow N, Maas B, Karch S, Granzow M, Janssen JWG, Jauch A, et al. 3p25.3 microdeletion of GABA transporters SLC6A1 and SLC6A11 results in intellectual disability, epilepsy and stereotypic behavior. Am J Med Genet A 2014; 164A: 3061–8.

[31] Mu N, Wang Y, Li X, Du Z, Wu Y, Su M, et al. Crotonylated *BEX2* interacts with NDP52 and enhances mitophagy to modulate chemotherapeutic agent-induced apoptosis in non-small-cell lung cancer cells. Cell Death Dis 2023; 14: 645.

[32] Chamoto K, Zhang B, Tajima M, Honjo T, Fagarasan S. Spermidine – an old molecule with a new age-defying immune function. Trends Cell Biol 2024; 34: 363–70.

[33] McNair LM, Andersen JV, Waagepetersen HS. Stable isotope tracing reveals disturbed cellular energy and glutamate metabolism in hippocampal slices of aged male mice. Neurochem Int 2023; 171: 105626.

[34] Keay KA, Bandler R. Parallel circuits mediating distinct emotional coping reactions to different types of stress. Neurosci Biobehav Rev 2001; 25: 669–78.

[35] Finn DP, Jhaveri MD, Beckett SRG, Roe CH, Kendall DA, Marsden CA, et al. Effects of direct periaqueductal grey administration of a cannabinoid receptor agonist on nociceptive and aversive responses in rats. Neuropharmacology 2003; 45: 594–604.

Figure legends

Figure 1 Overall architecture of SMIntegration

A. Data Upload. **B.** Spatial pattern analysis, identifying features with specific spatial patterns. **C.** Clustering analysis. **D.** Cell type annotation analysis. **E.** Differential analysis, allowing the selection of corresponding differential features through three different methods. **F.** Functional association analysis of differential features. **G.** Imaging visualization.

Figure 2 Integrated multi-omics clustering identifies conserved spatial domains and correlated molecular patterns

A. Schematic of mouse brain structure (atlas from ABA_Mouse_CCFv3_2017_25um.cutlas). **B.** Spatial metabolomics clustering. **C.** Spatial transcriptomics clustering. **D.** Integrated two-omics clustering map. **E.** Spatial pattern identification from spatial transcriptomics data. **F.** Spatial pattern identification from spatial metabolomics data. **G.** Spatial correlation between spatial metabolomics and spatial transcriptomics patterns. (The spatial pattern maps in B-F include brain region outlines for easier identification.)

Figure 3 Cell type-based differential analysis reveals cell-enriched metabolites and genes.

A. Group comparison selection based on cell types, with the experimental group being non-telencephalon astrocytes (NA) and the control group being mature oligodendrocytes (MO). **B.** UMAP analysis of genes in the MO and NA regions. **C.** UMAP analysis of metabolites in the MO and NA regions. **D.** Number of differentially expressed genes and metabolites in MO and NA. **E.** Correlation network between differential metabolites and genes in the NA region. **F.** Correlation network between differential metabolites and genes in the MO region.

Figure 4 Interactive spatial selection unveils region-enriched multi-omics functional pathways

A. Group comparison selection based on brain regions, with the experimental group as cornu ammonis (CA) and the control group as periaqueductal gray (PAG). **B.** Number

of differentially expressed genes and metabolites in the CA and PAG regions. **C.** Number of annotated pathways for differential genes and metabolites in the CA and PAG regions. **D.** Pathway enrichment for differential features in the CA and PAG regions. **E.** Pathway Retrograde endocannabinoid signaling enriched with metabolites and genes in the PAG region. **F.** Abundance distribution maps of enriched genes and metabolites in the PAG region. **G.** Spatial co-imaging (left) and co-expression (right) of *Slc32a1* and GABA.

Supplementary material

Supplementary File 1 Spatial metabolomics and transcriptomics data registration tutorial

Table S1 Execution time and memory consumption across datasets of varying sizes

Figure S1 Screenshot of the SMIntegration tutorial interface

Figure S2 Schematic diagram of data input formats.

A. Text Matrix Format: Requires metabolomics/transcriptomics data to be submitted as a "feature-pixel" matrix, with each column containing the metabolite/gene name, spatial coordinates (x/y), and feature value. **B.** Seurat Object Format: Requires both spatial metabolomics and transcriptomics data to share identical structures. The feature-pixel matrix is stored in the Spatial$counts slot (rows represent features and columns represent pixels), while spatial coordinates are stored in the meta.data slot.

Figure S3 Screenshot of the data upload interface

Figure S4 Screenshot of the spatial pattern analysis interface

Figure S5 Screenshot of the spatial clustering interface

Figure S6 Cell type annotation

A. Screenshot of the cell type annotation interface. **B.** Requirements for user-defined annotation file format: Must be a text matrix with three columns: spatial x/y coordinates and cell type.

Figure S7 Screenshot of the differential analysis region selection interface

A. Use the lasso tool to select ROI, then assign it as the experimental or control group. **B.** Select clustering classes, then assign them as the experimental or control group. **C.** Select cell classes, then assign them as the experimental or control group.

Figure S8 Screenshot of the differential analysis interface

Figure S9 Screenshot of the group-specific network interface

Figure S10 Screenshot of the functional association analysis interface

Figure S11 Screenshot of the single-feature visualization interface

Figure S12 Screenshot of the multi-feature visualization interface

Figure S13 Mouse brain data registration workflow

A. Total abundance imaging of spatial transcriptomics and spatial metabolomics data before registration.

B. Total abundance imaging of spatial transcriptomics and spatial metabolomics data after registration.

Figure S14 Enriched pathways in the conserved metabolite pattern 3 and gene expression pattern 2

Figure S15 Annotation of enriched features in non-telencephalic astrocytes within the GABAergic synapse pathway

A. Molecular annotation in the GABAergic synapse pathway. **B.** The spatial distribution of Gamma-aminobutyric acid (GABA) abundance. **C.** The spatial distribution of *Slc6a11* expression level.

Figure S16 Schematic diagram of manual selection for CA vs PAG

Figure S17 Annotation of enriched features in CA region within the glutamatergic synapse pathway

A. Molecular annotation in the glutamatergic synapse pathway. **B.** The spatial distribution of L-Glutamic acid abundance. **C.** The spatial distribution of *Grin2a* expression level.

merged figures

Click here to access/download;Figure;alt-text-figure-accessibility.pdf

Figure accessibility and alt text

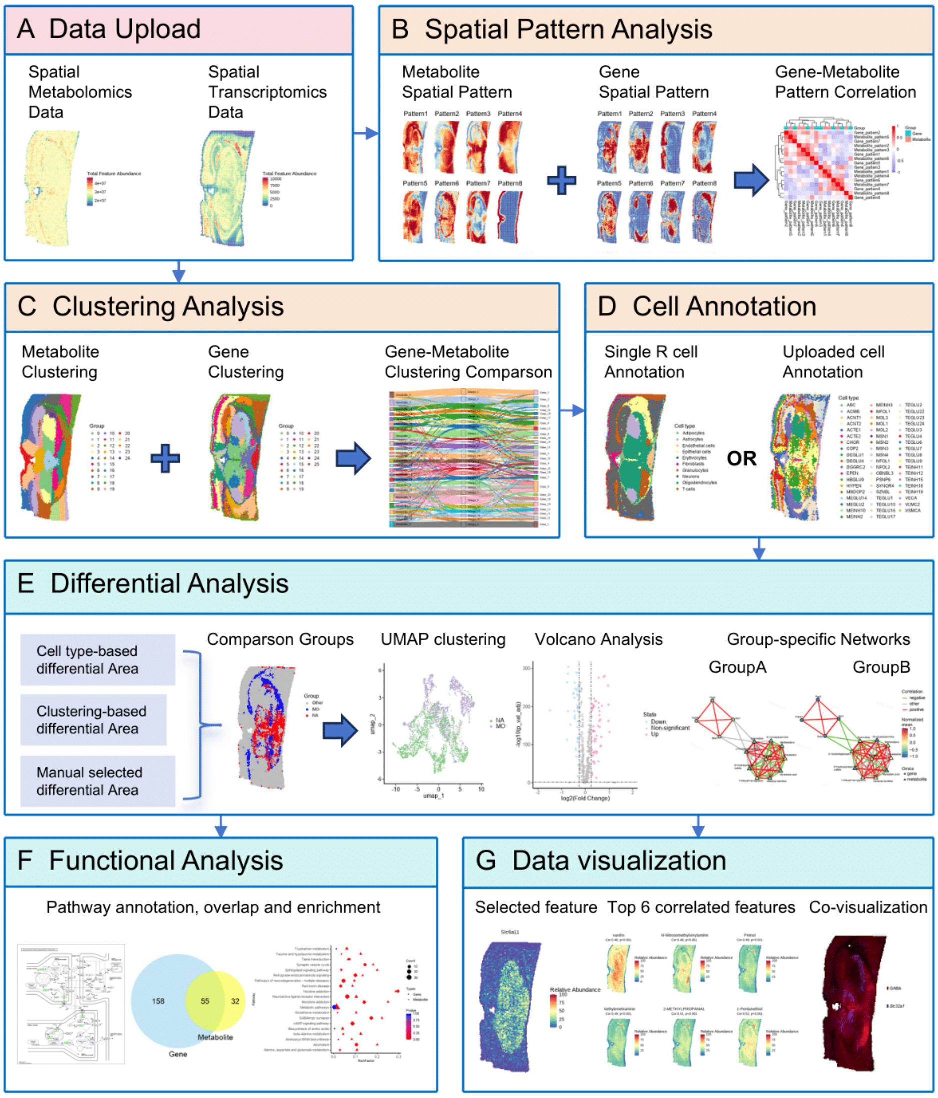

Figure 1 Overall architecture of SMIntegration

A. Data Upload. **B.** Spatial pattern analysis, identifying features with specific spatial patterns. **C.** Clustering analysis. **D.** Cell type annotation analysis. **E.** Differential analysis, allowing the selection of corresponding differential features through three different methods. **F.** Functional association analysis of differential features. **G.** Imaging visualization.

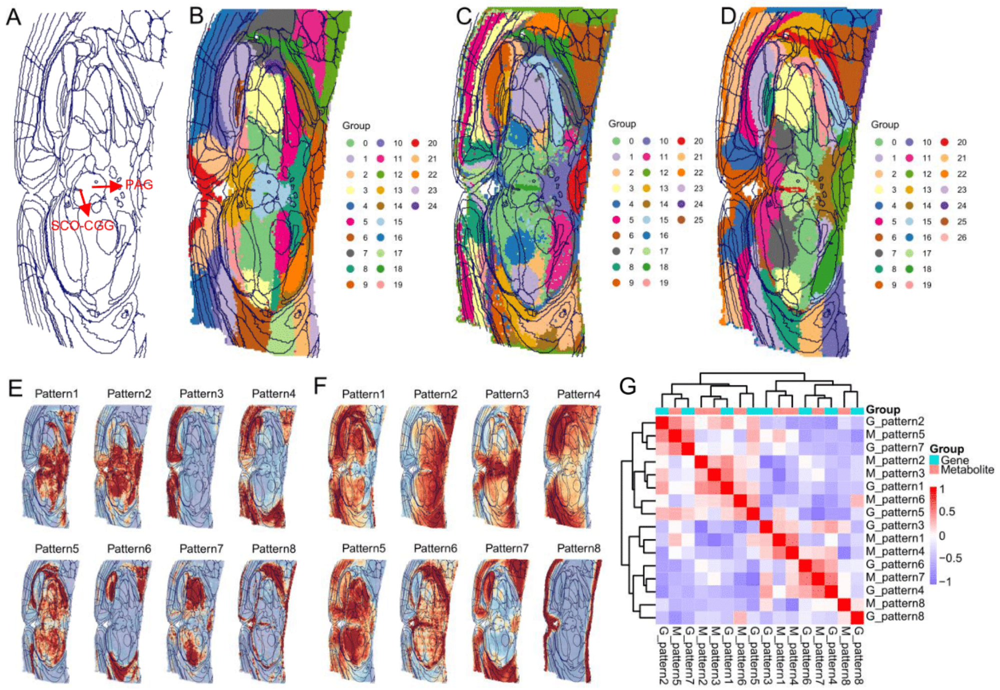

Figure 2 Integrated multi-omics clustering identifies conserved spatial domains and correlated molecular patterns

A. Schematic of mouse brain structure (atlas from ABA_Mouse_CCFv3_2017_25um.cutlas). **B.** Spatial metabolomics clustering. **C.** Spatial transcriptomics clustering. **D.** Integrated two-omics clustering map. **E.** Spatial pattern identification from spatial transcriptomics data. **F.** Spatial pattern identification from spatial metabolomics data. **G.** Spatial correlation between spatial metabolomics and spatial transcriptomics patterns. (The spatial pattern maps in B-F include brain region outlines for easier identification.)

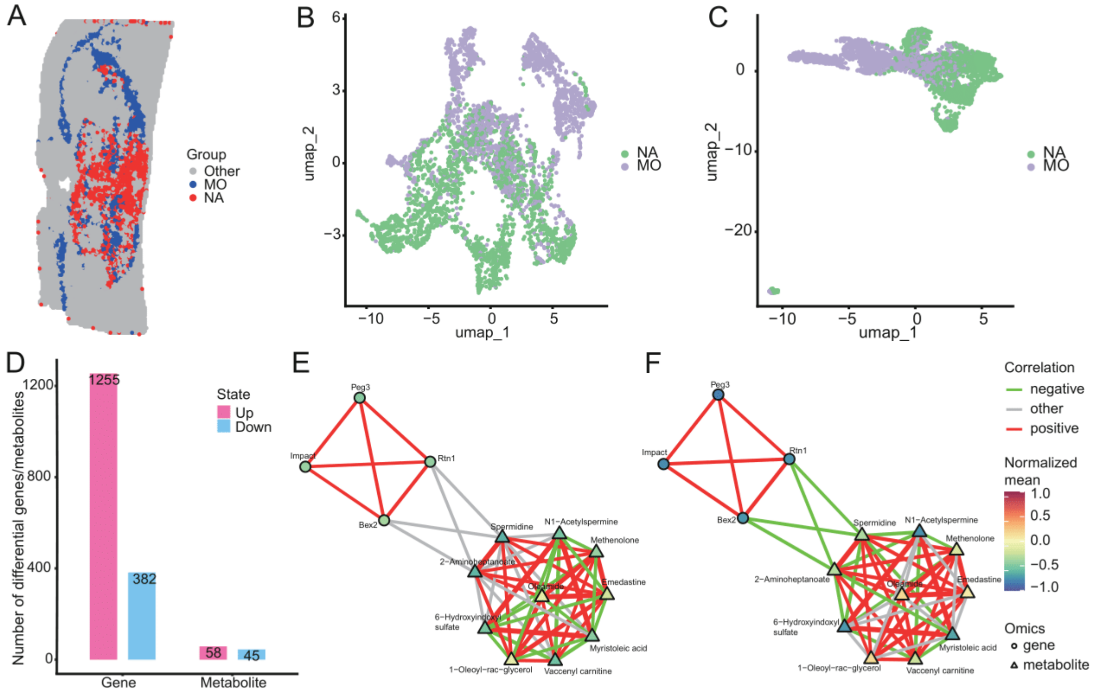

Figure 3 Cell type-based differential analysis reveals cell-enriched metabolites and genes.

A. Group comparison selection based on cell types, with the experimental group being non-telencephalon astrocytes (NA) and the control group being mature oligodendrocytes (MO). **B.** UMAP analysis of metabolites in the MO and NA regions. **C.** UMAP analysis of genes in the MO and NA regions. **D.** Number of differentially expressed genes and metabolites in MO and NA. **E.** Correlation network between differential metabolites and genes in the NA region. **F.** Correlation network between differential metabolites and genes in the MO region.

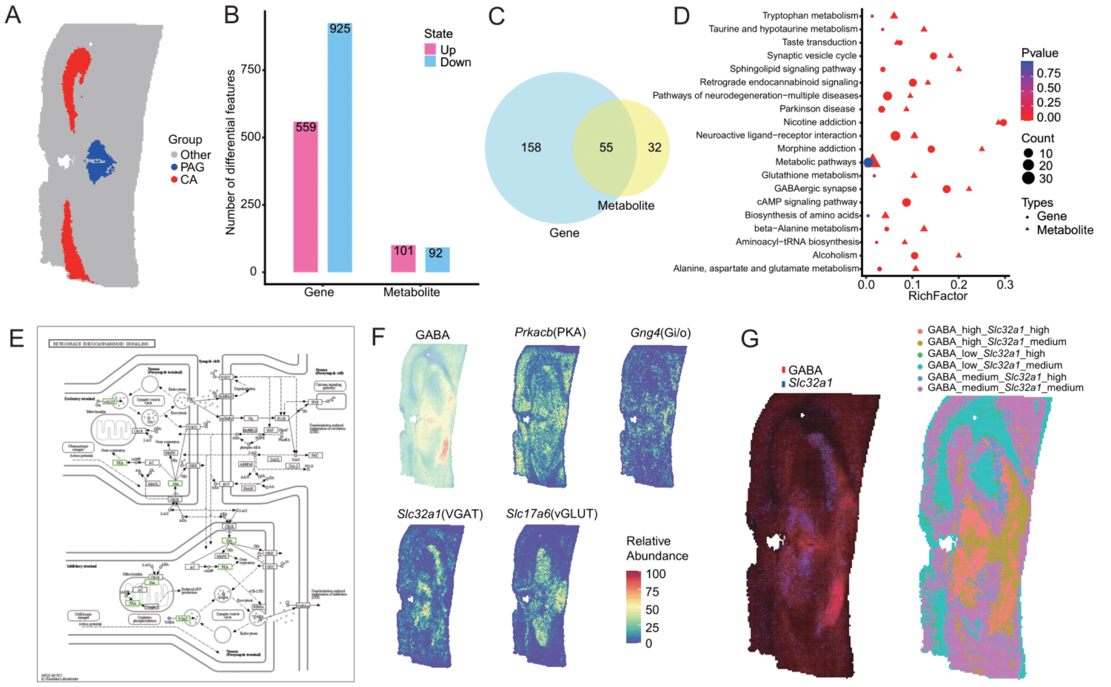

Figure 4 Interactive spatial selection unveils region-enriched multi-omics functional pathways

A. Group comparison selection based on brain regions, with the experimental group as cornu ammonis (CA) and the control group as periaqueductal gray (PAG). **B.** Number of differentially expressed genes and metabolites in the CA and PAG regions. **C.** Number of annotated pathways for differential genes and metabolites in the CA and PAG regions. **D.** Pathway enrichment for differential features in the CA and PAG regions. **E.** Pathway Retrograde endocannabinoid signaling enriched with metabolites and genes in the PAG region. **F.** Abundance distribution maps of enriched genes and metabolites in the PAG region. **G.** Spatial co-imaging (left) and co-expression (right) of *Slc32a1* and GABA.

Figure 1 Overall architecture of SMIntegration

Click here to access/download;Figure;figure1.pdf

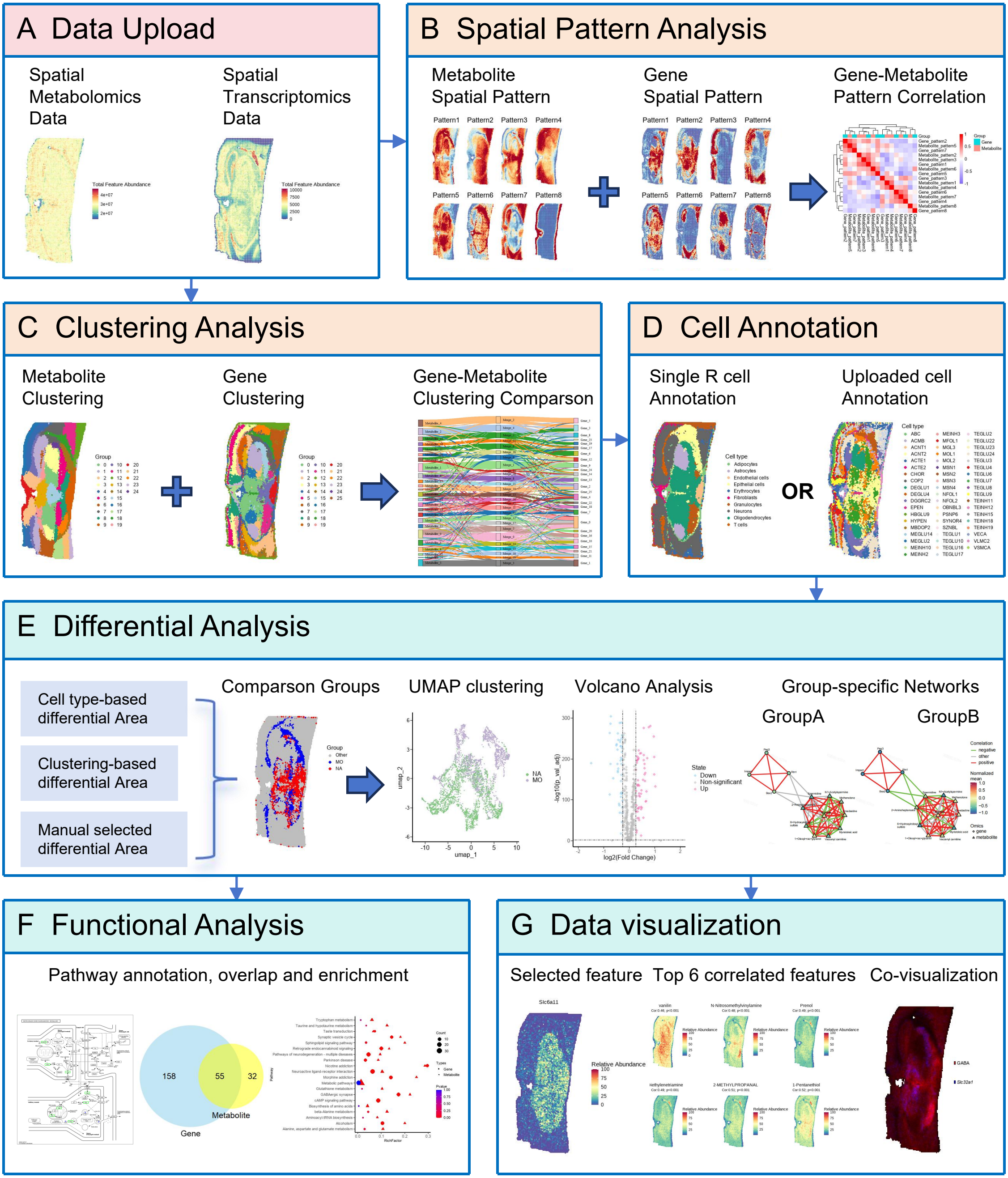

Figure 2 Integrated multi-omics clustering identifies conserved spatial domains and correlated molecular

Click here to access/download;Figure;figure2.pdf

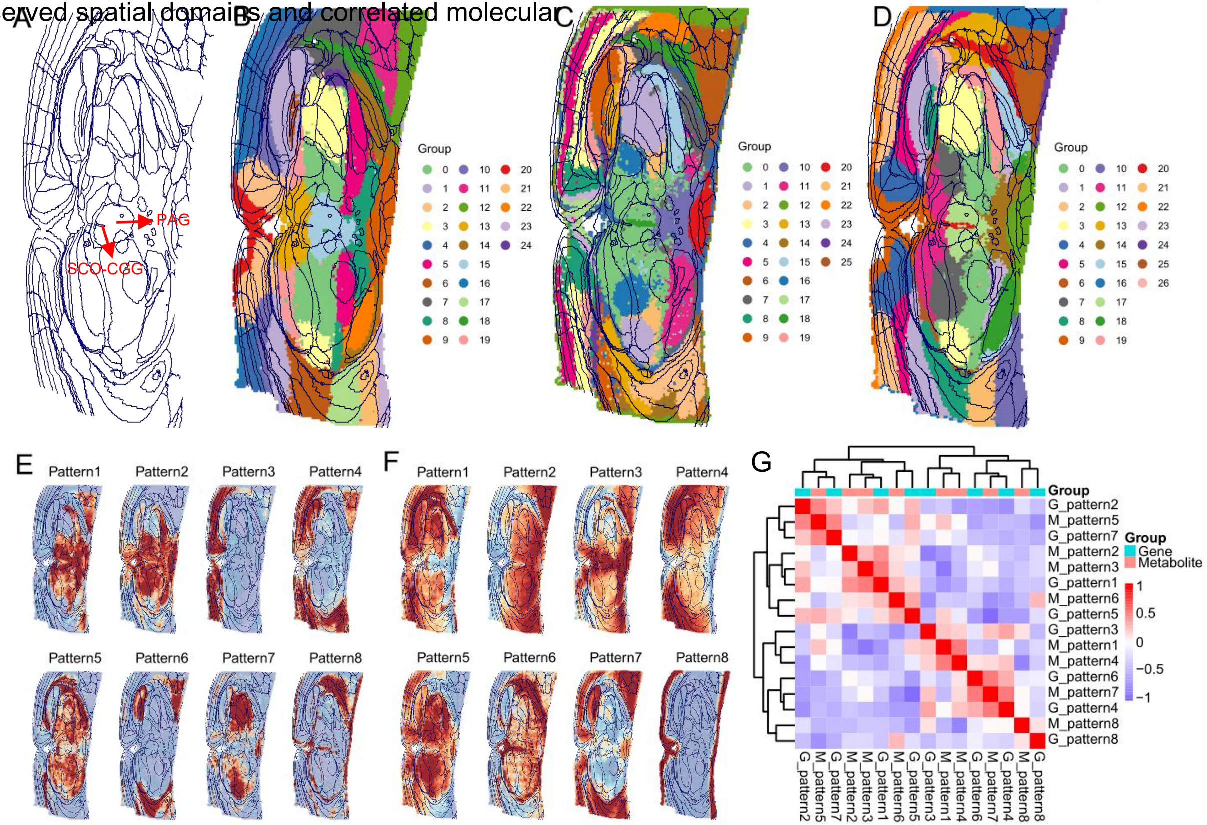

Figure 3. Cell type-based differential analysis reveals cell-enriched metabolites and genes.

Click here to access/download;Figure;figure3.pdf

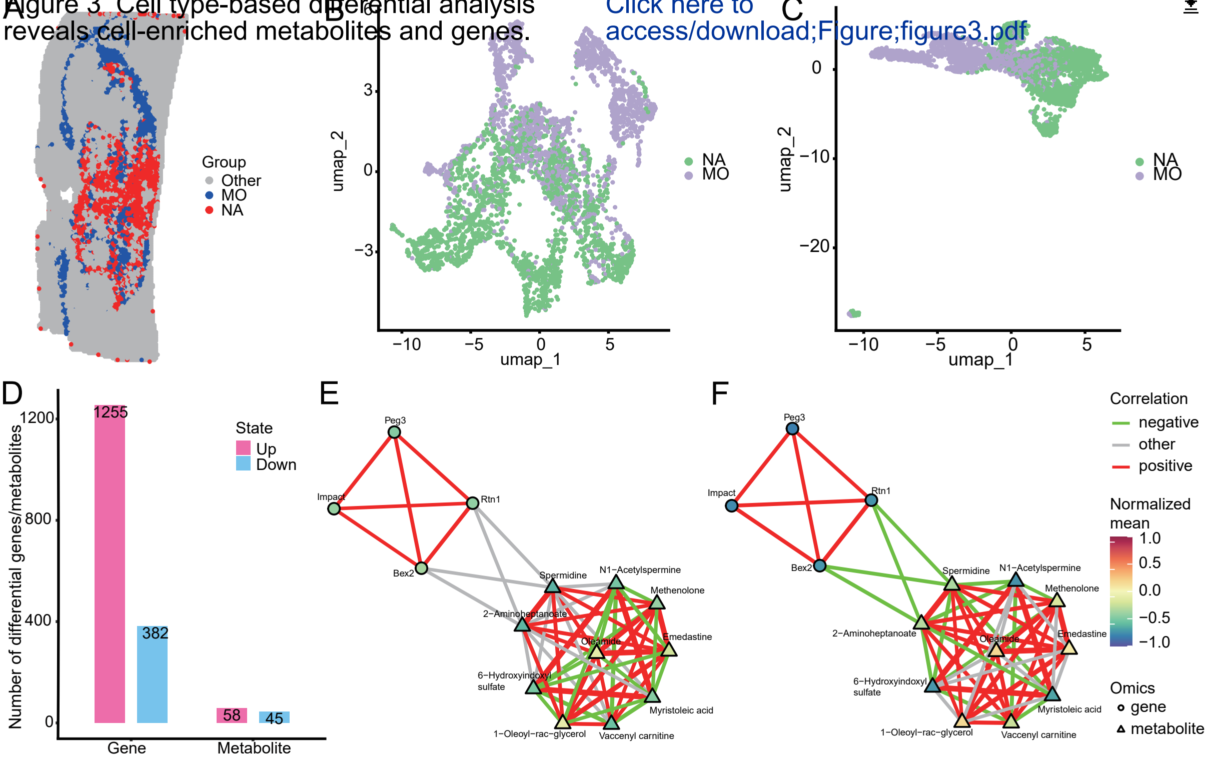

Click here to access/download;Figure;figure4.pdf

Figure 4 Interactive spatial selection unveils region-enriched multi-omics functional pathways

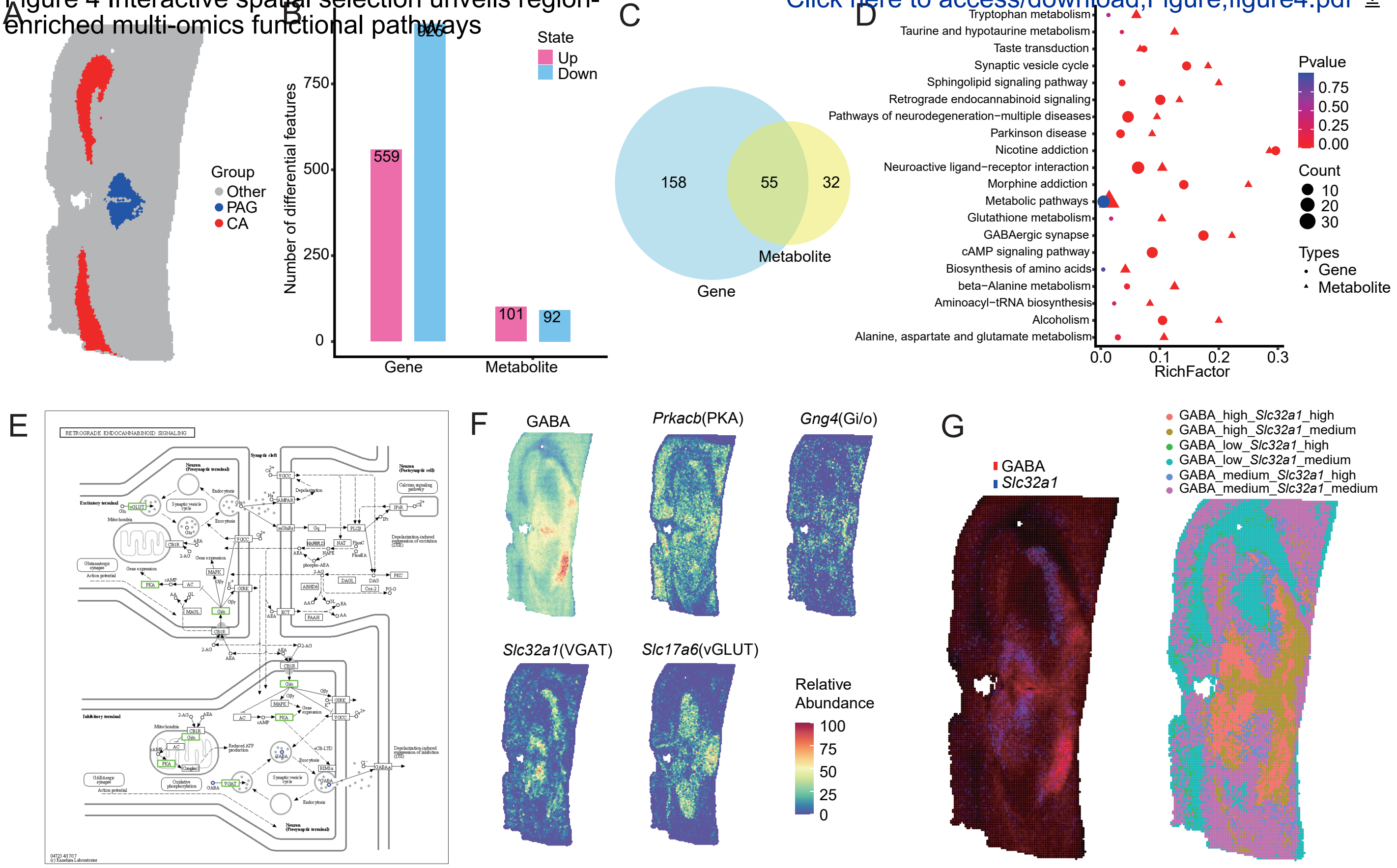

Supplementary File 1 Spatial metabolomics and transcriptomics data registration tutorial

Click here to access/download
Supplementary Material
Supplementary_File_1.pdf

Table S1 Execution time and memory consumption across datasets of varying sizes

Click here to access/download
Supplementary Material
Table_S1.xlsx

Figure S1 Screenshot of the SMIntegration tutorial interface

Click here to access/download

Supplementary Material

Figure_S1.pdf

Figure S2 Schematic diagram of data input formats.

Click here to access/download
Supplementary Material
Figure_S2.pdf

Figure S3 Screenshot of the data upload interface

Click here to access/download
Supplementary Material
Figure_S3.pdf

Figure S4 Screenshot of the spatial pattern analysis interface

Click here to access/download
Supplementary Material
Figure_S4.pdf

Figure S5 Screenshot of the spatial clustering interface

Click here to access/download
Supplementary Material
Figure_S5.pdf

Figure S6 Cell type annotation

Click here to access/download
Supplementary Material
Figure_S6.pdf

Figure S7 Screenshot of the differential analysis region selection interface

Click here to access/download
Supplementary Material
Figure_S7.pdf

Figure S8 Screenshot of the differential analysis interface

Click here to access/download

Supplementary Material

Figure_S8.pdf

Figure S9 Screenshot of the group-specific network interface

Click here to access/download
Supplementary Material
Figure_S9.pdf

Figure S10 Screenshot of the functional association analysis interface

Click here to access/download
Supplementary Material
Figure_S10.pdf

Figure S11 Screenshot of the single-feature visualization interface

Click here to access/download
Supplementary Material
Figure_S11.pdf

Figure S12 Screenshot of the multi-feature visualization interface

Click here to access/download

Supplementary Material

Figure_S12.pdf

Figure S13 Mouse brain data registration workflow

Click here to access/download
Supplementary Material
Figure_S13.pdf

Figure S14 Enriched pathways in the conserved metabolite pattern 3 and gene expression pattern 2

Click here to access/download
Supplementary Material
Figure_S14.pdf

Figure S15 Annotation of enriched features in non-telencephalic astrocytes within the GABAergic synapse pathway

Click here to access/download
Supplementary Material
Figure_S15.pdf

Figure S16 Schematic diagram of manual selection for CA vs PAG

Click here to access/download
Supplementary Material
Figure_S16.pdf

Figure S17 Annotation of enriched features in CA region within the glutamatergic synapse pathway

Click here to access/download
Supplementary Material
Figure_S17.pdf

coverletter for gigascience Click here to access/download;Personal Cover;Cover_Letter_gigascience.docx

Cover Letter

November 20, 2025

Submission Type: Technical Note

Dr. Hongling Zhou

Editor, GigaScience

Dear Dr. Zhou,

On behalf of my co-authors, we are pleased to submit our manuscript entitled "SMIntegration: A Web Platform for Integrated Spatial Metabolomics and Transcriptomics Analysis" for consideration for publication in GigaScience as a Technical Note.

Spatial multiomics provides powerful insights into cellular heterogeneity and molecular interactions within complex tissues. However, current computational tools often lack the capacity to integrate multiple data modalities in a user-friendly and interpretable manner. To bridge this gap, we developed SMIntegration, a dedicated web-based graphical platform that enables integrated analysis of spatial metabolomics and transcriptomics data.

Built using R and Shiny and containerized with Docker, SMIntegration offers a comprehensive workflow-from data preprocessing to functional annotation. Key features include cross-modal spatial pattern recognition, flexible differential analysis across custom regions or annotated cell types, and interactive construction of group-specific gene-metabolite networks. Using adjacent mouse brain sections profiled by Stereo-seq transcriptomics and AFADESI-MS metabolomics, we demonstrate that SMIntegration uncovers spatial domains and metabolic-transcriptional modules that are not detectable through single-modality analysis. The platform's zero-code interface makes it accessible to researchers across disciplines, facilitating exploration of cross-omics interactions in development, homeostasis, and disease contexts.

We believe our work closely matches the scope and mission of GigaScience, by providing an innovative methodology and an accessible computational tool for spatial multiomics data integration and interpretation. We are confident that this contribution will appeal to GigaScience readers engaged in both methodological and applied research.

This manuscript represents original work, has not been published before, and is not currently under consideration elsewhere. All authors have approved the manuscript and its submission to GigaScience.

Thank you for your time and consideration. We look forward to hearing from you.

Sincerely,

Corresponding Authors:

Jin Zi
BGI, Shenzhen 518083, China
Email: zij@genomics.cn
Phone: +86-18118761065

Zhanlong Mei
BGI, Shenzhen 518083, China
Email: meizhanlong@genomics.cn
Phone: +86-15813736269

gpb-coverletter Click here to access/download;Personal Cover;Cover_Letter_SMintegration.docx

Dear Editor,

On behalf of my co-authors, we are pleased to submit our manuscript entitled "SMIntegration: A Web Platform for Integrated Spatial Metabolomics and Transcriptomics Analysis" for consideration in the GigaScience Special Issue on Spatial Multiomics (article type: Web Server).

Spatial multiomics offers unique opportunities for understanding cellular heterogeneity and molecular interactions in complex tissues, but current computational resources gpbbiological interpretation. To address these challenges, we developed SMIntegration, the first dedicated, web-based graphical platform for the integrated analysis of spatial metabolomics and transcriptomics data.

The platform, implemented in R/Shiny and containerized with Docker, provides a complete workflow from preprocessing to functional annotation. Its distinctive features include cross-modal spatial pattern recognition, flexible differential analysis across user-defined regions or cell-type annotations, and interactive construction of group-specific gene–metabolite networks. Using adjacent mouse brain coronal sections profiled by Stereo-seq transcriptomics and AFADESI-MS metabolomics, SMIntegration successfully identified spatial domains and metabolic–transcriptional modules that were overlooked by single-modality analyses. Importantly, the platform features a zero-code interface, enabling researchers with diverse backgrounds to explore cross-omics interactions during development, homeostasis, and disease.

We believe that this work fits well within the scope of the Special Issue, as it provides a methodological advancement and an accessible computational tool for spatial multiomics data integration and interpretation. We are confident that GigaScience

readers will find this contribution valuable for both methodological and applied research.

This manuscript is original, has not been published previously, and is not under consideration elsewhere. All authors have approved the submission. We kindly request that it be considered for inclusion in the Special Issue on Spatial Multiomics.

Thank you very much for your time and consideration. We look forward to your feedback.

Sincerely,

Corresponding Authors:
Jin Zi
BGI, Shenzhen 518083, China
Email: zij@genomics.cn

Phone: +86-18118761065

Zhanlong Mei
BGI, Shenzhen 518083, China
Email: meizhanlong@genomics.cn
Phone: +86-15813736269

**Date:**	Oct 16, 2025
**To:**	"Zhanlong Mei" meizhanlong@genomics.cn
**From:**	"Genomics Proteomics and Bioinformatics" editor@big.ac.cn
**Subject:**	Genomics, Proteomics & Bioinformatics - GPB-D-25-00503 - Decision

Ms. Ref. No.: GPB-D-25-00503
Title: SMIntegration: A Web Tool for Comprehensive Spatial Metabolomics and Transcriptomics Integrated Analysis and Visualization
Genomics, Proteomics & Bioinformatics

Dear Dr. Zhanlong Mei,

I'm writing to you regarding manuscript GPB-D-25-00503 entitled "SMIntegration: A Web Tool for Comprehensive Spatial Metabolomics and Transcriptomics Integrated Analysis and Visualization" which you submitted to the Genomics, Proteomics & Bioinformatics.

I'd like to thank you for your interest in publishing in Genomics, Proteomics & Bioinformatics but regret to inform you that your manuscript has not been selected for publication in the journal. While we will not be considering the manuscript further for publication in our journal we would like to offer you the opportunity to transfer your submission to an alternative journal via the OUP Transfer Desk.

Our partners in the OUP Transfer Desk, **Straive (OUPTransferAssistant@straive.com)**, will be in touch within the next 24-48 hours with details of how the transfer service works together with a list of alternative submission venues for your manuscript. Accepting a transfer offer is entirely optional; the Transfer Desk will never transfer your manuscript to another journal without your permission. Please note that accepting a transferred article is entirely at the discretion of the receiving editors, who maintain editorial independence.

Thank you for considering the Genomics, Proteomics & Bioinformatics for the publication of your research. I hope the outcome of this specific submission will not discourage you from the submission of future manuscripts.

Yuxia Jiao
GPB Editorial Office
Genomics, Proteomics & Bioinformatics
