# Supplementary material for: SMIntegration: A web tool for comprehensive spatial metabolomics and transcriptomics integrated analysis and visualization
Source: Gigascience. 2026 Mar 24;15:giag033. doi: 10.1093/gigascience/giag033 (PMC13159472; doi:10.1093/gigascience/giag033)
Supplement: giag033_GIGA-D-25-00440_revision_1 [file giag033_giga-d-25-00440_revision_1.pdf]

GigaScience

SMIntegration: A Web Tool for Comprehensive Spatial Metabolomics and Transcriptomics Integrated Analysis and Visualization

--Manuscript Draft--

**Manuscript Number:**	GIGA-D-25-00440R1
**Full Title:**	SMIntegration: A Web Tool for Comprehensive Spatial Metabolomics and Transcriptomics Integrated Analysis and Visualization
**Article Type:**	Technical Note
**Funding Information:**	the National Key R&D Program of China (2021YFA0805100) — Not applicable<br>Major Scientific and Technological Special Project of Guizhou Province (KCXFZ20240903093925033) — Not applicable
**Abstract:**	Current tools for spatial omics analysis often face challenges in performing integrated transcriptomics and metabolomics analysis, in-depth biological interpretation, and user-friendly operation. To address this, we developed SMIntegration, the first web-based graphical platform designed specifically for integrated spatial metabolomics and transcriptomics analysis. Built with R/Shiny and deployed using Docker containerization, the platform provides a complete integration workflow, starting from pre-processed spatial features through to functional annotation. Its core functions include: (1) automated and interactive spatial registration; (2) cross-modal spatial pattern recognition; (3) flexible differential analysis of genes and mass features based on clustering results, user-defined regions, or cell type annotations; and (4) group-specific gene-metabolite network construction and interactive visualization. Using adjacent mouse brain coronal sections (Stereo-seq transcriptomics and AFADESI-MS metabolomics) as an example, SMIntegration successfully identified both the periaqueductal gray and subcommissural organ, which were missed by single-modality clustering. Cell type analysis revealed an association between astrocyte-enriched GABA metabolism and Slc6a11, while a comparison between the cornu ammonis region and the midbrain periaqueductal gray dissected glutamatergic and endogenous cannabinoid signaling pathway modules. With a zero-code interface, SMIntegration enables a wide range of researchers to deeply explore gene-metabolite interaction mechanisms within microenvironments during development, homeostasis, and disease.
**Corresponding Author:**	Zhanlong Mei, Ph.D.<br>BGI-Shenzhen: BGI Group<br>Shenzhen, Guangdong CHINA
**Corresponding Author Secondary Information:**	
**Corresponding Author's Institution:**	BGI-Shenzhen: BGI Group
**Corresponding Author's Secondary Institution:**	
**First Author:**	Haoke Deng
**First Author Secondary Information:**	
**Order of Authors:**	Haoke Deng
	Xiaolian Ning
	Xun Lin
	Liang Zong
	Shanqiao Zheng
	Yun Zhao
	Jing Wang

Powered by Editorial Manager® and ProduXion Manager® from Aries Systems Corporation

	Lingyun Chen
	Jin Zi
	Zhanlong Mei
**Order of Authors Secondary Information:**	
**Response to Reviewers:**	# Reviewer 1<br><br>1.Summary<br>We would like to express our sincere gratitude to the reviewer for their critical and constructive feedback. These comments have been instrumental in identifying key barriers to usability and technical reproducibility in our initial submission. In response, we have performed a major overhaul of the SMIntegration platform, codebase, and manuscript. The key highlights of our revisions are summarized below:<br>1)Integrated Spatial Registration: Addressing the critical concern regarding the difficulty of data coregistration, we have implemented a dedicated Spatial Registration module within the SMIntegration GUI. Utilizing the RNiftyReg framework, this module allows users to perform both linear and non-linear transformations directly within the platform, eliminating the need for undocumented external scripts.<br>2)Enhanced Analytical Rigor and Transparency: We have refined our analytical strategies to ensure robustness and clarity. This includes: (1) integrating PCA-based visualization to objectively guide clustering parameter selection; (2) justifying our differential analysis thresholds based on spatial biological sensitivity; and (3) transitioning from "functional enrichment" to a more transparent "Pathway Annotation" workflow to address the specific challenges of spatial metabolomics data interpretation.<br>3)Comprehensive Documentation and Validation: We have completely restructured our GitHub repository to meet high software standards. This includes systematic code commenting for all scripts and the implementation of a dual-layer validation suite (modular unit tests and figure reproduction scripts) to ensure computational reliability. Additionally, we have expanded user resources with step-by-step tutorials and integrated help manuals.<br>4)Refined Scientific Interpretation: We have carefully revised the manuscript's language to better reflect the exploratory nature of our findings, replacing over-interpretative terms with more precise descriptions of observed spatial patterns and associations.<br>We believe these substantial improvements effectively lower the technical barriers for users and establish SMIntegration as a rigorous, user-friendly tool for the spatial omics community. Detailed responses to each of the reviewer's comments are provided below.<br>2. Point-by-point response to Comments and Suggestions for Authors<br>Comment 1: "First, the software requires metabolomics and transcriptomics data which has already been coregistered and transformed to the same spatial resolution. These two tasks - especially coregistration - are challenging and a barrier to researchers intending to use the presented package. The authors point to a script on their github to coregister data, but this has zero documentation so is not a viable option. Please give better instructions and point to other options for these tasks."<br>Response: We sincerely thank the reviewer for this critical feedback. We agree that data coregistration and resolution alignment are significant steps in multi-omics workflows. We have taken the following actions to address these concerns:<br>1. Lowering the Barrier via Integrated Registration To eliminate the need for external, undocumented scripts, we have supplemented the Overall Distribution Analysis module with a robust registration function. When users select the 'Perform Registration' option, a comprehensive registration interface is presented directly within the GUI. This function utilizes the RNiftyReg package, providing an intuitive interface for both linear and non-linear (free-form deformation) transformations. This allows users to align different tissue sections or modalities within a single, unified web environment.<br>2. Standardized Resolution Alignment (Binning) Workflow: We would like to clarify that transforming different modalities to a common spatial resolution is now a standard and well-established operation in the spatial omics field. High-resolution spatial transcriptomics data (e.g., Stereo-seq at 500 nm resolution) is routinely aggregated or "binned" to reduce technical noise and match the resolution of other modalities [16]. SMIntegration follows this industry-standard practice by providing clear guidelines and internal logic for aggregating higher-resolution data to match the lower-resolution grid of spatial metabolomics (e.g., 50 μm).

Powered by Editorial Manager® and ProduXion Manager® from Aries Systems Corporation

3. Comprehensive Documentation and Tutorials: We have significantly improved our external resources and internal "Help" interface:
4.We have replaced the previous scripts with a comprehensive step-by-step tutorial (now included as Supplementary File 1). A new screenshot-based manual is available within the platform to guide users through the integrated registration process.

Specific changes made in the manuscript:
Abstract: Highlighted the addition of the "automated and interactive spatial registration" module. Revised text: "Its core functions include: (1) automated and interactive spatial registration; (2) cross-modal spatial pattern recognition; (3) flexible differential analysis of genes and mass features based on clustering results, user-defined regions, or cell type annotations; and (4) group-specific gene-metabolite network construction and interactive visualization."(Page 3, Line 55)
Methods: Revised the "Data Preparation and Spatial Registration" section to describe the RNiftyReg-based module and the standard binning procedures. Revised text: " SMIntegration input requires processed spatial metabolomics and transcriptomics data matrix. Since resolutions differ, higher-resolution data should be aggregated to match the lower (e.g., binning 500 nm transcriptomics by 100 to 50 μm metabolomics) [16]. To facilitate seamless integration, SMIntegration features a dedicated spatial registration module implemented using the RNiftyReg package [17], which provides an R interface to the NiftyReg library [18,19] and is part of the TractoR framework [20]. This module supports both linear (block-matching) and non-linear (free-form deformation) transformations to align the two modalities. Detailed tutorials for this registration module are provided in Supplementary File 1. Users also have the option to perform coregistration externally using the Python-based SpatialData ecosystem [21]; the aligned data can then be imported into SMIntegration in a compatible format for subsequent integrated analysis. Two input formats are supported: (1) text matrices containing feature name, spatial x/y coordinates, and values (Figure S2A); (2) Seurat objects with coordinates and abundance in designated slots (Figure S2B). Upload requirements are detailed on the help page."(Page 6, Line 131)
Figures & Supplementary Material: Updated Figure 1 and added Supplementary File 1 to serve as a complete user manual, and updated Figure S1 and Figure S3 to showcase the enhanced platform interface.
References: We have added five new references [16-20] to acknowledge the developers of the registration library and the TractoR framework.
We believe these updates effectively address the technical barriers mentioned and ensure that SMIntegration provides a complete, user-friendly pipeline from raw data to biological insight.

Comment 2: " The methods employed require various parameters to be set. For example, the number of clusters or clustering resolution. How should users set these? It would be nice to provide some (semi)automatic methods for picking the best values (or at least objective assessment of the results, allowing different values to be compared). Defaults should be justified / explained. E.g. why is the default log2FC threshold 2.6? "
Response: We sincerely thank the reviewer for these insightful comments regarding parameter transparency and objective guidance. We agree that providing a basis for parameter selection is crucial for reproducible and rigorous spatial omics analysis. We have addressed these concerns from the following aspects:
1. Objective Guidance for Clustering Parameters via PCA. In response to the suggestion for more objective methods (and as detailed in our response to Comment 5), we have integrated Principal Component Analysis (PCA) into the clustering workflow. Users can now utilize PCA variance plots to evaluate the complexity of their datasets. By observing the "elbow" of the variance curve, users can objectively determine the number of dimensions or clusters that best represent the spatial variation in their data, rather than relying on arbitrary defaults.
2. Justification of the |log_2FC| > 0.26 Default Threshold. We would like to clarify that the default threshold is 0.26 (approximately a 1.2-fold change), not 2.6. We chose this value for several reasons: 1) Biological Sensitivity: In spatial microenvironments, molecular changes often occur as subtle gradients rather than binary switches. A threshold of 0.26 allows the platform to capture these biologically significant transition zones. 2) Statistical Power: Our pixel-wise analysis treats each registered pixel as an individual observation. This high-density sampling provides substantial statistical power, ensuring that even smaller fold changes remain statistically robust (adjusted $p

	< 0.05$). 3) Alignment with Standards: This threshold closely mirrors the default settings of the widely used Seurat framework, ensuring that SMIntegration aligns with established community standards.<br>Specific changes made in the manuscript:<br>1. Added a new paragraph justifying the default thresholds and describing the role of PCA in guiding parameter selection. Revised text: "Five clustering methods are available (Figure 1C): Louvain (LV), LM, SLM [24], K-means after Principal Component Analysis (PCA), and K-means after Uniform Manifold Approximation and Projection (UMAP) [25,26]. To assist users in objective parameter selection, PCA is implemented as an initial visualization step to capture major spatial variation across many features. Users can leverage PCA plots to estimate the underlying complexity of the spatial domains and thus justify the selection of the number of clusters for subsequent analysis (Supplementary File 2). " (Page 7, Line 166).<br>2. Added a specific justification for the default log2FC threshold and highlighted its customizability. Revised text:" The threshold of 0.26 corresponds to a ~1.2-fold change, which is comparable to standard defaults in pipelines like Seurat. This allows for the detection of subtle molecular gradients and fine-scale heterogeneity in the tissue. Notably, this parameter is fully customizable, allowing users to apply more stringent filters depending on their specific biological questions and sample heterogeneity." (Page 8, Line 202).<br>Figure 1: We have updated the architecture diagram in Figure 1C to include PCA as part of the dimensionality reduction and clustering workflow.<br>Supplementary Material: We have added a new file (Supplementary File 2) demonstrating how PCA results can be used to inform the selection of cluster numbers for the mouse brain dataset, and updated Figure S5 to showcase the refined clustering interface which now integrates these assessment tools.<br>Software Interface: Updated the interface of the "Clustering Analysis and Cell Annotation" module to provide a step-by-step guide on how to interpret PCA and clustering results for parameter optimization.<br>Comment 3: " Some of the language, to my mind, over interprets the data. For example l169 "understanding of group specific regulatory mechanisms" I don't think you can infer mechanism directly from this type of exploratory analysis. Line 234 "discovery provides a novel molecular framework" - this is a potential hypothesis that can be tested, I don't think you can say it is a 'discovery'. Line 270 "strongly suggests the presence of a finely tuned inhibitory regulation" - this is just a correlation, and not 'strong' evidence in my opinion."<br>Response: We fully agree with the reviewer’s perspective. As an exploratory analysis tool, the primary goal of SMIntegration is to help researchers generate biologically plausible hypotheses and identify potential associations rather than providing direct proof of underlying biological mechanisms. We have carefully reviewed the manuscript and revised the language to better reflect the exploratory nature of the findings, softening our tone where necessary.<br>Specific changes made in the manuscript:<br>Regarding the description of regulatory mechanisms: We have replaced "mechanisms" with "patterns" to avoid over-interpretation. Revised text: "By enforcing the same node layout across groups (Figure S9), users can intuitively observe changes in gene-metabolite correlation patterns, facilitating identification of candidate group-specific gene–metabolite association patterns." (Page 8, Line 215).<br>Regarding the "molecular framework": We have changed "discovery" to "finding" and used more cautious language to describe the suggested framework. Revised text: "This finding offers a potential molecular basis for investigating region-specific mechanisms of motor control and reward, highlighting the unique value of integrated spatial multi-omics analysis." (Page 11, Line 287).<br>Regarding the relationship between Bex2 and spermidine: We have removed the word "strongly" and rephrased the sentence to emphasize that the observation is a potential relationship based on correlation. Revised text: "This phenomenon reveals that the correlation between the molecules Bex2 and spermidine is highly specific to cellular function: the significant negative correlation observed in the MO region, where decreased Bex2 expression coincides with increased spermidine levels, points to the potential presence of a finely tuned inhibitory relationship—potentially mediated by spermidine accumulation—targeting cell proliferation and apoptosis." (Page 12, Line 325).<br>In addition to these specific points, we have conducted a thorough review of the entire manuscript to ensure that our conclusions are appropriately grounded in the data as

exploratory findings. We appreciate the reviewer for helping us improve the scientific rigor of our presentation.

Comment 4: " Functional enrichment. For metabolomics this is not straightforward. Especially there will be uncertainty in the metabolite identities, as well as sparse mapping of metabolites to pathways. How are these aspects taken into account? At least the metabolite identification uncertainty should be acknowledge with a warning message. Is there a minimum number of metabolites/transcripts required to include a pathway? Mapping only a single metabolite to a pathway invalidates pathway testing. For enrichment (Fisher test), what background set is used? Use of the full set of e.g. KEGG compounds will greatly inflate p-values. What version of KEGG is used? What type of multiple testing is applied on for the pathway tests? Finally in the pathway enrichment plots, p-values are represented on a color scale 0-1 so it is hard to see any significance threshold. E.g. in figure 4D are any pathways significant? It would be clearer to visualise the log10 p-value here, with a specific mark for the cut-off. It also looks like the metabolites show higher rich factors than the transcripts. Rich factor is not defined, but if it relates to coverage of the pathway then this doesn't make sense - one would expect transcripts to have a higher coverage than metabolites."

Response: We sincerely thank the reviewer for this critical and insightful feedback regarding the challenges of functional enrichment in spatial metabolomics. We fully agree that the uncertainty in metabolite identification and the sparsity of pathway mapping present significant hurdles, and we have added a statement to emphasize that the interpretation of these data was conducted with appropriate caution.

Furthermore, we acknowledge the reviewer's concern that defining an appropriate background set for statistical testing (e.g., Fisher's exact test) is inherently difficult in MSI studies, where the detected metabolome represents only a fraction of the theoretical KEGG compound space (version 106.0). Using an overly broad background can indeed lead to inflated p-values and potentially misleading conclusions. Critically, we also recognize that the background sets for metabolites and transcripts are fundamentally different in size and composition. This inherent disparity makes the resulting p-values from separate enrichment analyses non-comparable, and their side-by-side visual presentation could be misleading.

Considering these valid concerns, we have refined our analytical strategy to prioritize biological robustness over statistical hypothesis testing based on uncertain assumptions. We have transitioned the module from a traditional "Enrichment Analysis" to a more transparent "Pathway Annotation Analysis" workflow. This approach focuses on reporting the count of annotated features (e.g., number of detected metabolites or transcripts mapped to a pathway) rather than comparing statistically derived p-values. We believe this provides a more intuitive and comparable measure of data coverage across different omics layers.

Specific revisions include:

1. Methodological Shift: We removed the Fisher's exact test and P-value calculations. The analysis now ranks pathways based on the number of annotated differential features.

2.Quality Control: We automatically exclude overly broad pathways (e.g., “Metabolic pathways”).

3.Visualization: We replaced the p-value bubble plot with a Ranked Annotation Dot Plot (Figure 4D), where the x-axis represents the annotation count, and shapes distinguish between metabolomic and transcriptomic contributions.

Changes in Manuscript:

1. Clarified the metabolite identification level and added a cautionary statement regarding data interpretation. Revised text: " It is important to note that this approach constitutes Level 3 annotation according to current metabolomics reporting standards. Therefore, all subsequent references to metabolites in the context of these data should be interpreted with caution, as they represent putatively annotated features rather than definitively identified compounds." (Page 9, Line 239)

2. Systematically updated key terminology throughout the manuscript to align with the revised analytical logic. We have implemented a systematic terminology update throughout the entire manuscript (including the Introduction, Results, Discussion, and Figure Legends) to align with the revised analytical approach. Specifically, we have replaced the following key terms:

(1) “functional enrichment” has been replaced with “functional annotation”.

(2) In the context of pathway analysis, terms such as “enriched” and “significantly enriched” have been replaced with “annotated”.

Powered by Editorial Manager® and ProduXion Manager® from Aries Systems Corporation

(3) “significant co-enrichment” and “co-enrichment” has been replaced with “co-annotation”.
3. Updated the method description for the revised functional analysis module. Revised text: "Functional Association and Annotation. This module integrates and interprets biological functions of DEGs and DAMs (Figure 1F). It performs pathway mapping (e.g., to KEGG pathways, version 106.0) and quantifies the number of DEGs and DAMs co-annotated to each pathway. Overly broad global pathways (e.g., "Metabolic pathways") are excluded. Pathway nodes are color-coded to indicate their up- or down-regulation, allowing users to visualize the spatial distribution of all annotated DEGs and DAMs for any given pathway (Figure S10)." (Page 9, Line 219)
4. Revised results descriptions to consistently use the updated annotation-based terminology. Revised text:
"Differential analysis identified 1484 differential genes (Figure 4B), corresponding to 212 pathways, and 193 differential mass features, corresponding to 86 pathways. There were 54 pathways shared by both differential genes and mass features (Figure 4C). " (Page 13, Line 344)
"Figure 4D shows the pathways containing these differential features. Genes and mass features highly expressed in the CA region were annotated to pathways such as Glutamatergic synaps..." (Page 13, Line 348)
"Pathways containing DEGs and DAMs highly expressed in the PAG region point to its core functions" (Page 13, Line 357)
5. Updated relevant figures and their legends to reflect the terminology and methodological changes: We have also updated Figure 4 and Supplementary Figures ((S10, S14) and its legend to reflect this change.
Comment 5: " Spatial pattern visualisation. It is very standard to use PCA to visualise spatial variation from a large number of features, but this doesn't seem to be available. Can the authors add this?"
Response: We sincerely thank the reviewer for this constructive suggestion. We agree that Principal Component Analysis (PCA) is an essential tool for visualizing spatial variation and capturing the major axes of variance in high-dimensional multi-omics data.
In response to your suggestion, we have integrated PCA into the "Pixel-level Spatial Clustering" module of SMIntegration. Specific changes made in the manuscript:
Methods Section (Pixel-level Spatial Clustering): We have updated the description to include PCA as a core method for both visualization and parameter guidance. Revised text: "Five clustering methods are available (Figure 1C): Louvain (LV), LM, SLM [24], K-means after Principal Component Analysis (PCA), and K-means after Uniform Manifold Approximation and Projection (UMAP) [25,26]. To assist users in objective parameter selection, PCA is implemented as an initial visualization step to capture major spatial variation across many features. Users can leverage PCA variance plots to estimate the underlying complexity of the spatial domains and thus justify the selection of the number of clusters for subsequent analysis (Supplementary File 2). " (Page 7, Line 166).
Figure 1: We have updated the architecture diagram in Figure 1C to include PCA as part of the dimensionality reduction and clustering workflow.
Supplementary Material: We have updated Figure S5 to showcase the refined clustering interface which now integrates these assessment tools.
We believe this addition significantly improves the analytical depth and user guidance of our platform. Thank you again for this valuable recommendation.

Comment 6: "Spatial pattern detection is done via SpaGene. Many readers won't be familiar with this, so please add a few sentences describing how the algorithm works. Other methods (e.g. Moran's I correlation) would also benefit from more detailed description, perhaps in supplementary or on the website. "
Response: We sincerely thank the reviewer for this suggestion. We agree that providing more technical details on the underlying algorithms is essential for a broader audience to understand and utilize the platform effectively.
In response, we have expanded the description of SpaGene and Moran’s I in the Methods section. Specifically, we have clarified that SpaGene is a graph-based, model-free method that identifies spatially variable features by comparing observed high-expression subnetworks against random permutations using Earth Mover’s Distance (EMD). We have also added a brief explanation of how Moran’s I is used to quantify the spatial consistency between different omics layers.
Specific changes made in the manuscript: " This module applies SpaGene [22] to

Powered by Editorial Manager® and ProduXion Manager® from Aries Systems Corporation

detect spatially variable (SV) features using a model-free, graph-based approach. Specifically, SpaGene constructs a k-nearest neighbor (k-NN) graph based on spatial coordinates and identifies high-expression subnetworks for each gene and metabolite. The spatial pattern strength is then quantified using Earth mover's distance (EMDg) which measures the distance between the observed high-expression distribution on the spatial graph and a null distribution derived from random permutations. Features with significant EMDg values are identified as SV features. Subsequently, non-negative matrix factorization (NMF) partitions these features into distinct spatial modules based on their pattern similarity (Figure 1B). To quantify cross-omics associations, the platform employs Moran's I [23], a measure of spatial autocorrelation, to evaluate the consistency of distribution patterns between identified gene and metabolite modules. Users can browse features within modules (Figure S4)." (Page 6, Line 152).

Comment 7: "Line 250: "1313 genes were highly expressed in the NA region, while 427 were highly expressed in the MO region (Figure 3D)" The figure shows different numbers: 1255 and 382."
Response: We sincerely apologize for the confusion caused by this error in our description. Upon re-examining our data, we realized that the numbers 1313 and 427 stated in the text were inadvertently calculated as the sum of both high-expression genes and high-intensity metabolites in the NA and MO regions, respectively. However, our intention was to specifically describe the number of high-expression genes in these two regions. The correct gene counts, as shown in Figure 3D, are 1255 and 382. We have now corrected the text to strictly refer to the high-expression gene counts. We appreciate the reviewer's meticulous review, which helped us ensure the consistency and accuracy of our report.
Specific changes made in the manuscript: "In terms of genes, 1255 genes were highly expressed in the NA region, while 382 were highly expressed in the MO region (Figure 3D)." (Page 11, Line 306)

Comment 8: "Line 241: "SMIntegration effectively addresses this challenge through its integrated cell type annotation and pixel registration" but you say that it doesn't perform coregistration of the two datasets? Clarify what you mean by 'registration'."
Response: We thank the reviewer for identifying this ambiguity. Our original use of the term "registration" in this context was intended to describe the process of matching and assigning labels between two already aligned modalities. We have made two major updates to address this:
1.Software Update: As noted in our response to Comment 1, we have now implemented a dedicated Spatial Registration module within the SMIntegration GUI (using the RNiftyReg package). This module allows users to perform the actual coregistration of the two datasets directly within the platform.
2.Clarified Terminology: In the revised manuscript, we have updated the description of the cell-type analysis workflow. We now use more precise language—"coordinate-based cell-type assignment" and "projecting labels"—to distinguish the transfer of biological annotations from the initial spatial alignment of the data slices.
Specific changes made in the manuscript: "SMIntegration effectively addresses this challenge through its integrated workflow of coordinate-based cell-type assignment and pixel registration. By projecting SingleR-identified labels from transcriptomics onto spatially aligned metabolomics pixels, the platform allows each pixel to be treated as a cell-type-specific sample (Figure 1D)." (Page 11, Line 293).

Comment 9: "When testing the app using the demo data, in step 4 "differential analysis" I was not able to input the cluster numbers or define the regions interactively as the app would not accept inputs. The cluster numbers just disappeared when clicking elsewhere on the app. For the interactive definition, the box "select feature to plot" was permanently blank. I could not perform the differential analysis."
Response:
We sincerely thank the reviewer for taking the time to test our application and for reporting this issue. We apologize for the inconvenience and confusion you experienced during the differential analysis step.
We have carefully reproduced the entire workflow and verified that all functions perform as expected under standard operating conditions. Having examined the issue you described, we suspect that the inability to select regions or input cluster numbers may have been caused by skipping the "Cell Annotation" step after completing the

Powered by Editorial Manager® and ProduXion Manager® from Aries Systems Corporation

"Clustering Analysis". The Differential Analysis module depends on the data generated during the Cell Annotation step (even if just using the demo or default annotation). Without this step, the downstream data structures required for ROI selection are not initialized.
To clarify the correct workflow, we have included a GIF below demonstrating the usage of the ROI selection module. You can also refer to the MP4 file in the attachments for supplementary information.

Furthermore, to enhance the user experience and prevent similar issues, we have implemented improved guidance and error-prevention mechanisms in the ROI selection module:
Explicit Step Numbering: The key prerequisite steps in the interface are now clearly labeled as Step 3.1 (Cluster Preprocessing), Step 3.2 (Cluster Analysis), and Step 3.3 (Cell Annotation) to visually outline the required workflow sequence.
Intelligent Input Validation: Within the ROI selection module, the system now performs real-time checks for the required data structures. If the necessary data is detected as missing or uninitialized, the interface dynamically displays a prompt: " Please complete 'Clustering Analysis' and 'Cell Annotation' before starting ROI Selection." This mechanism ensures users successfully finish all necessary upstream steps before attempting differential analysis.
These optimizations will ensure all users clearly understand the dependencies between modules and receive explicit guidance if an operation is blocked.
Supplementary Material: We have updated Figure S5 and S6 to showcase the changes in the prompts of the clustering analysis and cell annotation module.
Comment 10: "In my review of the github I found that the code is not commented at all. I was unable to find any R or Python script which contained comments. This is extremely poor practice in software development and needs to be rectified before publication. Other aspects such as unit tests and validation also did not appear to be present."
Response: We sincerely apologize for the lack of adequate documentation and formal validation in the initial version of our repository. We fully agree that for scientific software, transparency of code and verification of analytical logic are essential for reproducibility.
While SMIntegration is developed as a standalone Shiny application rather than a traditional R package, we recognize that the same standards of software rigor must apply. We have performed a comprehensive overhaul of the GitHub repository to meet these standards:
1. Systematic Code Commenting: We have reviewed and commented all R scripts (including UI, Server logic, and utility functions) and Python scripts. Every core function now includes detailed header comments explaining the input requirements, mathematical logic, and output formats.
2. Implementation of a Dual-Layer Validation Suite: To address the requirement for "unit tests and validation," we implemented a two-tiered validation framework:
2.1 Modular Unit Tests for the Analytical Pipeline (/validation_pipeline): Core modules (Preprocessing, Clustering, Differential Expression, Functional Association) were refactored into standalone, testable units. Using the downsampled demo dataset, we conducted targeted tests for each:
a.Preprocessing & Clustering: Validates data normalization, multi-omics integration, and Louvain clustering stability (01_test_preprocessing.R, 02_test_clustering.R).
b.Biologically-Contextualized Validation: A dedicated test (03_test_differential_analysis.R) automatically maps cell annotations and replicates the key Experimental (ACNT1/2) vs. Control (MOL1/2) group comparison from the manuscript.
c.Pathway Analysis: Confirms accurate mapping of differential features to KEGG pathways (04_test_functional_association.R).
2.2 Figure Reproduction for Output Verification (run_validation_figure.R): A regression test ensures reproducibility of final visual results. This script autonomously regenerates key manuscript figures (Figures 1-4) from archived intermediate data (RDS files), verifying visualization pipeline reliability.
For user convenience, master execution scripts (run_pipeline_validation.R and run_validation_figure.R) are provided in the root directory, enabling a single-command execution of the entire validation suite to verify both computational integrity and graphical output fidelity.
Specific changes made in the manuscript:

Software Implementation Section: Added a description of the validation protocols and code documentation efforts. Revised text: "SMIntegration is a web-based GUI implemented in R (v4.4.2) using Shinyproxy and Docker. To ensure the reliability of the integrated analytical workflow, we have implemented a systematic validation protocol. This includes (1) comprehensive inline documentation for all core analytical scripts on GitHub; and (2) a standalone, dual-layer validation suite comprising modular unit tests for core computational modules (e.g., spatial normalization, clustering, differential expression) and regression tests for figure reproducibility. "(Page 5, Line 112)
We believe these improvements significantly enhance the transparency and reliability of the SMIntegration platform.

References

[16] Chen A, Liao S, Cheng M, Ma K, Wu L, Lai Y, et al. Spatiotemporal transcriptomic atlas of mouse organogenesis using DNA nanoball-patterned arrays. Cell 2022; 185(10): 1777–1792.e21.
[17] Clayden J, Modat M, Presles B, Anthopoulos T, Daga P. RNiftyReg: Image registration using the "NiftyReg" library. R package version 2.8.4, 2024.
[18] Modat M, Cash DM, Daga P, Winston GP, Duncan JS, Ourselin S. Global image registration using a symmetric block-matching approach. J Med Imaging (Bellingham) 2014; 1: 024003.
[19] Modat M, Ridgway GR, Taylor ZA, Lehmann M, Barnes J, Hawkes DJ et al. Fast free-form deformation using graphics processing units. Comput Methods Programs Biomed 2010; 98: 278–284.
[20] Clayden JD, Maniega SM, Storkey AJ, King MD, Bastin ME, Clark CA. TractoR: Magnetic Resonance Imaging and Tractography with R. J. Stat. Soft. 2011; 44: 1–18.

#Reviewer 2

1.Summary
We sincerely appreciate the reviewer's thoughtful questions regarding the algorithmic transparency and technical implementation of SMIntegration. These inquiries have prompted us to significantly clarify the platform's internal logic, particularly concerning how biological associations are computationally derived and how multi-modal data with differing resolutions are integrated. In response to your comments, we have revised the manuscript to provide a more rigorous and detailed explanation of our methodologies. The key revisions are summarized below:
1)Clarified Discovery Logic for Cell-Specific Associations: We have expanded the "Results" and "Methods" sections to systematically explain the three-step computational process underlying our cell-type analysis: (1) Coordinate Transfer, where transcriptomics-derived labels are projected onto registered metabolomics pixels; (2) Parallel Differential Testing, which independently identifies markers for each omics layer; and (3) Functional Convergence, where the algorithm automatically flags pathway co-annotation (e.g., Slc6a11 and GABA in astrocytes). This ensures the "discovery" process is presented as a reproducible, algorithmic output rather than an opaque result.
2)Detailed "Align-Register-Fuse" Integration Framework: We have substantially elaborated on the technical algorithms used to handle resolution discrepancies. The revised manuscript now explicitly describes our high-to-low aggregation (binning) strategy for resolution matching, the implementation of the RNiftyReg algorithm for robust spatial registration, and the pixel-level matrix concatenation method used for joint clustering.
3)Enhanced Documentation: To support these clarifications, we have added five new technical references acknowledging the registration libraries and updated the Methods section to serve as a comprehensive technical guide for the integration workflow.
We believe these revisions provide the necessary technical depth to validate the platform's robustness. Detailed responses to each specific comment are provided below.
2. Point-by-point response to Comments and Suggestions for Authors
Comment 1: " The authors should explain more detailed for cell type analysis of their algorithm. How to figure out the association between astrocyte enriched GABA metabolism and Slc6a11?"
Response: We thank the reviewer for this constructive comment. To clarify how

SMIntegration "figures out" such associations from high-dimensional data, we have expanded the description in the Results section (Subheading: "Cell-Specific Metabolite Analysis").

The discovery of the association between Slc6a11 and GABA was achieved through a systematic three-layer filtering algorithm rather than manual selection:

Firstly, Spatial Coordinate Transfer. The platform first registers the Stereo-seq and AFADESI-MS data. Cell-type labels from the transcriptomics layer are then projected onto the metabolomics pixels based on their shared spatial coordinates. This defines the "non-telencephalon astrocyte (NA)" region at the pixel level.

Secondly, Parallel Differential Testing. The software executes independent Wilcoxon rank-sum tests for both genes and metabolites across these defined regions. Slc6a11 and GABA were independently identified as significant "markers" for the NA region.

Thirdly, Functional Convergence Screening. The "Functional Association" module takes the list of differentially expressed genes (DEGs) and differentially abundant metabolites (DAMs) and performs a co-enrichment analysis. The algorithm automatically identifies pathways where both a DEG and a DAM are present. In this case, the system flagged the GABAergic synapse pathway, thereby establishing the functional link between the transporter gene (Slc6a11) and the metabolite (GABA).

We have revised the manuscript to emphasize this automated discovery logic, ensuring that the process of "locating" these associations is transparent and reproducible.

Revised Text in Manuscript:

1. In "Methods - Cell Type Annotation": "Cell types are annotated for transcriptomics using SingleR [27] based on reference datasets (MouseRNAseqData, HumanPrimaryCellAtlasData). Since the transcriptomics and metabolomics modalities are spatially registered, SMIntegration transfers these cell-type labels to the corresponding metabolomics pixels based on their overlapping spatial coordinates. This allows the definition of cell-type-specific regions of interest (ROIs) for subsequent cross-modal differential analysis." (Page 7, Line 187)

2. In "Results - Cell-Specific Metabolite Analysis":" Deciphering gene expression and metabolite abundance changes within specific cell types is crucial for a deeper understanding of cellular function. However, spatial metabolomics data itself lacks direct cell type annotation capabilities. SMIntegration effectively addresses this challenge through its integrated workflow of coordinate-based cell-type assignment and pixel registration. By projecting SingleR-identified labels from transcriptomics onto spatially aligned metabolomics pixels, the platform allows each pixel to be treated as a cell-type-specific sample (Figure 1D). We demonstrate this by comparing two functionally distinct glial cell populations in the mouse brain: regions dominated by non-telencephalon astrocytes (NA) versus regions dominated by Mature oligodendrocytes (MO) (Figure 3A, Figure S7C). UMAP analysis showed clear differences in metabolite (Figure 3B) and gene (Figure 3C) expression between these two cell types.

To systematically pinpoint biological associations from the high-dimensional data, we applied a parallel discovery logic. Through differential screening, SMIntegration identified 103 differentials mass features, with 58 upregulated in NA and 45 upregulated in MO. In terms of genes, 1255 genes were highly expressed in the NA region, while 382 were highly expressed in the MO region (Figure 3D). To determine the functional synergy between these two lists, the platform's "Functional Association" module was utilized to perform automated co-annotation analysis. Notably, the gene Slc6a11 and the metabolite gamma-Aminobutyric acid (GABA) were algorithmically flagged as they both converged on the GABAergic synapse pathway (Figure S15A). Both were upregulated in the NA region (Figure S15B, C). Slc6a11 encodes a sodium-dependent transporter [36], and its absence can lead to GABA accumulation and an imbalance in neuronal excitability, affecting cognitive function [37]. These differential results are consistent with the cellular functions of astrocytes, which play a role in GABA synthesis and transmission." (Page 11, Line 291)

Comment 2: " Due to resolution difference, which algorithm has been utilized or developed for integration of spatial transcriptomics and spatial metabolomics in the web-tool?"

Response: We thank the reviewer for this important technical question. SMIntegration addresses the challenge of integrating multi-modal data with differing resolutions through a systematic "Align-Register-Fuse" computational framework:

Resolution Alignment (Aggregation/Binning): To harmonize the resolution difference (e.g., 500 nm ST vs. 50 μm SM), the platform utilizes a high-to-low aggregation algorithm. ST data are binned to match the specific grid resolution of the SM imaging

pixels, ensuring that both datasets share a consistent spatial unit.
Spatial Registration (RNiftyReg): As now detailed in the revised manuscript, we have integrated a dedicated registration module implemented via the RNiftyReg package. This algorithm supports both linear and non-linear transformations, allowing for precise alignment of tissue sections even when subtle morphological distortions exist between adjacent slices.
Multimodal Integration Algorithm (Pixel-level Fusion): Once aligned, the "integration" itself is achieved through pixel-level matrix concatenation. The platform treats each registered pixel as a unified observation, combining its transcriptomic and metabolomic profiles into a high-dimensional feature vector. This fused matrix then serves as the input for joint clustering algorithms, which identify shared spatial domains by considering both modalities simultaneously.
We have updated the Methods section to provide a more rigorous description of these algorithms and their implementation.
Revised Text in Manuscript:
1. On Resolution Alignment and Registration Algorithm: " SMIntegration input requires processed spatial metabolomics and transcriptomics data matrix. Since resolutions differ, higher-resolution data should be aggregated to match the lower (e.g., binning 500 nm transcriptomics by 100 to 50 μm metabolomics) [16]. To facilitate seamless integration, SMIntegration features a dedicated spatial registration module implemented using the RNiftyReg package [17], which provides an R interface to the NiftyReg library [18,19] and is part of the TractoR framework [20]. This module supports both linear (block-matching) and non-linear (free-form deformation) transformations to align the two modalities. Detailed tutorials for this registration module are provided in Supplementary File 1. Users also have the option to perform coregistration externally using the Python-based SpatialData ecosystem [21]; the aligned data can then be imported into SMIntegration in a compatible format for subsequent integrated analysis. Two input formats are supported: (1) text matrices containing feature name, spatial x/y coordinates, and values (Figure S2A); (2) Seurat objects with coordinates and abundance in designated slots (Figure S2B). Upload requirements are detailed on the help page." (Page 6, Line 131)
References: We have added four five new references [16-20] to acknowledge the developers of the registration library and the TractoR framework.
2. On Multimodal Integration (Fusion) Algorithm, section: Pixel-level Spatial Clustering: " Integrated data combines both modalities by pixel coordinates. This integration algorithm operates by concatenating the pre-processed, scaled feature matrices from both transcriptomics and metabolomics into a unified multimodal matrix. By treating each spatially registered pixel as a shared observation containing both gene and mass feature dimensions, the platform enables joint dimensionality reduction and clustering to uncover synchronized spatial domains. A Sankey diagram compares clustering concordance (Figure S5)." (Page 7, Line 179)
References
[16] Chen A, Liao S, Cheng M, Ma K, Wu L, Lai Y, et al. Spatiotemporal transcriptomic atlas of mouse organogenesis using DNA nanoball-patterned arrays. Cell 2022; 185(10): 1777–1792.e21.
[17] Clayden J, Modat M, Presles B, Anthopoulos T, Daga P. RNiftyReg: Image registration using the “NiftyReg” library. R package version 2.8.4, 2024.
[18] Modat M, Cash DM, Daga P, Winston GP, Duncan JS, Ourselin S. Global image registration using a symmetric block-matching approach. J Med Imaging (Bellingham) 2014; 1: 024003.
[19] Modat M, Ridgway GR, Taylor ZA, Lehmann M, Barnes J, Hawkes DJ et al. Fast free-form deformation using graphics processing units. Comput Methods Programs Biomed 2010; 98: 278–284.
[20] Clayden JD, Maniega SM, Storkey AJ, King MD, Bastin ME, Clark CA. TractoR: Magnetic Resonance Imaging and Tractography with R. J. Stat. Soft. 2011; 44: 1–18.

#Reviewer3

1.Summary

	We would like to express our sincere gratitude to the reviewers for their insightful comments and constructive suggestions. Their feedback has been invaluable in enhancing the scientific rigor, technical completeness, and clarity of our manuscript. In response to the reviewers' concerns, we have performed a comprehensive revision of both the SMIntegration platform and the manuscript. The key highlights of our revisions are summarized below:<br>1)Significant Functional Enhancement: To address the critical need for multi-modal alignment, we have implemented a dedicated Spatial Registration module within the SMIntegration GUI. This module utilizes the RNiftyReg framework to support both linear and non-linear transformations, providing a seamless transition from raw data to integrated analysis.<br>2)Enhanced Analytical Rigor & Transparency: Following the reviewers' suggestions, we have refined our preprocessing pipeline by explicitly decoupling normalization options (TIC and RMS) from data transformation. This modular approach ensures greater transparency and strictly aligns with mass spectrometry imaging (MSI) reporting standards. Furthermore, we have updated our terminology throughout the manuscript—replacing "metabolites" with "mass features"—to accurately reflect the Level 3 (tentative) identification confidence associated with MS1-based annotation.<br>3)Clarified Platform Scope and Positioning: We have carefully revised the manuscript to define SMIntegration as a specialized downstream integration platform. We have clarified that primary raw data processing (e.g., peak detection) remains external to the platform to preserve methodological flexibility and ensure computational scalability for concurrent users.<br>4)Detailed Methodological Justification: We have expanded our discussion on the statistical rationale behind our default differential analysis thresholds, emphasizing their suitability for capturing subtle molecular gradients within high-density spatial microenvironments.<br>We believe that these revisions significantly strengthen the utility of SMIntegration as a robust, user-friendly tool for the spatial omics community. Detailed responses to each of the reviewer's comments are provided below.<br><br>2. Point-by-point response to Comments and Suggestions for Authors<br>Comment 1: " Data preprocessing for spatial metabolomics data was done using the Cardinal package, and for spatial transcriptomics data using Seurat in this manuscript. This significantly reduces user-friendliness and requires substantial programming skills to use SMIntegration, making this tool difficult for many users. Please consider integrating the data preprocessing steps directly into SMIntegration."<br>Response: We sincerely appreciate the reviewer’s suggestion to enhance the user-friendliness of SMIntegration. We understand the value of an "all-in-one" solution; however, after careful consideration, we have decided to maintain SMIntegration’s focus as a specialized downstream integration platform rather than a primary raw data processor. Our reasoning is based on the following:<br>1)Methodological Flexibility and Scalability: Spatial metabolomics peak detection and spatial transcriptomics preprocessing are both computationally intensive and highly dependent on experimental design, instrumentation, and user-defined parameters. Integrating these steps directly into a shared cloud-based platform would substantially increase computational burden, affect scalability for concurrent users, and limit methodological flexibility. By allowing users to upload pre-processed features, we ensure they can utilize the most appropriate parameters specific to their raw data acquisition.<br>2)Platform Positioning: The core mission of SMIntegration is to bridge the gap between two omics modalities. Most researchers in this field already have established preferences and standardized pipelines for single-modality data quality control. SMIntegration acts as the next step in the workflow, focusing on cross-modal pattern recognition and co-localization.<br>We have revised the manuscript to better manage user expectations regarding the software's scope:<br>Abstract: Clarified that the platform provides a downstream integration workflow starting from the pre-processed spatial features: "Built with R/Shiny and deployed using Docker containerization, the platform provides a complete integrati...
**Additional Information:**	
**Question**	**Response**

Are you submitting this manuscript to a special series or article collection?	No
**Experimental design and statistics**<br><br>Full details of the experimental design and statistical methods used should be given in the Methods section, as detailed in our Minimum Standards Reporting Checklist. Information essential to interpreting the data presented should be made available in the figure legends.<br><br>Have you included all the information requested in your manuscript?	Yes
**Resources**<br><br>A description of all resources used, including antibodies, cell lines, animals and software tools, with enough information to allow them to be uniquely identified, should be included in the Methods section. Authors are strongly encouraged to cite Research Resource Identifiers (RRIDs) for antibodies, model organisms and tools, where possible.<br><br>Have you included the information requested as detailed in our Minimum Standards Reporting Checklist?	Yes
**Availability of data and materials**<br><br>All datasets and code on which the conclusions of the paper rely must be either included in your submission or deposited in publicly available repositories (where available and ethically appropriate), referencing such data using a unique identifier in the references and in the "Availability of Data and Materials" section of your manuscript.<br><br>Have you have met the above requirement as detailed in our Minimum	Yes

Standards Reporting Checklist?	
GigaScience has policies and guidelines in place for the use of generative AI-writing tools such as ChatGPT. If you have used such writing tools to assist with writing the manuscript this must be declared and cited in the text. Authors should not list AI-writing tools and other AI-assisted technologies as an author or co-author and should acknowledge that they are fully responsible for text generated or refined by AI-writing tools.<p><br><br>A summary of use (particularly in the introduction or among methods) needs to be included at the end of the paper, and the outputs should also be included as a supplementary file hosted in GigaDB or other open repositories. Please read our guidelines for more information. <p><br><br>By submitting to GigaScience, you are aware of the journal's AI-writing tools policy, and if you have declared use of such tools below, you have acknowledged this where appropriate in your manuscript and have made a summary of use and outputs available. <p><br><b>AI-assisted writing tools have been used in the preparation of this manuscript?	Yes

Manuscript
Click here to access/download;Manuscript;Manuscript-GIGA-D-25-00440 .docx

Title page

Article Title

SMIntegration: A Web Tool for Comprehensive Spatial Metabolomics and Transcriptomics Integrated Analysis and Visualization

Author(s) Names

Haoke Deng[1#], Xiaolian Ning[2#], Xun Lin[1], Liang Zong[1], Shanqiao Zheng[1], Yun Zhao[1], Jing Wang[1], Lingyun Chen[2], Jin Zi[1*], Zhanlong Mei[1*]

Author(s) Address Information

[1] *BGI, Shenzhen 518083, China*

[2] *BGI Research, Shenzhen 518083, China*

Symbol

[#] Equal contribution.

[*] Corresponding author(s).

E-mail: denghaoke@genomics.cn (Deng H), ningxiaolian@bgitechsolutions.com(Ning X) , linxun@genomics.cn (Lin X), zongliang@genomics.cn(Liang Z), zhengshanqiao@genomics.cn (Zheng S), zhaoyun@genomics.cn (Zhao Y), wangjing@genomics.cn(Wang J), chenlingyun@genomics.cn (Chen L), zij@genomics.cn (Zi J), meizhanlong@genomics.cn (Mei Z)

Running title: *Deng H et al / SMIntegration for Spatial Multi-omics*

[a]ORCID: Haoke Deng [0000-0002-1381-4818]

[b]ORCID: Xiaolian Ning [0009-0002-1213-4754]

[c]ORCID: Xun Lin [0009-0006-0169-6005]

[d]ORCID: Liang Zong [0000-0003-3751-4198]

[e]ORCID: Shanqiao Zheng [0009-0008-1685-8059]
[f]ORCID: Yun Zhao [0000-0001-7363-9487]
[g]ORCID: Jing Wang [0009-0009-8545-4573]
[h]ORCID: Lingyun Chen [0000-0001-5869-842X]
[i]ORCID: Jin Zi [0000-0002-1891-1393]
[j]ORCID: Zhanlong Mei [0000-0003-2203-2495]

Total word counts (from “Introduction” to “Conclusions” or “Materials and methods”): 3148

Total figures: 4

Total tables: 0

Total supplementary figures: 17

Total supplementary tables: 1

Total supplementary files: 20

Abstract page

Abstract

Current tools for spatial omics analysis often face challenges in performing integrated transcriptomics and metabolomics analysis, in-depth biological interpretation, and user-friendly operation. To address this, we developed SMIntegration, the first web-based graphical platform designed specifically for integrated spatial metabolomics and transcriptomics analysis. Built with R/Shiny and deployed using Docker containerization, the platform provides a complete integration workflow, starting from pre-processed spatial features through to functional annotation. Its core functions include: (1) automated and interactive spatial registration; (2) cross-modal spatial pattern recognition; (3) flexible differential analysis of genes and mass features based on clustering results, user-defined regions, or cell type annotations; and (4) group-specific gene-metabolite network construction and interactive visualization. Using adjacent mouse brain coronal sections (Stereo-seq transcriptomics and AFADESI-MS metabolomics) as an example, SMIntegration successfully identified both the periaqueductal gray and subcommissural organ, which were missed by single-modality clustering. Cell type analysis revealed an association between astrocyte-enriched GABA metabolism and *Slc6a11*, while a comparison between the cornu ammonis region and the midbrain periaqueductal gray dissected glutamatergic and endogenous cannabinoid signaling pathway modules. With a zero-code interface, SMIntegration enables a wide range of researchers to deeply explore gene-metabolite interaction mechanisms within microenvironments during development, homeostasis, and disease.

KEYWORDS: Spatial multi-omics; Spatial pattern analysis; Spatial differential analysis; Gene-metabolite co-localization; Differential expression network

Text body page

Introduction

Spatial omics technologies have revolutionized molecular biology by enabling localization of molecular information within tissue sections [1]. Spatial multi-omics, integrating transcriptomics, proteomics, and metabolomics, was recognized by Nature in 2022 as a “Technology to Watch” [2]. Integrating spatial transcriptomics and metabolomics is particularly important as it links gene expression (genotype) with metabolic products (phenotype), revealing mechanisms in development, disease, and therapy. Recent studies highlight this power: Sun et al. [3] profiled gastric cancer metabolic remodeling, while Vicari et al. [4] developed a protocol for simultaneous profiling on a single slice. Such approaches enable spatial clustering comparisons [3,5], identification of synergistic gene–metabolite modules [4,6], and cross-region interaction analyses [3,6]. These insights clarify how spatially defined genes regulate the metabolic microenvironment, advancing understanding of tissue development and disease.

Despite progress, current computational tools face three main limitations. First, modality compatibility is limited: platforms like SpatialGlue [7] and Giotto [8] mainly address transcriptomics–proteomics, and SpaTrio [9] links single-cell multi-omics with spatial transcriptomics. No standardized pipeline exists for spatial transcriptomics and spatial metabolomics integration. Second, biological interpretation remains shallow. SODB [10] allows data loading but not deep analysis; MIIT [11] supports registration but not interaction studies. Third, accessibility is poor: tools like MISO [12] and SOAPy [13] require programming expertise, while recent machine learning methods [14] are complex and lack GUIs. These gaps hinder research on how gene regulation shapes spatial metabolism, emphasizing the need for multimodal, analytical, and user-friendly platforms.

To address this, we developed SMIntegration, the first GUI platform for joint spatial metabolomics–transcriptomics analysis. It provides a streamlined downstram integration pipeline lowering the technical barriers. Core functions include: (1) Spatial

pattern analysis to identify co-varying features across omics; (2) Differential analysis based on clustering, user-defined ROIs, or cell types, with integrated functional annotation; and (3) Network analysis and visualization to construct differential expression genes (DEG)/ differential abundant mass features (DAM) correlation networks and explore spatial co-localization. Validated on mouse brain data, SMIntegration integrates both modalities, identifies fine brain structures, reveals astrocyte and oligodendrocyte networks, and uncovers mechanisms of synaptic plasticity and pain regulation, demonstrating strong potential for systems-level studies.

Methods

Software Implementation and Architecture

SMIntegration is a web-based GUI implemented in R (v4.4.2) using Shinyproxy and Docker. To ensure the reliability of the integrated analytical workflow, we have implemented a systematic validation protocol. This includes (1) comprehensive inline documentation for all core analytical scripts on GitHub; and (2) a standalone, dual-layer validation suite comprising modular unit tests for core computational modules (e.g., spatial normalization, clustering, differential expression) and regression tests for figure reproducibility. The complete computational workflow is archived in WorkflowHub [15]. The cloud platform (128 CPUs, 1000 GB RAM) is available online. Runtime benchmarks are in Table S1. For very large datasets, local deployment is recommended. Source code is publicly available, with documentation, tutorials, and example datasets accessible from the help interface (Figure S1).

Data Preparation and Spatial Registration

SMIntegration is specifically designed as a downstream integration platform. To maintain methodological flexibility and accommodate diverse experimental designs, primary raw data processing—such as peak detection for mass spectrometry imaging or initial analysis for spatial transcriptomics—is handled by specialized external tools. These steps are computationally intensive and highly dependent on instrumentation and

user-defined parameters; thus, performing them externally ensures scalability for concurrent users on our cloud-based platform and preserves methodological rigor.

SMIntegration input requires processed spatial metabolomics and transcriptomics data matrix. Since resolutions differ, higher-resolution data should be aggregated to match the lower (e.g., binning 500 nm transcriptomics by 100 to 50 μm metabolomics) [16]. To facilitate seamless integration, SMIntegration features a dedicated spatial registration module implemented using the RNiftyReg package [17], which provides an R interface to the NiftyReg library [18,19] and is part of the TractoR framework [20]. This module supports both linear (block-matching) and non-linear (free-form deformation) transformations to align the two modalities. Detailed tutorials for this registration module are provided in Supplementary File 1. Users also have the option to perform coregistration externally using the Python-based SpatialData ecosystem [21]; the aligned data can then be imported into SMIntegration in a compatible format for subsequent integrated analysis. Two input formats are supported: (1) text matrices containing feature name, spatial x/y coordinates, and values (Figure S2A); (2) Seurat objects with coordinates and abundance in designated slots (Figure S2B). Upload requirements are detailed on the help page.

Data Upload and Visualization

On the Overall Distribution Panel, users can upload datasets or use built-in test data (Figure S3). The system performs format checks, retains overlapping pixels, and generates abundance maps for both omics (**Figure 1A**).

Core Analysis Modules

Spatial Expression Pattern Recognition

This module applies SpaGene [22] to detect spatially variable (SV) features using a model-free, graph-based approach. Specifically, SpaGene constructs a k-nearest neighbor (k-NN) graph based on spatial coordinates and identifies high-expression subnetworks for each gene and metabolite. The spatial pattern strength is then quantified using Earth mover's distance (EMDg) which measures the distance between the observed high-expression distribution on the spatial graph and a null distribution derived from random permutations. Features with significant EMDg values are

identified as SV features. Subsequently, non-negative matrix factorization (NMF) partitions these features into distinct spatial modules based on their pattern similarity (**Figure 1B**). To quantify cross-omics associations, the platform employs Moran's I [23], a measure of spatial autocorrelation, to evaluate the consistency of distribution patterns between identified gene and metabolite modules. Users can browse features within modules (Figure S4).

Pixel-level Spatial Clustering

Five clustering methods are available (**Figure 1C**): Louvain (LV), LM, SLM [24], K-means after Principal Component Analysis (PCA), and K-means after Uniform Manifold Approximation and Projection (UMAP) [25,26]. To assist users in objective parameter selection, PCA is implemented as an initial visualization step to capture major spatial variation across many features. Users can leverage PCA variance plots to estimate the underlying complexity of the spatial domains and thus justify the selection of the number of clusters for subsequent analysis (Supplementary File 2). The Preprocessing pipeline follows a standardized workflow to handle the sparsity and technical variance of spatial data: (1) Normalization where users can choose Total Ion Current (TIC) or Root Mean Squared (RMS) normalization to account for pixel-wise technical variation; (2) Transformation, such as LogNormalize to stabilize variance; and (3) Scaling and variable feature selection (top 2000 genes/mass features). This modular design allows users to customize each step or skip them if the input data has been pre-processed. Integrated data combines both modalities by pixel coordinates. This integration algorithm operates by concatenating the pre-processed, scaled feature matrices from both transcriptomics and metabolomics into a unified multimodal matrix. By treating each spatially registered pixel as a shared observation containing both gene and mass feature dimensions, the platform enables joint dimensionality reduction and clustering to uncover synchronized spatial domains. A Sankey diagram compares clustering concordance (Figure S5).

Cell Type Annotation

Cell types are annotated for transcriptomics using SingleR [27] based on reference datasets (MouseRNAseqData, HumanPrimaryCellAtlasData). Since the

transcriptomics and metabolomics modalities are spatially registered, SMIntegration transfers these cell-type labels to the corresponding metabolomics pixels based on their overlapping spatial coordinates. (**Figure 1D**). This allows the definition of cell-type-specific regions of interest (ROIs) for subsequent cross-modal differential analysis. Users may also upload custom annotations (Figure S6).

Differential Analysis

This analysis consists of two steps: ROI selection and differential testing. ROIs can be defined in three ways (**Figure 1E**): Interactive Selection (manually drawing on metabolomics ion maps or transcriptomics expression maps, Figure S7A), Clustering-based Selection (Figure S7B), and Cell Type-based Selection (Figure S7C). After defining ROIs, users specify groups (e.g., Region A vs. Region B). Each pixel is treated as an independent sample, and Seurat's FindMarkers function [24] with a Wilcoxon rank-sum test identifies DEGs and DAMs. Results are Bonferroni-corrected [28], with default thresholds of $|\log2FC| > 0.26$ and adjusted $p < 0.05$. The threshold of 0.26 corresponds to a ~1.2-fold change, which is comparable to standard defaults in pipelines like Seurat. This allows for the detection of subtle molecular gradients and fine-scale heterogeneity in the tissue. Notably, this parameter is fully customizable, allowing users to apply more stringent filters depending on their specific biological questions and sample heterogeneity. In addition to univariate analysis, UMAP visualization highlights expression differences across ROIs, and users can view spatial distributions of identified DEGs or DAMs (Figure S8).

Group-specific Network Construction of Differential Features

This module reveals spatial co-expression relationships between DEGs and DAMs under specific biological conditions (Figure 1E). For each comparison group, differential genes and mass features are first selected, and Spearman correlation coefficients are calculated using pixel-level data. Pairs meeting adjusted p-value < 0.01 and $|r| > 0.6$ are retained as network edges. By enforcing the same node layout across groups (Figure S9), users can intuitively observe changes in gene–metabolite correlation patterns, facilitating identification of candidate group-specific gene–metabolite association patterns.

Functional Association and Annotation

This module integrates and interprets biological functions of DEGs and DAMs (**Figure 1F**). It performs pathway mapping (e.g., to KEGG pathways, version 106.0) and quantifies the number of DEGs and DAMs co-annotated to each pathway. Overly broad global pathways (e.g., "Metabolic pathways") are excluded. Pathway nodes are color-coded to indicate their up- or down-regulation, allowing users to visualize the spatial distribution of all annotated DEGs and DAMs for any given pathway (Figure S10).

Spatial Imaging Visualization

SMIntegration provides spatial visualization tools for exploring distribution patterns (**Figure 1G**). It supports single-feature imaging to generate spatial maps for any gene or metabolite, feature co-localization to display the top six positively and negatively correlated genes and mass features for a selected feature (Figure S11), and multi-feature visualization, where two to three features can be mapped to RGB channels to generate pseudo-color composite images (Figure S12). Together, these functions offer an intuitive means to explore spatial multi-omics relationships.

Example Data and Validation

SMIntegration includes datasets from adjacent coronal brain sections of a 7-week-old male mouse. Spatial metabolomics data were acquired by AFADESI-MS (50 μm resolution), processed with Cardinal [29]. Metabolite identification was performed using the SManalyst platform [30] based on monoisotopic mass matching yielding 13,707 pixels and 560 annotated mass features. It is important to note that this approach constitutes Level 3 annotation according to current metabolomics reporting standards [31]. Therefore, all subsequent references to mass features in the context of these data should be interpreted with caution, as they represent putatively annotated features rather than definitively identified compounds. Spatial transcriptomics data from Stereo-seq were binned to 50 μm, resulting in 14,605 pixels and 10,000 highly variable genes. The two modalities were registered using SpatialData (Figure S13), followed by KNN interpolation and filtering, yielding 14,530 valid pixels. A downsampled demo dataset (500 genes and 500 annotated mass features) is also provided, allowing users to quickly

test platform functions via the “Use demo data” option in the Overall Distribution Analysis panel.

Results and Discussion

Integrated Spatial Pattern Analysis Reveals Covarying Molecular Landscapes

SMIntegration identifies spatially consistent regions and molecular patterns by offering a variety of clustering algorithms and spatial pattern recognition methods. Joint clustering of integrated spatial metabolomics and transcriptomics data demonstrates improved spatial domain identification. We matched our mouse brain imaging data to the Allen Mouse Brain Reference Atlas [32] using DeepSlice [33] to identify the closest matching atlas plate, and then registered this atlas plate to our experimental images using QuickNII [34], and **Figure 2A** shows the brain structure of the mouse after registration. While separate spatial metabolomics clustering could only identify the periaqueductal gray (PAG) (**Figure 2B**), and separate transcriptomics clustering could only identify the subcommissural organ (SCO) (**Figure 2C**), the joint clustering of both spatial metabolomics and transcriptomics data accurately identified both regions simultaneously (**Figure 2D**). This highlights the potential of integrating different modalities to resolve fine spatial heterogeneity. The relationship between the clustering results of different modalities is visualized through a Sankey diagram (Figure S5), which demonstrates the unique information and degree of correspondence contributed by each omics layer to the spatial stratification.

In addition to spatial clustering, SMIntegration uses the SpaGene method to identify molecular expression patterns. **Figures 2E** and **2F** display the identified spatial expression patterns for genes and mass features, while **Figure 2G** shows the correlation between these spatial patterns. Although the overall clustering distributions exhibit similarities, the specific patterns differ. Some patterns appear to be complementary between modalities, such as metabolite pattern 2 and gene pattern 4 (r=-0.607). However, conserved cross-modal patterns were also found, such as metabolite pattern 3 and gene pattern 2 (r=0.565), both of which are enriched in the mid-brain region (Figures 2E, 2F). We performed functional annotation analysis on the conserved pattern

pair (Figure S14). The results revealed co-annotation of both patterns in key pathways such as synaptic vesicle cycle and neuroactive ligand-receptor interaction. This finding indicates that the co-localized module is deeply involved in the regulation of synaptic signaling in the midbrain region. It is noteworthy that the cAMP signaling pathway was also annotated. This pathway represents a classic intracellular signaling cascade that translates neurotransmitter receptor activation into changes in neuronal excitability and gene expression and is closely associated with synaptic plasticity [35].

The above analysis demonstrates the powerful capability of the SMIntegration platform in identifying spatially co-localized multi-omic modules. By recognizing coordinated spatial patterns of genes and mass features, it can be directly linked to functional synergy. This finding offers a potential molecular basis for investigating region-specific mechanisms of motor control and reward, highlighting the unique value of integrated spatial multi-omics analysis.

Cell-Specific Metabolite Analysis

Deciphering gene expression and metabolite abundance changes within specific cell types is crucial for a deeper understanding of cellular function. However, spatial metabolomics data itself lacks direct cell type annotation capabilities. SMIntegration effectively addresses this challenge through its integrated workflow of coordinate-based cell-type assignment and pixel registration. By projecting SingleR-identified labels from transcriptomics onto spatially aligned metabolomics pixels, the platform allows each pixel to be treated as a cell-type-specific sample (Figure 1D). We demonstrate this by comparing two functionally distinct glial cell populations in the mouse brain: regions dominated by non-telencephalon astrocytes (NA) versus regions dominated by Mature oligodendrocytes (MO) (**Figure 3A**, Figure S7C). UMAP analysis showed clear differences in metabolite (**Figure 3B**) and gene (**Figure 3C**) expression between these two cell types.

To systematically pinpoint biological associations from the high-dimensional data, we applied a parallel discovery logic. Through differential screening, SMIntegration identified 103 differentials mass features, with 58 upregulated in NA and 45 upregulated in MO. In terms of genes, 1255 genes were highly expressed in the NA

region, while 382 were highly expressed in the MO region (**Figure 3D**). To determine the functional synergy between these two lists, the platform’s "Functional Association" module was utilized to perform automated co-annotation analysis. Notably, the gene *Slc6a11* and the metabolite gamma-Aminobutyric acid (GABA) were algorithmically flagged as they both converged on the GABAergic synapse pathway (Figure S15A). Both were upregulated in the NA region (Figure S15B, C). *Slc6a11* encodes a sodium-dependent transporter [36], and its absence can lead to GABA accumulation and an imbalance in neuronal excitability, affecting cognitive function [37]. These differential results are consistent with the cellular functions of astrocytes, which play a role in GABA synthesis and transmission.

Cell-type-specific metabolic regulatory networks provide a new perspective for dissecting the mechanisms of metabolic-gene synergistic interactions. We constructed correlation networks for these differential features in the NA and MO regions separately. In the NA region, no significant correlation was observed between *Bex2* and spermidine (Figure 3E); whereas in the MO region, decreased expression of the *Bex2* gene and upregulation of spermidine were detected, showing a negative correlation between them (Figure 3F). *Bex2* has anti-apoptotic and pro-proliferative properties [38], while the effect of spermidine on the immune system is dose-dependent, with higher doses being anti-inflammatory and lower doses enhancing cytotoxic immune function [39]. This phenomenon reveals that the correlation between the molecules *Bex2* and spermidine is highly specific to cellular function: the significant negative correlation observed in the MO region, where decreased *Bex2* expression coincides with increased spermidine levels, points to the potential presence of a finely tuned inhibitory relationship—potentially mediated by spermidine accumulation—targeting cell proliferation and apoptosis. In contrast, the lack of correlation in the NA region may indicate a functional decoupling between the two. Such specificity in interaction is likely closely related to the immune microenvironment or neural activity demands of the cell population. These results reflect the heterogeneity of transcriptomics and metabolomics at the cell type level, validating the platform's utility in precisely dissecting cell-specific molecular networks.

Differential Expression Between Brain Regions Reveals Functional Insights

Beyond supporting differential analysis based on cell type annotations, SMIntegration's flexible interactive selection feature (Figure 1E) also allows researchers to directly select any region of interest to define comparison groups. We used the comparison between the cornu ammonis (CA) region and the mid-brain periaqueductal gray (PAG) region in a mouse brain coronal section (Figure S16, **Figure 4A**) as an example to demonstrate SMIntegration's flexible differential analysis to reveal regulatory networks in different brain microenvironments. Differential analysis identified 1484 differential genes (**Figure 4B**), corresponding to 212 pathways, and 193 differential mass features, corresponding to 86 pathways. There were 54 pathways shared by both differential genes and mass features (**Figure 4C**).

Figure 4D shows the pathways containing these differential features. Genes and mass features highly expressed in the CA region were annotated to pathways such as Glutamatergic synapse, Neuroactive ligand-receptor interaction, and Long-term potentiation/depression. In particular, in the activated Glutamatergic synapse pathway (Figure S17A), L-Glutamic acid (Figure S17B) and *Grin2a* (Figure S17C) were upregulated. Glutamate is the main excitatory neurotransmitter in the hippocampus, and its metabolic flux directly affects synaptic plasticity. The gene *Grin2a* encodes the NMDAR, an ionotropic glutamate receptor. The synergistic action of these pathways forms the molecular basis of hippocampus-dependent learning and memory [40].

Pathways containing DEGs and DAMs highly expressed in the PAG region point to its core functions: pain modulation and defensive responses. In the addiction-related neuroadaptations pathway (Figure 4E), both the metabolite gamma-Aminobutyric acid (GABA) and the gene *Slc32a1* were significantly upregulated (**Figure 4F**). *Slc32a1* encodes the vesicular transporter VGAT, which loads GABA into synaptic vesicles, and GABA is an inhibitory tonic [41]. Studies have shown that the analgesic effect of exogenous cannabinoids (e.g., Δ9-THC) in the PAG is achieved by activating CB1 receptors to modulate vesicular release patterns and reduce the probability of GABA release [41,42]. Furthermore, multi-feature imaging and spatial co-expression analysis

also demonstrated the consistent high expression of *Slc32a1* and GABA in the mid-brain region, predominantly the PAG (**Figure 4G**).

Conclusion

SMIntegration is the first zero-code platform for integrated spatial metabolomics and transcriptomics, unifying spatial pattern recognition, differential analysis, network construction, and functional annotation. It enables intuitive exploration of gene–metabolite interactions and spatial heterogeneity, as demonstrated in the mouse brain by revealing region- and cell type–specific networks and key processes such as GABA/Glutamate balance and cannabinoid signaling. Future extensions will include additional omics and machine learning–based modules, further advancing spatial multi-omics research in both fundamental and translational biology.

Institutional Review Board Statement

The animal study protocol was approved by the Institutional Review Board of BGI (protocol code BGI-IRB A25004 and date of approval 21 February 2025)

Availability of Source Code and Requirements

Project name: SMIntegration

Project homepage: https://github.com/mzlab-research/SMIntegration

Operating system(s): Platform independent (MacOS, Linux, Windows)

Programming language: R

Other requirements: None

License: MIT license

RRID: SCR_027925

biotoolsID: smintegration

Web Server: https://metax.genomics.cn/app/SMIntegration

Docker Image: https://hub.docker.com/r/mzlabresearch/smintegration

Data Availability

All resources described in this study are publicly available. The raw spatial metabolomics and spatial transcriptomics data from mouse brain tissue, as well as the derived processed peak intensity tables, have been deposited in the China National Center for Bioinformation (CNCB) OMIX database. The spatial metabolomics and transcriptomics data are accessible under accession number OMIX011674 [43]. The processed data is available in GitHub repository [44].

Authors' contributions

Author Contributions (CRediT):

HKD: Conceptualization, Methodology, Software, Writing – Original Draft (Methods and Results)

XLN: Investigation, Formal Analysis, Writing – Original Draft (Introduction)

SQZ: Methodology, Data Curation, Software

LZ: Investigation, Resources

JW: Investigation, Resources

YZ: Investigation, Resources

CLY: Investigation, Resources

JZ: Supervision, Conceptualization

ZLM: Conceptualization, Writing – Review & Editing

All authors: Review & Approval of Final Manuscript

Competing interests

Haoke Deng, Xun Lin, Liang Zong, Shanqiao Zheng, Yun Zhao, Jing Wang, Jin Zi and Zhanlong Mei are employees of BGI Genomics. Xiaolian Ning and Lingyun Chen are employees of BGI Research. This paper reflects the views of the scientists, not the company.

Acknowledgments

This research was funded by the National Key R&D Program of China, grant number 2021YFA0805100, and the Sustainable Development Program of Shenzhen Science and Technology Major Program, grant number KCXFZ20240903093925033. During the preparation of this manuscript, the authors used Gemini 2.5 Flash (Experimental) to polish the language. The authors have reviewed and edited the generated content and take full responsibility for the content of this publication.

References

[1] Ståhl PL, Salmén F, Vickovic S, Lundmark A, Navarro JF, Magnusson J, et al. Visualization and analysis of gene expression in tissue sections by spatial transcriptomics. Science 2016; 353: 78−82.

[2] Eisenstein M. Seven technologies to watch in 2022. Nature 2022; 601: 658−61.

[3] Sun C, Wang A, Zhou Y, Chen P, Wang X, Huang J, et al. Spatially resolved multi-omics highlights cell-specific metabolic remodeling and interactions in gastric cancer. Nat Commun 2023; 14: 2692.

[4] Vicari M, Mirzazadeh R, Nilsson A, Shariatgorji R, Bjärterot P, Larsson L, et al. Spatial multimodal analysis of transcriptomes and metabolomes in tissues. Nat Biotechnol 2024; 42:1046–50.

[5] Ravi VM, Will P, Kueckelhaus J, Sun N, Joseph K, Salié H, et al. Spatially resolved multi-omics deciphers bidirectional tumor-host interdependence in glioblastoma. Cancer Cell 2022; 40: 639–55.e13.

[6] Zheng P, Zhang N, Ren D, Yu C, Zhao B, Zhang Y. Integrated spatial transcriptome and metabolism study reveals metabolic heterogeneity in human injured brain. Cell Rep Med 2023; 4: 101057.

[7] Long Y, Ang KS, Sethi R, Liao S, Heng Y, van Olst L, et al. Deciphering spatial domains from spatial multi-omics with SpatialGlue. Nat Methods 2024; 21: 1658–67.

[8] Dries R, Zhu Q, Dong R, Eng CHL, Li H, Liu K, et al. Giotto: a toolbox for integrative analysis and visualization of spatial expression data. Genome Biol 2021; 22: 78.

[9] Yang P, Jin L, Liao J, Jin K, Shao X, Li C, et al. Revealing spatial multimodal heterogeneity in tissues with SpaTrio. Cell Genomics 2023; 3: 100446.

[10] Yuan Z, Pan W, Zhao X, Zhao F, Xu Z, Li X, et al. SODB facilitates comprehensive exploration of spatial omics data. Nat Methods 2023; 20: 387–399.

[11] Wess M, Andersen MK, Midtbust E, Guillem JCC, Viset T, Størkersen Ø, et al. Spatial integration of multi-omics data from serial sections using the novel Multi-Omics Imaging Integration Toolset. Gigascience 2025; 14: giaf035.

[12] Coleman K, Schroeder A, Loth M, Zhang D, Park JH, Sung JY, et al. Resolving tissue complexity by multimodal spatial omics modeling with MISO. Nat Methods 2025; 22: 530–8.

[13] Wang H, Li J, Jing S, Lin P, Qiu Y, Yan X, et al. SOAPy: a Python package to dissect spatial architecture, dynamics, and communication. Genome Biol 2025; 26: 80.

[14] Dexter A, Thomas SA, Steven RT, Robinson KN, Taylor AJ, Elia EA, et al. A New Approach to Large Multiomics Data Integration. Anal Chem 2025; Advance online publication.

[15] Deng H, Ning X, Lin X, Zong L, Zheng S, Zhao Y et al. SMIntegration Workflow: Spatial Metabolomics and Transcriptomics Analysis. WorkflowHub 2026; https://doi.org/10.48546/WORKFLOWHUB.WORKFLOW.2074.5

[16] Chen A, Liao S, Cheng M, Ma K, Wu L, Lai Y, et al. Spatiotemporal transcriptomic atlas of mouse organogenesis using DNA nanoball-patterned arrays. Cell 2022; 185(10): 1777–1792.e21.

[17] Clayden J, Modat M, Presles B, Anthopoulos T, Daga P. RNiftyReg: Image registration using the “NiftyReg” library. R package version 2.8.4, 2024.

[18] Modat M, Cash DM, Daga P, Winston GP, Duncan JS, Ourselin S. Global image registration using a symmetric block-matching approach. J Med Imaging (Bellingham) 2014; 1: 024003.

[19] Modat M, Ridgway GR, Taylor ZA, Lehmann M, Barnes J, Hawkes DJ et al. Fast free-form deformation using graphics processing units. Comput Methods Programs Biomed 2010; 98: 278–284.

[20] Clayden JD, Maniega SM, Storkey AJ, King MD, Bastin ME, Clark CA. TractoR: Magnetic Resonance Imaging and Tractography with R. J. Stat. Soft. 2011; 44: 1–18.

[21] Marconato L, Palla G, Yamauchi KA, Virshup I, Heidari E, Treis T, et al. SpatialData: an open and universal data framework for spatial omics. Nat Methods 2025; 22: 58–62.

[22] Liu Q, Hsu CY, Shyr Y. Scalable and model-free detection of spatial patterns and colocalization. Genome Res 2022; 32: 1736–45.

[23] Bivand R, Müller WG, Reder M. Power calculations for global and local Moran's I. Comput Stat Data Anal 2009; 53: 2859–72.

[24] Stuart T, Butler A, Hoffman P, Hafemeister C, Papalexi E, Mauck WM, et al. Comprehensive Integration of Single-Cell Data. Cell 2019; 177: 1888–902.e21.

[25] Do VH, Canzar S. A generalization of t-SNE and UMAP to single-cell multimodal omics. Genome Biol 2021; 22: 130.

[26] Steinley D. K-means clustering: A half-century synthesis. Br J Math Stat Psychol 2006; 59: 1–34.

[27] Aran D, Looney AP, Liu L, Wu E, Fong V, Hsu A, et al. Reference-based analysis of lung single-cell sequencing reveals a transitional profibrotic macrophage. Nat Immunol 2019; 20: 163–172.

[28] Satija R, Farrell JA, Gennert D, Schier AF, Regev A. Spatial reconstruction of single-cell gene expression data. Nat Biotechnol 2015; 33: 495–502.

[29] Bemis KD, Harry A, Eberlin LS, Ferreira C, Van De Ven SM, Mallick P, et al. Cardinal: An R package for statistical analysis of mass spectrometry-based imaging experiments. Bioinformatics 2015; 31: 2418–20.

[30] Mei Z, Ning X, Deng H, Chen L, Zhao Y, Jin Z. SMAnalyst: A Web Server for Spatial Metabolomic Data Analysis and Annotation. Preprints 2025; 2025091621.

[31] Schymanski EL, Jeon J, Gulde R, Fenner K, Ruff M, Singer HP, et al. Identifying Small Molecules via High Resolution Mass Spectrometry: Communicating Confidence. Environ Sci Technol 2014; 48:2097–8.

[32] Wang Q, Ding SL, Li Y, Royall J, Feng D, Lesnar P, et al. The Allen Mouse Brain Common Coordinate Framework: A 3D Reference Atlas. Cell 2020; 181: 936–53.e20.

[33] Carey H, Pegios M, Martin L, Saleeba C, Turner AJ, Everett NA, et al. DeepSlice: rapid fully automatic registration of mouse brain imaging to a volumetric atlas. Nat Commun 2023; 14: 5884.

[34] Puchades MA, Csucs G, Ledergerber D, Leergaard TB, Bjaalie JG. Spatial registration of serial microscopic brain images to three-dimensional reference atlases with the QuickNII tool. PLoS One 2019; 14: e0216796.

[35] Kandel ER. The molecular biology of memory: CAMP, PKA, CRE, CREB-1, CREB-2, and CPEB. Mol Brain 2012; 5:14.

[36] Pramod AB, Foster J, Carvelli L, Henry LK. SLC6 transporters: Structure, function, regulation, disease association and therapeutics. Mol Aspects Med 2013; 34: 197–219.

[37] Dikow N, Maas B, Karch S, Granzow M, Janssen JWG, Jauch A, et al. 3p25.3 microdeletion of GABA transporters SLC6A1 and SLC6A11 results in intellectual disability, epilepsy and stereotypic behavior. Am J Med Genet A 2014; 164A: 3061–8.

[38] Mu N, Wang Y, Li X, Du Z, Wu Y, Su M, et al. Crotonylated *BEX2* interacts with NDP52 and enhances mitophagy to modulate chemotherapeutic agent-induced apoptosis in non-small-cell lung cancer cells. Cell Death Dis 2023; 14: 645.

[39] Chamoto K, Zhang B, Tajima M, Honjo T, Fagarasan S. Spermidine – an old molecule with a new age-defying immune function. Trends Cell Biol 2024; 34: 363–70.

[40] McNair LM, Andersen JV, Waagepetersen HS. Stable isotope tracing reveals disturbed cellular energy and glutamate metabolism in hippocampal slices of aged male mice. Neurochem Int 2023; 171: 105626.

[41] Keay KA, Bandler R. Parallel circuits mediating distinct emotional coping reactions to different types of stress. Neurosci Biobehav Rev 2001; 25: 669–78.

[42] Finn DP, Jhaveri MD, Beckett SRG, Roe CH, Kendall DA, Marsden CA, et al. Effects of direct periaqueductal grey administration of a cannabinoid receptor agonist on nociceptive and aversive responses in rats. Neuropharmacology 2003; 45: 594–604.

[43] CNCB OMIX data. https://ngdc.cncb.ac.cn/omix/release/OMIX011674. Accessed on 1 September 2025

[44] SMIntegration. GitHub Repository. https://github.com/mzlab-research/SMIntegration/tree/main/spatialdata. Accessed on 1 September 2025.

Figure legends

Figure 1 Overall architecture of SMIntegration

A. Data Upload and Registration. **B.** Spatial pattern analysis, identifying features with specific spatial patterns. **C.** Clustering analysis. **D.** Cell type annotation analysis. **E.** Differential analysis, allowing the selection of corresponding differential features through three different methods. **F.** Functional association analysis of differential features. **G.** Imaging visualization.

Figure 2 Integrated multi-omics clustering identifies conserved spatial domains and correlated molecular patterns

A. Schematic of mouse brain structure (atlas from ABA_Mouse_CCFv3_2017_25um.cutlas). **B.** Spatial metabolomics clustering. **C.** Spatial transcriptomics clustering. **D.** Integrated two-omics clustering map. **E.** Spatial pattern identification from spatial transcriptomics data. **F.** Spatial pattern identification from spatial metabolomics data. **G.** Spatial correlation between spatial metabolomics and spatial transcriptomics patterns. (The spatial pattern maps in B-F include brain region outlines for easier identification.)

Figure 3 Cell type-based differential analysis reveals cell-enriched mass features and genes.

A. Group comparison selection based on cell types, with the experimental group being non-telencephalon astrocytes (NA) and the control group being mature oligodendrocytes (MO). **B.** UMAP analysis of genes in the MO and NA regions. **C.** UMAP analysis of mass features in the MO and NA regions. **D.** Number of differentially expressed genes and mass features in MO and NA. **E.** Correlation network between differential mass features and genes in the NA region. **F.** Correlation network between differential mass features and genes in the MO region.

Figure 4 Interactive spatial selection unveils region-specific multi-omics functional pathways

A. Group comparison selection based on brain regions, with the experimental group as cornu ammonis (CA) and the control group as periaqueductal gray (PAG). **B.** Number

of differentially expressed genes and mass features in the CA and PAG regions. **C.** Number of annotated pathways for differential genes and mass features in the CA and PAG regions. **D.** Pathway annotation for differential features in the CA and PAG regions. **E.** Pathway Retrograde endocannabinoid signaling annotated with mass features and genes in the PAG region. **F.** Abundance distribution maps of enriched genes and mass features in the PAG region. **G.** Spatial co-imaging (left) and co-expression (right) of *Slc32a1* and GABA.

Supplementary material

Supplementary File 1 Spatial metabolomics and transcriptomics data registration tutorial

Supplementary File 2 Clustering Parameter Selection Tutorial: Using PCA for Data-Driven Clustering in SMIntegration

Table S1 Execution time and memory consumption across datasets of varying sizes

Figure S1 Screenshot of the SMIntegration tutorial interface

Figure S2 Schematic diagram of data input formats.

A. Text Matrix Format: Requires metabolomics/transcriptomics data to be submitted as a "feature-pixel" matrix, with each column containing the metabolite/gene name, spatial coordinates (x/y), and feature value. **B.** Seurat Object Format: Requires both spatial metabolomics and transcriptomics data to share identical structures. The feature-pixel matrix is stored in the Spatial$counts slot (rows represent features and columns represent pixels), while spatial coordinates are stored in the meta.data slot.

Figure S3 Screenshot of the data upload interface

Figure S4 Screenshot of the spatial pattern analysis interface

Figure S5 Screenshot of the spatial clustering interface

Figure S6 Cell type annotation

A. Screenshot of the cell type annotation interface. **B.** Requirements for user-defined annotation file format: Must be a text matrix with three columns: spatial x/y coordinates and cell type.

Figure S7 Screenshot of the differential analysis region selection interface

A. Use the lasso tool to select ROI, then assign it as the experimental or control group. **B.** Select clustering classes, then assign them as the experimental or control group. **C.** Select cell classes, then assign them as the experimental or control group.

Figure S8 Screenshot of the differential analysis interface

Figure S9 Screenshot of the group-specific network interface

Figure S10 Screenshot of the functional association analysis interface

Figure S11 Screenshot of the single-feature visualization interface

Figure S12 Screenshot of the multi-feature visualization interface

Figure S13 Mouse brain data registration workflow

A. Total abundance imaging of spatial transcriptomics and spatial metabolomics data before registration.

B. Total abundance imaging of spatial transcriptomics and spatial metabolomics data after registration.

Figure S14 Annotated pathways in the conserved metabolite pattern 3 and gene expression pattern 2

Figure S15 Annotation of enriched features in non-telencephalic astrocytes within the GABAergic synapse pathway

A. Molecular annotation in the GABAergic synapse pathway. **B.** The spatial distribution of Gamma-aminobutyric acid (GABA) abundance. **C.** The spatial distribution of *Slc6a11* expression level.

Figure S16 Schematic diagram of manual selection for CA vs PAG

Figure S17 Annotation of enriched features in CA region within the glutamatergic synapse pathway

A. Molecular annotation in the glutamatergic synapse pathway. **B.** The spatial distribution of L-Glutamic acid abundance. **C.** The spatial distribution of *Grin2a* expression level.

Figure accessibility and alt text

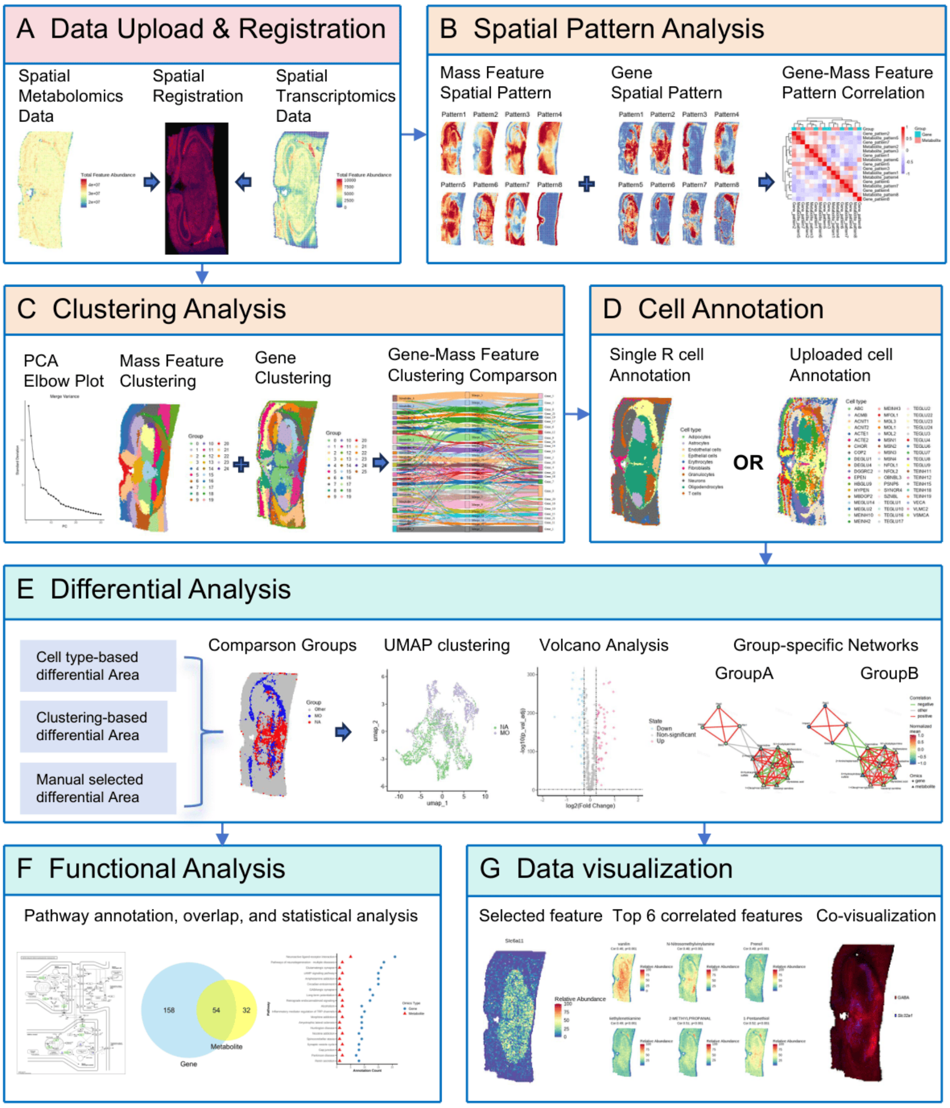

Figure 1 Overall architecture of SMIntegration

A. Data Upload and Registration. **B.** Spatial pattern analysis, identifying features with specific spatial patterns. **C.** Clustering analysis. **D.** Cell type annotation analysis. **E.** Differential analysis, allowing the selection of corresponding differential features through three different methods. **F.** Functional association analysis of differential features. **G.** Imaging visualization.

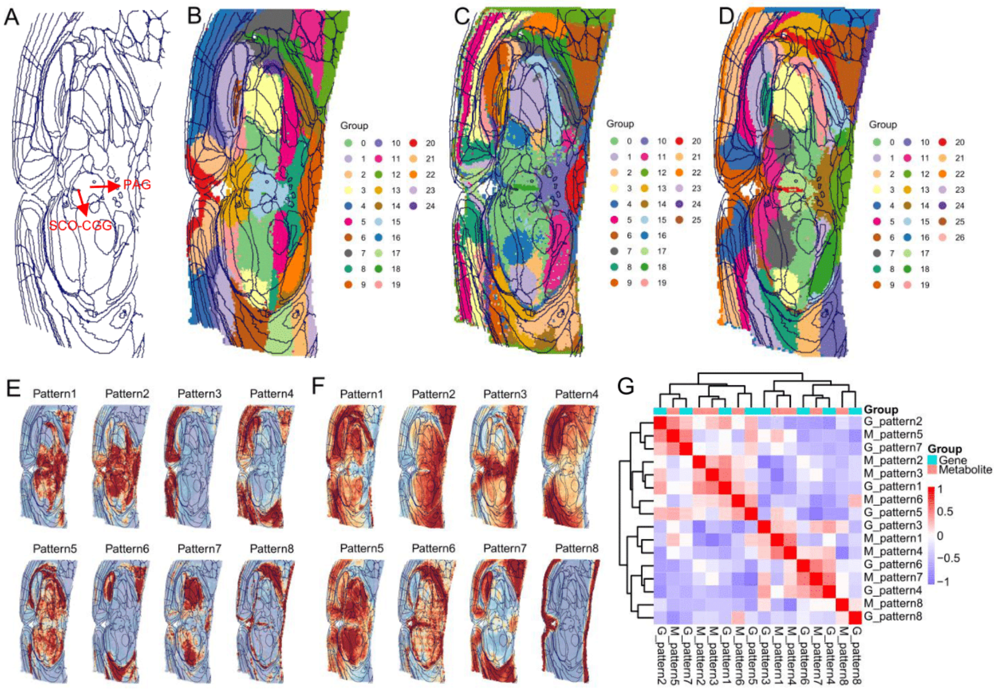

Figure 2 Integrated multi-omics clustering identifies conserved spatial domains and correlated molecular patterns

A. Schematic of mouse brain structure (atlas from ABA_Mouse_CCFv3_2017_25um.cutlas). **B.** Spatial metabolomics clustering. **C.** Spatial transcriptomics clustering. **D.** Integrated two-omics clustering map. **E.** Spatial pattern identification from spatial transcriptomics data. **F.** Spatial pattern identification from spatial metabolomics data. **G.** Spatial correlation between spatial metabolomics and spatial transcriptomics patterns. (The spatial pattern maps in B-F include brain region outlines for easier identification.)

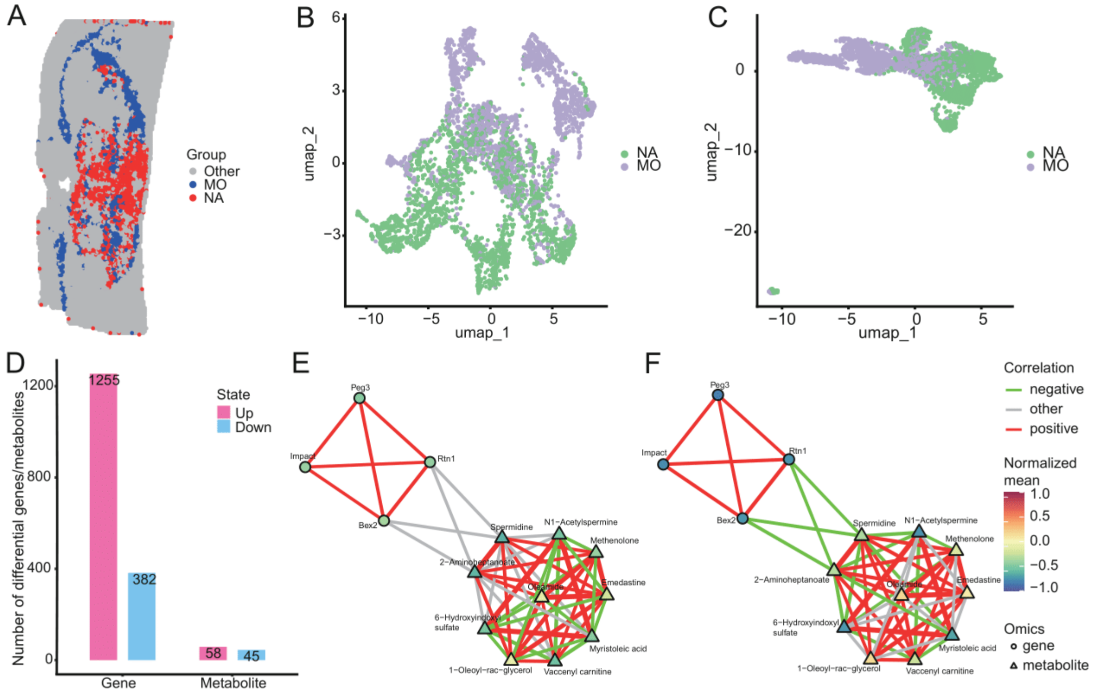

Figure 3 Cell type-based differential analysis reveals cell-enriched mass features and genes.

A. Group comparison selection based on cell types, with the experimental group being non-telencephalon astrocytes (NA) and the control group being mature oligodendrocytes (MO). **B.** UMAP analysis of mass features in the MO and NA regions. **C.** UMAP analysis of genes in the MO and NA regions. **D.** Number of differentially expressed genes and mass features in MO and NA. **E.** Correlation network between differential mass features and genes in the NA region. **F.** Correlation network between differential mass features and genes in the MO region.

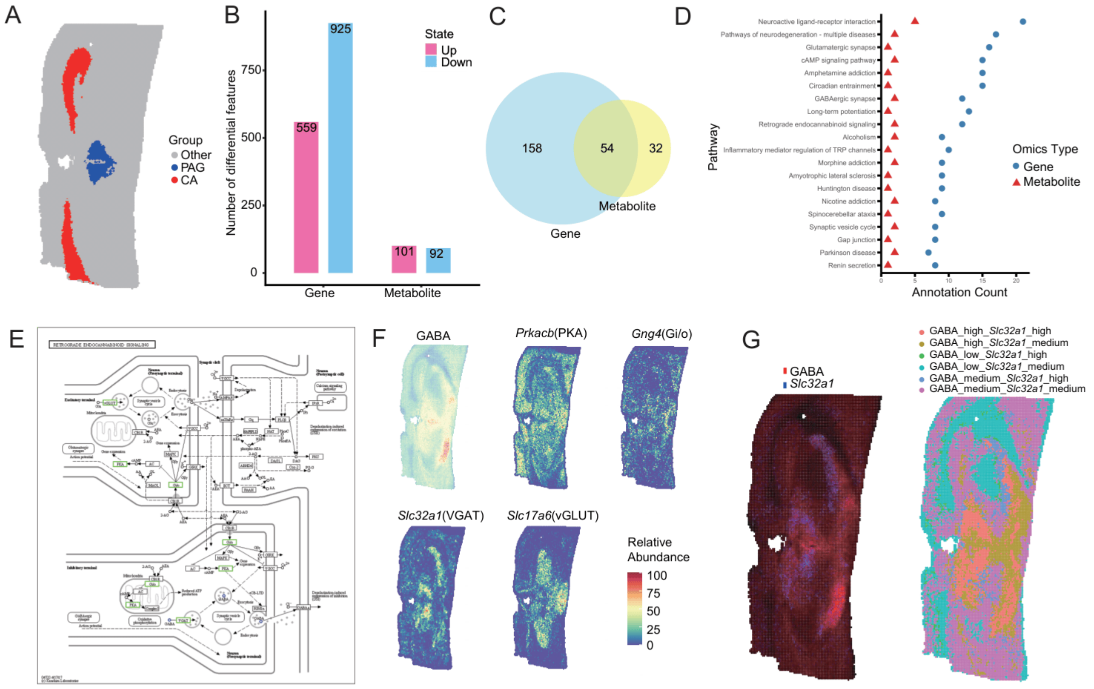

Figure 4 Interactive spatial selection unveils region-specific multi-omics functional pathways

A. Group comparison selection based on brain regions, with the experimental group as cornu ammonis (CA) and the control group as periaqueductal gray (PAG). **B.** Number of differentially expressed genes and mass features in the CA and PAG regions. **C.** Number of annotated pathways for differential genes and mass features in the CA and PAG regions. **D.** Pathway annotation for differential features in the CA and PAG regions. **E.** Pathway Retrograde endocannabinoid signaling annotated with mass features and genes in the PAG region. **F.** Abundance distribution maps of enriched genes and mass features in the PAG region. **G.** Spatial co-imaging (left) and co-expression (right) of *Slc32a1* and GABA.

merged figures with marks
Click here to access/download;Figure;alt-text-figure-accessibility-marked.docx

Figure accessibility and alt text

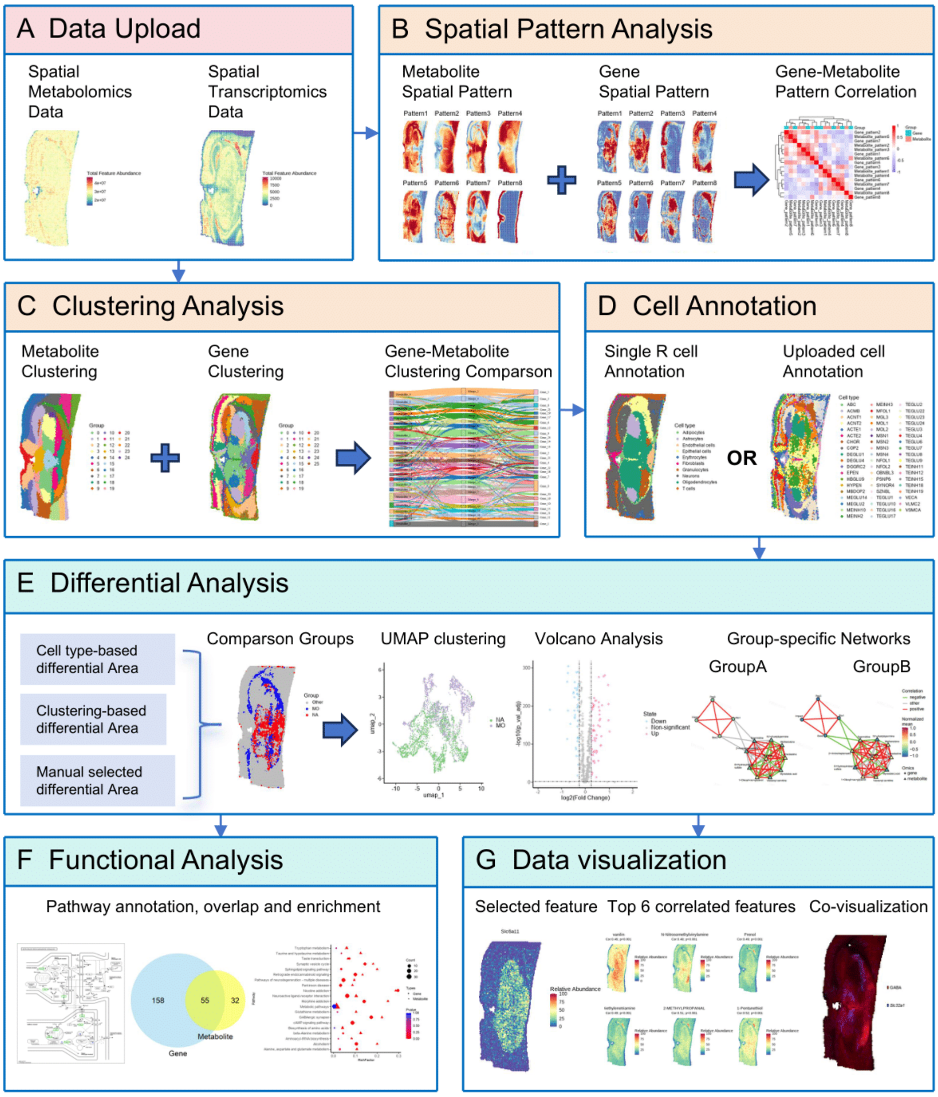
A Data Upload
Spatial Metabolomics Data
Spatial Transcriptomics Data
B Spatial Pattern Analysis
Metabolite Spatial Pattern
Gene Spatial Pattern
Gene-Metabolite Pattern Correlation
C Clustering Analysis
Metabolite Clustering
Gene Clustering
Gene-Metabolite Clustering Comparson
D Cell Annotation
Single R cell Annotation
OR
Uploaded cell Annotation
E Differential Analysis
Cell type-based differential Area
Clustering-based differential Area
Manual selected differential Area
Comparson Groups
UMAP clustering
Volcano Analysis
Group-specific Networks
GroupA
GroupB
F Functional Analysis
Pathway annotation, overlap and enrichment
158
55
32
Gene
Metabolite
G Data visualization
Selected feature
Top 6 correlated features
Co-visualization

2

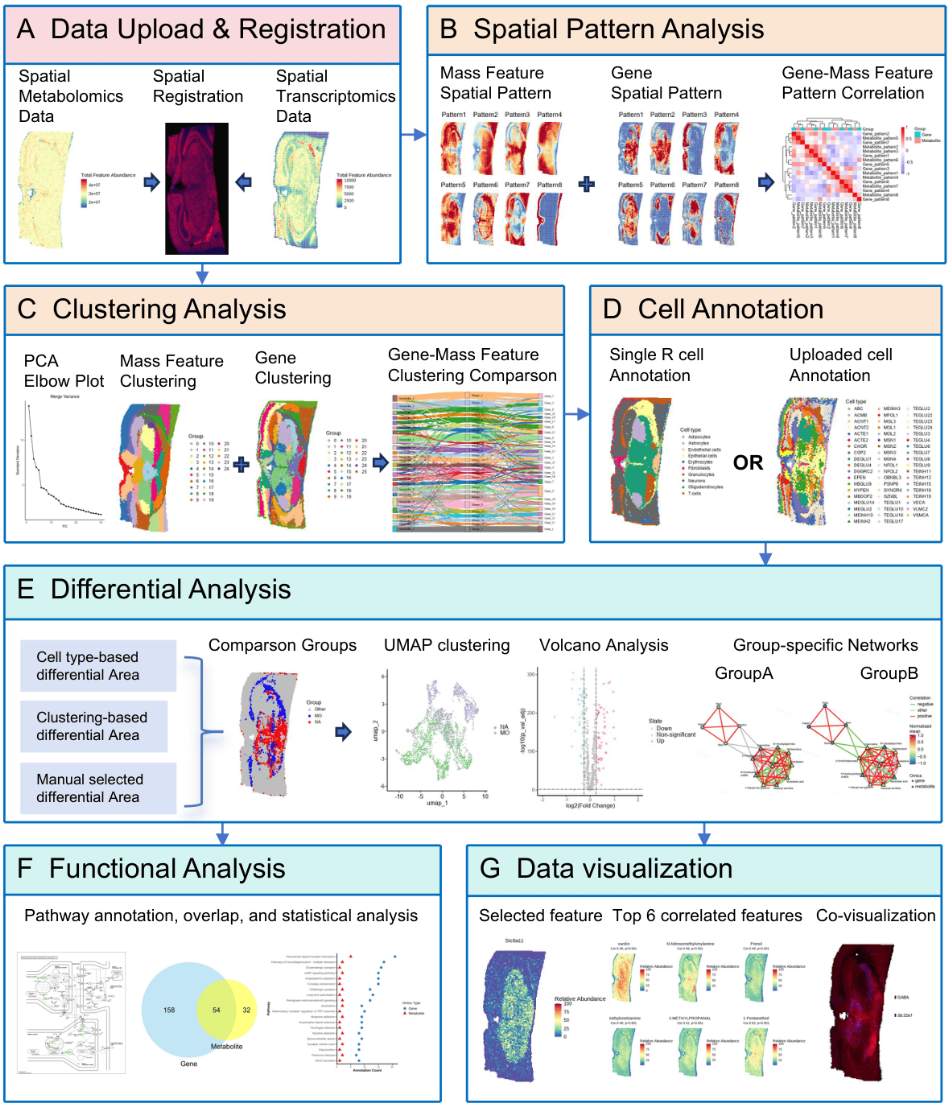

Figure 1 Overall architecture of SMIntegration

A. Data Upload and Registration. **B.** Spatial pattern analysis, identifying features with specific spatial patterns. **C.** Clustering analysis. **D.** Cell type annotation analysis. **E.** Differential analysis, allowing the selection of corresponding differential features through three different methods. **F.** Functional association analysis of differential features. **G.** Imaging visualization.

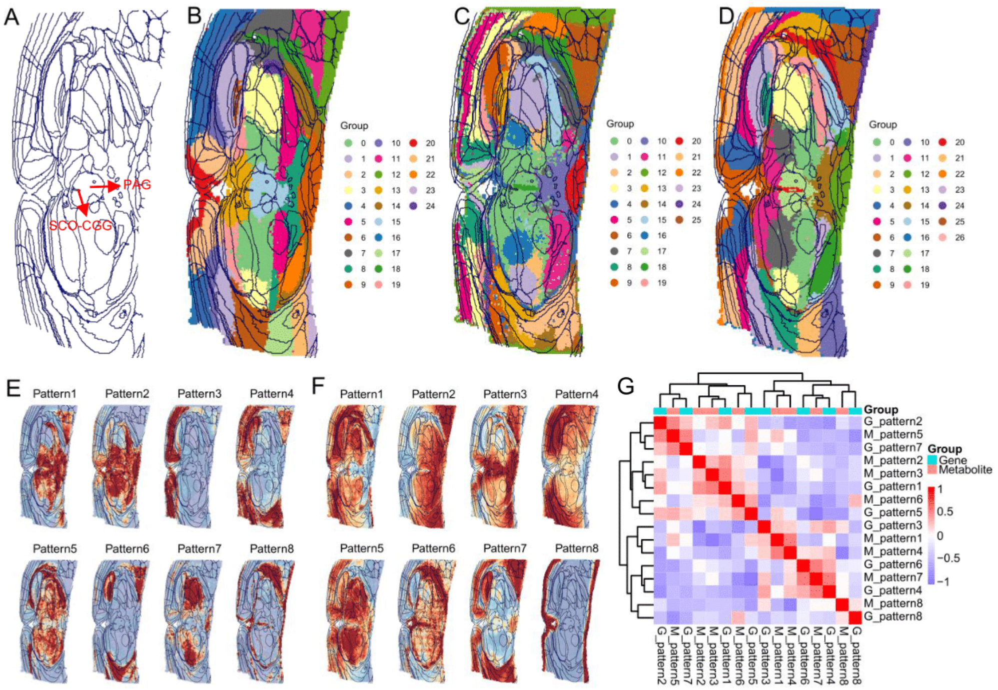

Figure 2 Integrated multi-omics clustering identifies conserved spatial domains and correlated molecular patterns

A. Schematic of mouse brain structure (atlas from ABA_Mouse_CCFv3_2017_25um.cutlas). **B.** Spatial metabolomics clustering. **C.** Spatial transcriptomics clustering. **D.** Integrated two-omics clustering map. **E.** Spatial pattern identification from spatial transcriptomics data. **F.** Spatial pattern identification from spatial metabolomics data. **G.** Spatial correlation between spatial metabolomics and spatial transcriptomics patterns. (The spatial pattern maps in B-F include brain region outlines for easier identification.)

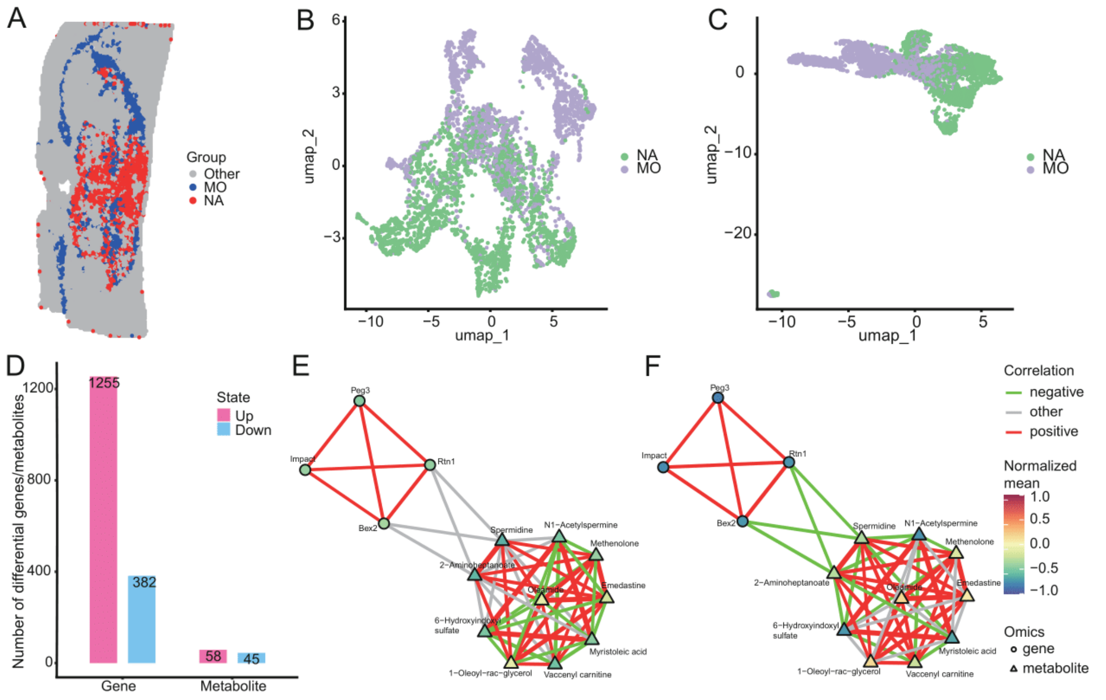

Figure 3 Cell type-based differential analysis reveals cell-enriched ~~metabolites~~mass features and genes.

A. Group comparison selection based on cell types, with the experimental group being non-telencephalon astrocytes (NA) and the control group being mature oligodendrocytes (MO). **B.** UMAP analysis of ~~metabolites~~mass features in the MO and NA regions. **C.** UMAP analysis of genes in the MO and NA regions. **D.** Number of differentially expressed genes and ~~metabolites~~mass features in MO and NA. **E.** Correlation network between differential ~~metabolites~~mass features and genes in the NA region. **F.** Correlation network between differential ~~metabolites~~mass features and genes in the MO region.

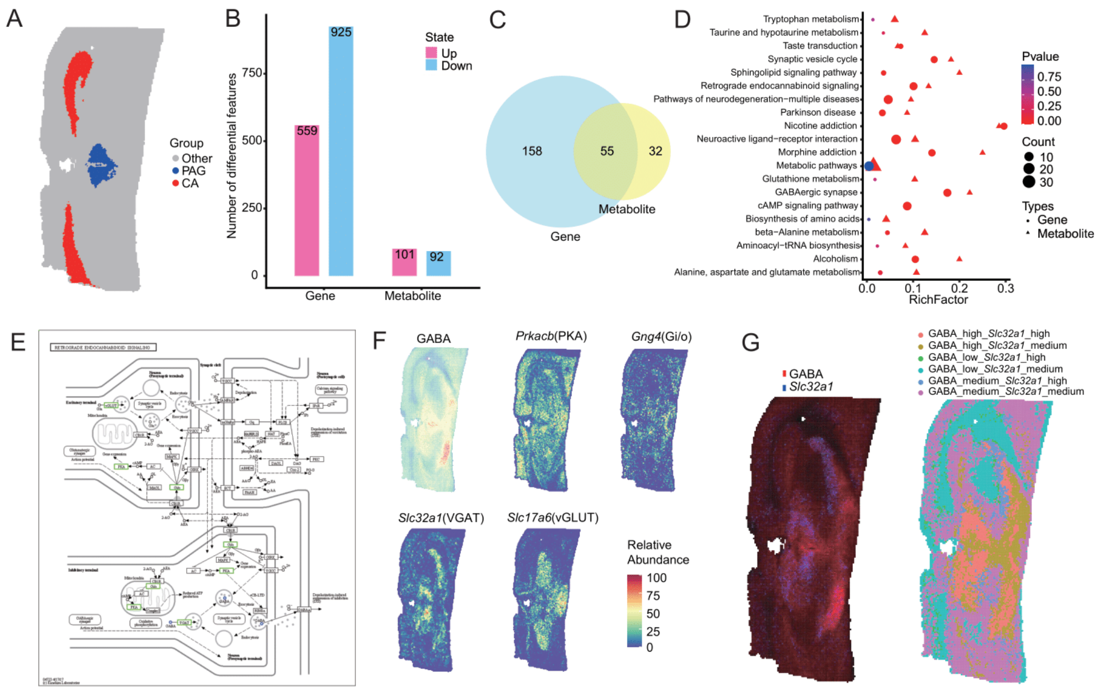

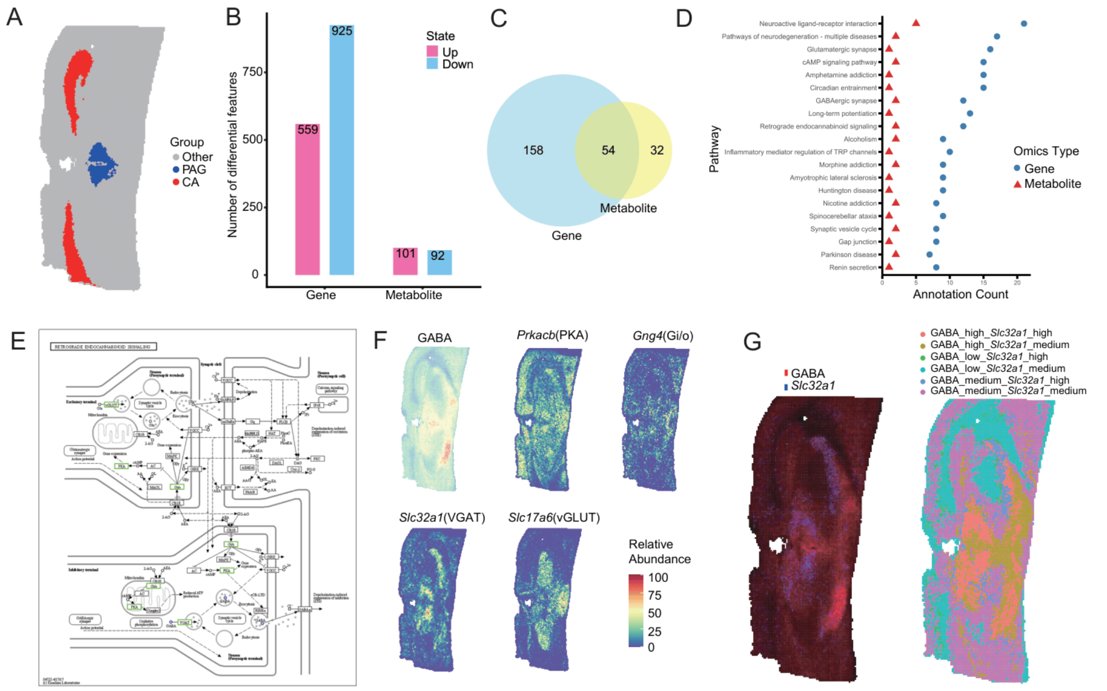

Figure 4 Interactive spatial selection unveils region-specific~~enriched~~ multi-omics functional pathways

A. Group comparison selection based on brain regions, with the experimental group as cornu ammonis (CA) and the control group as periaqueductal gray (PAG). **B.** Number of differentially expressed genes and ~~metabolites~~mass features in the CA and PAG regions. **C.** Number of annotated pathways for differential genes and ~~metabolites~~mass

features in the CA and PAG regions. **D.** Pathway annotation~~enrichment~~ for differential features in the CA and PAG regions. **E.** Pathway Retrograde endocannabinoid signaling annotated~~enriched~~ with ~~metabolites~~mass features and genes in the PAG region. **F.** Abundance distribution maps of enriched genes and ~~metabolites~~mass features in the PAG region. **G.** Spatial co-imaging (left) and co-expression (right) of *Slc32a1* and GABA.

Figure1

Click here to access/download;Figure;figure1.pdf

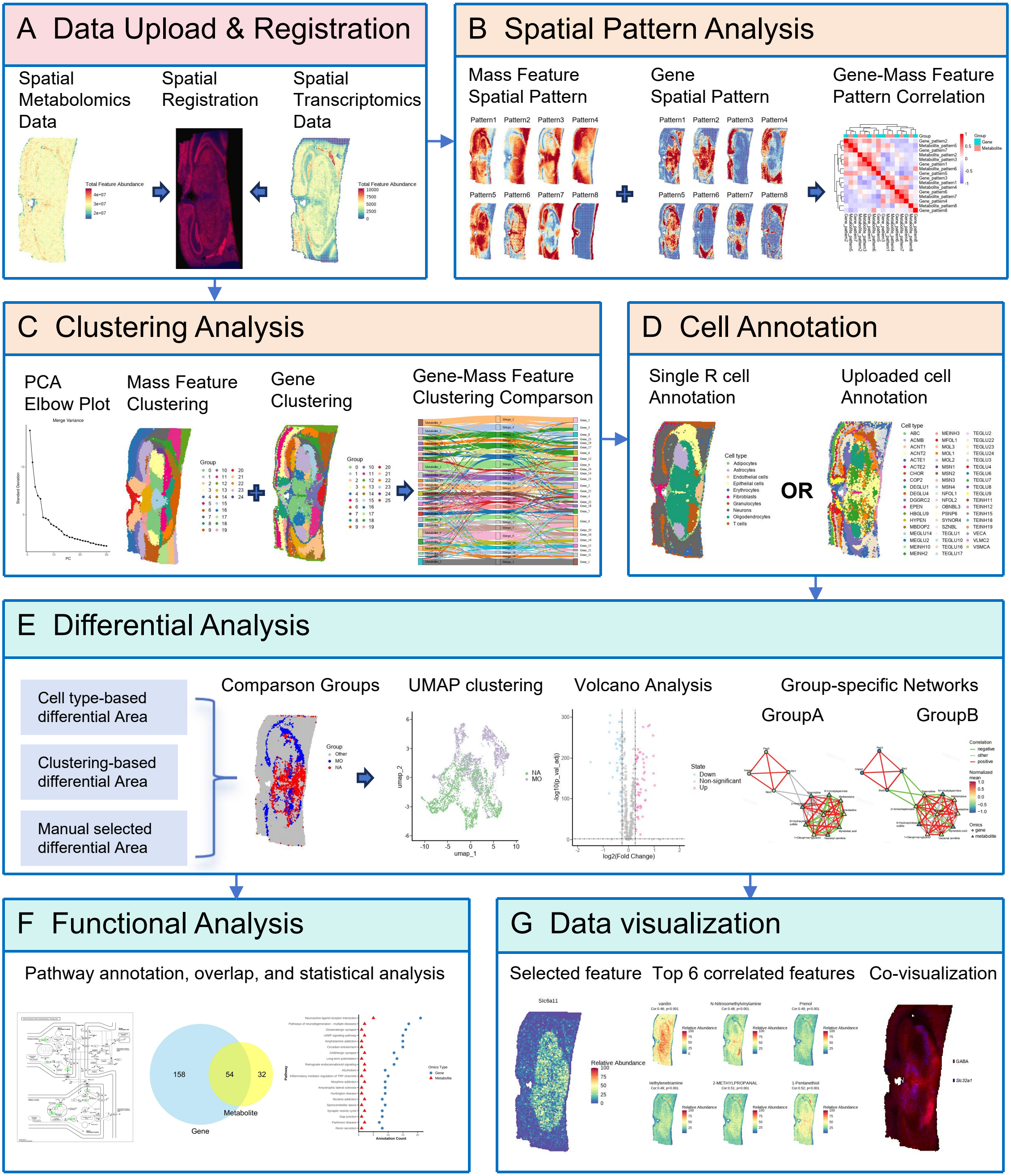

Figure4

Click here to access/download;Figure;figure4.pdf

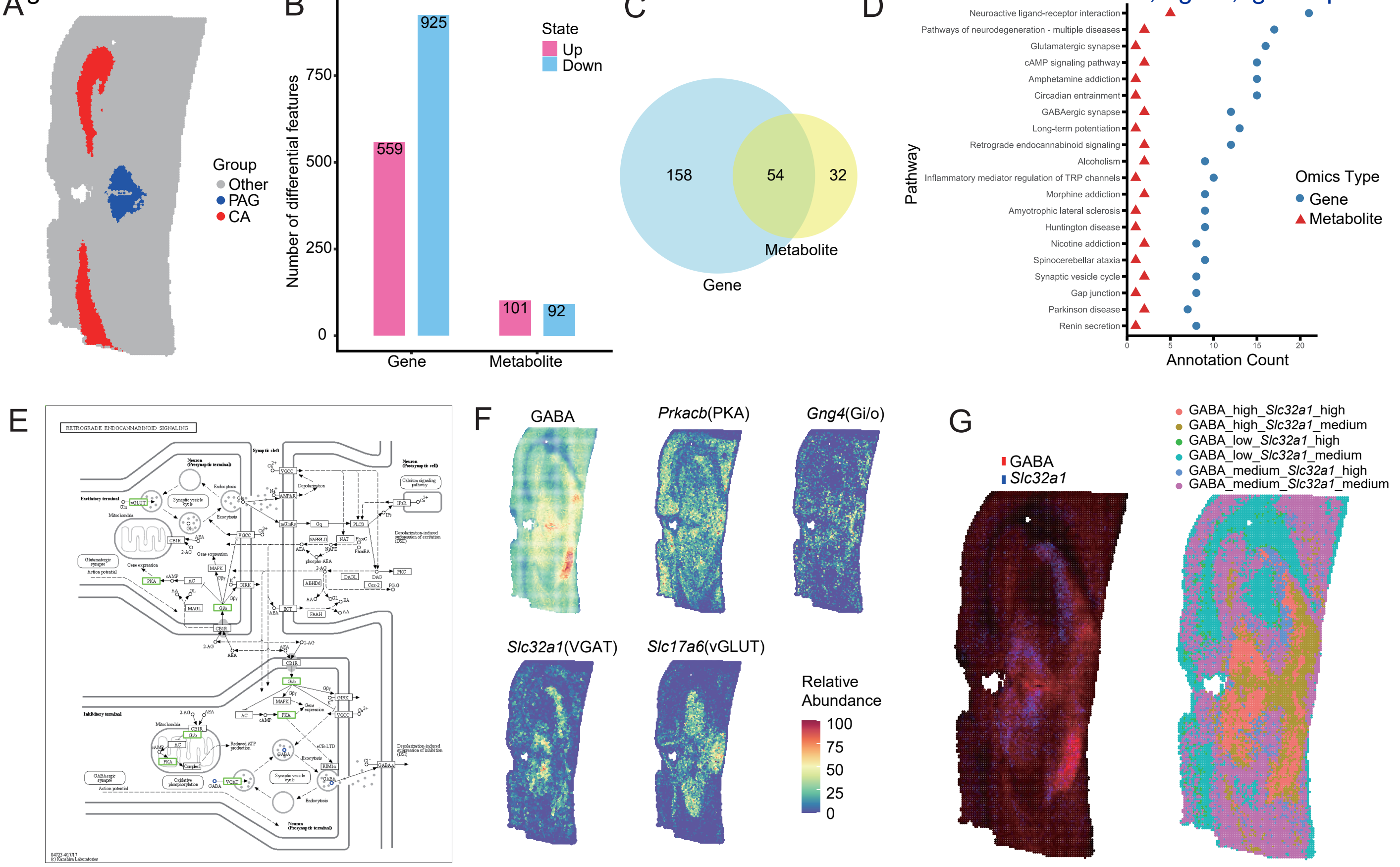

A video demonstrating differential analysis based on cell type

Click here to access/download
Supplementary Material
ROI.mp4

Supplementary Figure 1

Click here to access/download
Supplementary Material
Figure_S1.pdf

Supplementary Figure 10

Click here to access/download
Supplementary Material
Figure_S10.pdf

Supplementary Figure 14

Click here to access/download
Supplementary Material
Figure_S14.pdf

Supplementary Figure 3

Click here to access/download
Supplementary Material
Figure_S3.pdf

Supplementary Figure 5

Click here to access/download
Supplementary Material
Figure_S5.pdf

Supplementary Figure 6

Click here to access/download
Supplementary Material
Figure_S6.pdf

Supplementary File 1

Click here to access/download
Supplementary Material
Supplementary_File_1.pdf

Supplementary File 2

Click here to access/download
Supplementary Material
Supplementary_File_2.pdf

coverletter

Click here to access/download;Personal Cover;CoverLetter.docx

February 12, 2026

Submission Type: Technical Note

Dear Ms. Fan,

We are pleased to submit the revised version of our manuscript entitled "SMIntegration: A Web Tool for Comprehensive Spatial Metabolomics and Transcriptomics Integrated Analysis and Visualization" (Manuscript ID: GIGA-D-25-00440) for consideration in GigaScience.

We sincerely thank the editor and reviewers for their insightful comments and constructive suggestions, which have greatly helped us improve the scientific rigor, technical completeness, and accessibility of our manuscript. In this revised version, we have carefully addressed all the editorial and reviewer comments point-by-point.

Major revisions include:

1. Compliance with Editorial Policies:
    1) Software Registration: We have successfully registered the software in bio.tools (biotoolsID: smintegration) and SciCrunch (RRID: SCR_027925). The computational workflow has been archived in WorkflowHub (DOI: 10.48546/workflowhub.workflow.2074.6). These identifiers have been included in the "Data Availability Statement", and the workflow DOI has also been cited in the Methods section.
    2) URL Standardization: All web links and URLs have been removed from the main text and moved to the Reference list or Data Availability Statement as requested.
    3) Author Information: ORCID identifiers for all authors have been updated in the title page and submission system.
2. Functional Enhancements & Technical Upgrades:
    1) Implemented a dedicated Spatial Registration module within the GUI (using RNiftyReg) to address the coregistration barrier raised by Reviewer 1 and 2.
    2) Integrated PCA-based visualization for objective clustering parameter selection.
    3) Refined the preprocessing pipeline by explicitly decoupling normalization options (TIC and RMS) from data transformation, ensuring greater transparency and alignment with MSI standards (Reviewer 3).

3. Analytical Rigor & Transparency:
    1) Transitioned from "functional enrichment" to a more robust "Pathway Annotation" workflow to address the identification uncertainty inherent in spatial metabolomics (Reviewer 1 & 3).
    2) Clarified the algorithmic logic for cell-type specific association discovery (Reviewer 2).
    3) Implemented a dual-layer validation suite (modular unit tests and figure reproduction scripts) and added comprehensive code comments to ensure software reproducibility (Reviewer 1).

4. Refined Scientific Interpretation:
Systematically updated terminology (e.g., replacing "metabolites" with "mass features" where appropriate) and softened exploratory conclusions to avoid over-interpretation.
All changes have been clearly highlighted in the revised manuscript, and detailed point-by-point responses to the editor and each reviewer are included in the attached "Response to Reviewers" document.
We believe that the revised manuscript now presents SMIntegration as a robust, fully compliant, and user-friendly platform that significantly lowers the technical barrier for spatial multi-omics integration.

Thank you for considering our revised manuscript. We look forward to your decision. As the Chinese New Year begins, I would like to take this moment to wish you a joyful and prosperous Year of the Horse.

Sincerely,

Zhanlong Mei, Ph.D.
Corresponding Author
BGI Genomics, Shenzhen, China

Jin Zi, Ph.D.
Co-Corresponding Author
BGI Genomics, Shenzhen, China

respond to reviewer3 Click here to access/download;Personal Cover;reviewer3.docx

Response to Reviewer Comments

1. Summary

We would like to express our sincere gratitude to the reviewers for their insightful comments and constructive suggestions. Their feedback has been invaluable in enhancing the scientific rigor, technical completeness, and clarity of our manuscript. In response to the reviewers' concerns, we have performed a comprehensive revision of both the SMIntegration platform and the manuscript. The key highlights of our revisions are summarized below:

1) Significant Functional Enhancement: To address the critical need for multi-modal alignment, we have implemented a dedicated Spatial Registration module within the SMIntegration GUI. This module utilizes the RNiftyReg framework to support both linear and non-linear transformations, providing a seamless transition from raw data to integrated analysis.
2) Enhanced Analytical Rigor & Transparency: Following the reviewers' suggestions, we have refined our preprocessing pipeline by explicitly decoupling normalization options (TIC and RMS) from data transformation. This modular approach ensures greater transparency and strictly aligns with mass spectrometry imaging (MSI) reporting standards. Furthermore, we have updated our terminology throughout the manuscript—replacing "metabolites" with "mass features"—to accurately reflect the Level 3 (tentative) identification confidence associated with MS1-based annotation.
3) Clarified Platform Scope and Positioning: We have carefully revised the manuscript to define SMIntegration as a specialized downstream integration platform. We have clarified that primary raw data processing (e.g., peak detection) remains external to the platform to preserve methodological flexibility and ensure computational scalability for concurrent users.
4) Detailed Methodological Justification: We have expanded our discussion on the statistical rationale behind our default differential analysis thresholds, emphasizing their suitability for capturing subtle molecular gradients within high-density spatial microenvironments.

We believe that these revisions significantly strengthen the utility of SMIntegration as a robust, user-friendly tool for the spatial omics community. Detailed responses to each of the reviewer's comments are provided below.

2. Point-by-point response to Comments and Suggestions for Authors

Comment 1: " Data preprocessing for spatial metabolomics data was done using the Cardinal package, and for spatial transcriptomics data using Seurat in this manuscript.

This significantly reduces user-friendliness and requires substantial programming skills to use SMIntegration, making this tool difficult for many users. Please consider integrating the data preprocessing steps directly into SMIntegration."

Response: We sincerely appreciate the reviewer's suggestion to enhance the user-friendliness of SMIntegration. We understand the value of an "all-in-one" solution; however, after careful consideration, we have decided to maintain SMIntegration's focus as a specialized downstream integration platform rather than a primary raw data processor. Our reasoning is based on the following:

1) Methodological Flexibility and Scalability: Spatial metabolomics peak detection and spatial transcriptomics preprocessing are both computationally intensive and highly dependent on experimental design, instrumentation, and user-defined parameters. Integrating these steps directly into a shared cloud-based platform would substantially increase computational burden, affect scalability for concurrent users, and limit methodological flexibility. By allowing users to upload pre-processed features, we ensure they can utilize the most appropriate parameters specific to their raw data acquisition.
2) Platform Positioning: The core mission of SMIntegration is to bridge the gap between two omics modalities. Most researchers in this field already have established preferences and standardized pipelines for single-modality data quality control. SMIntegration acts as the next step in the workflow, focusing on cross-modal pattern recognition and co-localization.

We have revised the manuscript to better manage user expectations regarding the software's scope:

Abstract: Clarified that the platform provides a downstream integration workflow starting from the pre-processed spatial features: "Built with R/Shiny and deployed using Docker containerization, the platform provides a complete integration workflow, starting from pre-processed spatial features through to functional annotation. "(Page 3, Line 53).

Methods (Data Preparation and Spatial Registration): Added a statement explicitly defining the platform's scope and explaining the rationale for handling primary raw data processing externally to maintain methodological flexibility. Revised text: "SMIntegration is specifically designed as a downstream integration platform. To maintain methodological flexibility and accommodate diverse experimental designs, primary raw data processing—such as peak detection for mass spectrometry imaging or initial analysis for spatial transcriptomics—is handled by specialized external tools. These steps are computationally intensive and highly dependent on instrumentation and user-defined parameters; thus, performing them externally ensures scalability for concurrent users on our cloud-based platform and preserves methodological rigor. "(Page 5, Line 124)

We believe these clarifications accurately define the tool's utility while ensuring it remains a high-performance, flexible solution for the spatial omics community.

Comment 2: " Please consider adding a registration function in SMIntegration using the SpatialData package, as this is one of the most critical steps for integrated spatial metabolomics and transcriptomics data analysis."

Response: We sincerely thank the reviewer for this insightful and valuable suggestion. We fully agree that spatial registration is the cornerstone of multi-modal integration, and providing an internal solution within the GUI would significantly lower the barrier for users.

In response to your suggestion, we have implemented a dedicated Spatial Registration module directly within the SMIntegration platform. While we carefully evaluated the SpatialData package as suggested, we ultimately chose to implement this module using the RNiftyReg package [17]. This decision was based on several factors:

Compatibility: RNiftyReg offers seamless integration with our R/Shiny-based architecture, ensuring high stability and performance within the web environment.

Algorithm Robustness: It provides access to the powerful NiftyReg library, supporting both efficient linear (block-matching) and flexible non-linear (free-form deformation) registration, which are essential for aligning different tissue sections or modalities.

User Experience: The integrated module allows users to perform registration and subsequent analysis within a single, unified environment without switching to external Python-based pipelines.

Specific changes made in the manuscript:

Abstract: We have highlighted the addition of the integrated spatial registration module as a core feature of SMIntegration. Revised text: " Its core functions include: (1) automated and interactive spatial registration; (2) cross-modal spatial pattern recognition; (3) flexible differential analysis of genes and mass features based on clustering results, user-defined regions, or cell type annotations; and (4) group-specific gene-metabolite network construction and interactive visualization. " (Page 3, Line 55).

Methods Section: The "Data Preparation and Spatial Registration" section has been substantially revised to describe the new module. Revised text: "SMIntegration input requires processed spatial metabolomics and transcriptomics data matrix. Since resolutions differ, higher-resolution data should be aggregated to match the lower (e.g., binning 500 nm transcriptomics by 100 to 50 μm metabolomics) [16]. To facilitate seamless integration, SMIntegration features a dedicated spatial registration module

implemented using the RNiftyReg package [17], which provides an R interface to the NiftyReg library [18,19] and is part of the TractoR framework [20]. This module supports both linear (block-matching) and non-linear (free-form deformation) transformations to align the two modalities. Detailed tutorials for this registration module are provided in Supplementary File 1. Users also have the option to perform coregistration externally using the Python-based SpatialData ecosystem [21]; the aligned data can then be imported into SMIntegration in a compatible format for subsequent integrated analysis. "(Page 6, Line 131).

References: We have added five new references [16-20] to acknowledge the developers of the registration library and the TractoR framework.

Figure 1: We have updated the architecture diagram in Figure 1 to include the registration module as a key component of the data preprocessing workflow.

We believe this addition greatly enhances the utility and completeness of SMIntegration. Thank you again for your constructive guidance.

Comment 3: " Line 143: LogNormalization does not work well for spatial metabolomics data due to its highly sparse nature. More common methods are TIC normalization and root mean squared normalization."

Response: We sincerely thank the reviewer for this insightful comment regarding normalization standards in mass spectrometry imaging (MSI). We agree that pixel-wise normalization methods like TIC and RMS are the community standard for mitigating technical artifacts in MSI data.

We would like to clarify that Seurat's NormalizeData function, when the 'LogNormalize' method is selected, inherently performs Total Ion Current (TIC) normalization. This method works by first dividing the intensity of each feature by the total sum of intensities in that pixel (which is the definition of TIC normalization) before applying the logarithmic transformation. Therefore, the original pipeline effectively included the TIC step.

However, we fully accept the reviewer's point that using "LogNormalize" as a blanket term obscures this critical step and that RMS normalization is an equally important alternative not covered by the default Seurat function.

To address this and improve methodological transparency:

1. Exposed Normalization Options: We have decoupled the normalization step from the transformation step in our GUI. Users can now explicitly select "TIC" (Total Ion

Current), "RMS" (Root Mean Square), or "None" as the primary normalization method.

2. Modular Pipeline: We have refined the preprocessing workflow into three distinct stages: (1) Normalization (TIC/RMS), (2) Transformation (Log1p), and (3) Scaling (Z-score). This allows users to customize the normalization and transformation strategies to adhere to their specific MSI data processing standards.

Changes in the manuscript: "The preprocessing pipeline follows a standardized workflow to handle the sparsity and technical variance of spatial data: (1) Normalization where users can choose Total Ion Current (TIC) or Root Mean Squared (RMS) normalization to account for pixel-wise technical variation; (2) Transformation, such as LogNormalize to stabilize variance; and (3) Scaling and variable feature selection (top 2000 genes/mass features). This modular design allows users to customize each step or skip them if the input data has been pre-processed." (Page 7, Line 172).

We believe this change significantly enhances the clarity and flexibility of the platform for metabolomics researchers.

Comment 4: " Line 90: Add the word "spatial" (e.g., "spatial metabolomics")."

Response: Thank you for your careful reading and valuable suggestion. The comment to emphasize the spatial dimension is well-noted and important for accurately defining the scope of our study within spatial multi-omics integration.

To further enhance precision, we have specified both omics layers, now stating: "No standardized pipeline exists for spatial transcriptomics and spatial metabolomics integration." (Page 4, Line 90)

This modification more clearly frames the methodological gap our work addresses. We appreciate your insightful feedback, which has helped improve the clarity of our manuscript.

Comment 5: " Line 100: Your workflow does not include the complete processing steps. Please consider removing the word "preprocessing.""

Response: Thank you for this constructive comment. You are correct that the term "preprocessing" could be interpreted as encompassing raw data processing steps which may not be fully covered by our integration platform. We agree that removing this specific term enhances the accuracy of our description.

Accordingly, we have revised the sentence at Line 100 to state: "It provides a streamlined downstram integration pipeline lowering the technical barriers." (Page 4, Line 99)

This revision more precisely describes our platform's scope in facilitating the integrative analysis of preprocessed data. We appreciate your suggestion for improving the clarity of our manuscript.

Comment 6: " Line 159: Why is the default threshold for the fold-change value so small?"

Response: We thank the reviewer for this constructive question. We agree that the threshold of |log2FC| > 0.26 appears smaller than the values typically used in traditional bulk omics. However, we chose this default value based on the specific characteristics of spatial/single-cell data and established community standards:

1) Biological Sensitivity in Microenvironments: A |log2FC| of 0.26 translates to a 1.2-fold change. In high-resolution spatial analysis, a 20% difference in protein or metabolite abundance can represent critical biological transition zones or boundary effects that would be obscured by a more stringent 2-fold threshold.
2) Statistical Power of Pixel-wise Analysis: Our workflow treats each pixel as an individual observation. This high-density sampling provides the statistical power to detect smaller effect sizes with high confidence, ensuring that the identified markers are statistically robust despite a lower FC.
3) Alignment with Community Standards: The threshold of 0.26 closely approximates the default setting for the FindMarkers function in Seurat, the most widely used framework for single-cell and spatial analysis. Adopting this value ensures our software remains consistent with the analytical habits of the broader research community.
4) Parameter Flexibility: We have clarified in the revised manuscript that this threshold is a customizable parameter. While 0.26 is the default for high-sensitivity discovery, users can easily adjust this value within the software interface to meet their specific stringency requirements.

We have updated the manuscript to include this justification and to highlight the adjustability of the parameter. Revised text: " Results are Bonferroni-corrected [28], with default thresholds of |log2FC| > 0.26 and adjusted p < 0.05. The threshold of 0.26 corresponds to a ~1.2-fold change, which is comparable to standard defaults in pipelines like Seurat. This allows for the detection of subtle molecular gradients and fine-scale heterogeneity in the tissue. Notably, this parameter is fully customizable, allowing users to apply more stringent filters depending on their specific biological questions and sample heterogeneity. "(Page 8, Line 201)

Comment 7: " Line 189: The authors appear to confuse "mass features/peaks" with "metabolites." Please replace "metabolites" with "mass peaks" or "mass features" throughout the manuscript and in Figure 1."

Response: We appreciate the reviewer's guidance on the precise use of metabolomics terminology. We acknowledge that the term "metabolic features" or "annotated features" is more scientifically accurate than "metabolites" when identifications are tentative (Level 3) and when multiple peaks may derive from a single molecular entity (e.g., adducts and isotopes).

In accordance with your suggestion, we have replaced the term "metabolites" with "mass features" throughout the entire manuscript, including the Abstract, Results, and Discussion sections. We have also updated Figure 1 and its legend to reflect this change.

Comment 8: " Line 188: SManalyst identifies metabolites only through monoisotopic ion matching, which is considered Level 3 metabolite identification. The authors should state this clearly in the manuscript and exercise caution when interpreting the data throughout the manuscript."

Response: We sincerely thank the reviewer for this insightful and valuable comment. We fully agree that metabolite identification based solely on monoisotopic mass matching (MS1), as performed by the SManalyst platform, corresponds to Level 3 (tentative candidates).

As suggested, we have explicitly stated this identification level in the revised manuscript and added a statement to emphasize that the interpretation of these data was conducted with appropriate caution. We have added a new reference (Schymanski et al., 2014) to clarify the confidence levels in metabolite identification.

Changes in the manuscript: " Metabolite identification was performed using the SManalyst platform [30] based on monoisotopic mass matching yielding 13,707 pixels and 560 annotated metabolic features. It is important to note that this approach constitutes Level 3 annotation according to current metabolomics reporting standards [31]. Therefore, all subsequent references to metabolites in the context of these data should be interpreted with caution, as they represent putatively annotated features rather than definitively identified compounds." (Page 9, Line 237)

We believe these revisions improve the transparency and accuracy of our reporting. Thank you again for your constructive guidance.

References

[16] Chen A, Liao S, Cheng M, Ma K, Wu L, Lai Y, et al. Spatiotemporal transcriptomic atlas of mouse organogenesis using DNA nanoball-patterned arrays. Cell 2022; 185(10): 1777–1792.e21.

[17] Clayden J, Modat M, Presles B, Anthopoulos T, Daga P. RNiftyReg: Image registration using the “NiftyReg” library. R package version 2.8.4, 2024.

[18] Modat M, Cash DM, Daga P, Winston GP, Duncan JS, Ourselin S. Global image registration using a symmetric block-matching approach. J Med Imaging (Bellingham) 2014; 1: 024003.

[19] Modat M, Ridgway GR, Taylor ZA, Lehmann M, Barnes J, Hawkes DJ et al. Fast free-form deformation using graphics processing units. Comput Methods Programs Biomed 2010; 98: 278–284.

[20] Clayden JD, Maniega SM, Storkey AJ, King MD, Bastin ME, Clark CA. TractoR: Magnetic Resonance Imaging and Tractography with R. J. Stat. Soft. 2011; 44: 1–18.

[31] Schymanski EL, Jeon J, Gulde R, Fenner K, Ruff M, Singer HP, et al. Identifying Small Molecules via High Resolution Mass Spectrometry: Communicating Confidence. Environ Sci Technol 2014; 48:2097–8.

respond to reviewer1 Click here to access/download;Personal Cover;reviewer1.docx

Response to Reviewer Comments

1. Summary

We would like to express our sincere gratitude to the reviewer for their critical and constructive feedback. These comments have been instrumental in identifying key barriers to usability and technical reproducibility in our initial submission. In response, we have performed a major overhaul of the SMIntegration platform, codebase, and manuscript. The key highlights of our revisions are summarized below:

1) Integrated Spatial Registration: Addressing the critical concern regarding the difficulty of data coregistration, we have implemented a dedicated Spatial Registration module within the SMIntegration GUI. Utilizing the RNiftyReg framework, this module allows users to perform both linear and non-linear transformations directly within the platform, eliminating the need for undocumented external scripts.

2) Enhanced Analytical Rigor and Transparency: We have refined our analytical strategies to ensure robustness and clarity. This includes: (1) integrating PCA-based visualization to objectively guide clustering parameter selection; (2) justifying our differential analysis thresholds based on spatial biological sensitivity; and (3) transitioning from "functional enrichment" to a more transparent "Pathway Annotation" workflow to address the specific challenges of spatial metabolomics data interpretation.

3) Comprehensive Documentation and Validation: We have completely restructured our GitHub repository to meet high software standards. This includes systematic code commenting for all scripts and the implementation of a dual-layer validation suite (modular unit tests and figure reproduction scripts) to ensure computational reliability. Additionally, we have expanded user resources with step-by-step tutorials and integrated help manuals.

4) Refined Scientific Interpretation: We have carefully revised the manuscript's language to better reflect the exploratory nature of our findings, replacing over-interpretative terms with more precise descriptions of observed spatial patterns and associations.

We believe these substantial improvements effectively lower the technical barriers for users and establish SMIntegration as a rigorous, user-friendly tool for the spatial omics community. Detailed responses to each of the reviewer's comments are provided below.

2. Point-by-point response to Comments and Suggestions for Authors

Comment 1: "First, the software requires metabolomics and transcriptomics data which has already been coregistered and transformed to the same spatial resolution. These two tasks - especially coregistration - are challenging and a barrier to

researchers intending to use the presented package. The authors point to a script on their github to coregister data, but this has zero documentation so is not a viable option. Please give better instructions and point to other options for these tasks."

Response: We sincerely thank the reviewer for this critical feedback. We agree that data coregistration and resolution alignment are significant steps in multi-omics workflows. We have taken the following actions to address these concerns:

1. Lowering the Barrier via Integrated Registration To eliminate the need for external, undocumented scripts, we have supplemented the Overall Distribution Analysis module with a robust registration function. When users select the 'Perform Registration' option, a comprehensive registration interface is presented directly within the GUI. This function utilizes the RNiftyReg package, providing an intuitive interface for both linear and non-linear (free-form deformation) transformations. This allows users to align different tissue sections or modalities within a single, unified web environment.

2. Standardized Resolution Alignment (Binning) Workflow: We would like to clarify that transforming different modalities to a common spatial resolution is now a standard and well-established operation in the spatial omics field. High-resolution spatial transcriptomics data (e.g., Stereo-seq at 500 nm resolution) is routinely aggregated or "binned" to reduce technical noise and match the resolution of other modalities [16].

SMIntegration follows this industry-standard practice by providing clear guidelines and internal logic for aggregating higher-resolution data to match the lower-resolution grid of spatial metabolomics (e.g., 50 µm).

3. Comprehensive Documentation and Tutorials: We have significantly improved our external resources and internal "Help" interface:

4.We have replaced the previous scripts with a comprehensive step-by-step tutorial (now included as Supplementary File 1). A new screenshot-based manual is available within the platform to guide users through the integrated registration process.

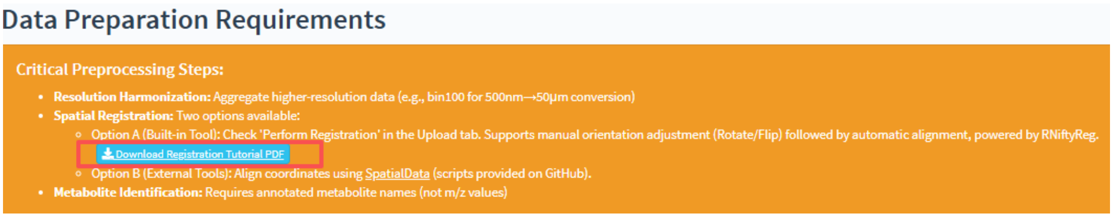

Specific changes made in the manuscript:

Abstract: Highlighted the addition of the "automated and interactive spatial registration" module. Revised text: "Its core functions include: (1) automated and interactive spatial registration; (2) cross-modal spatial pattern recognition; (3) flexible

differential analysis of genes and mass features based on clustering results, user-defined regions, or cell type annotations; and (4) group-specific gene-metabolite network construction and interactive visualization."(Page 3, Line 55)

Methods: Revised the "Data Preparation and Spatial Registration" section to describe the RNiftyReg-based module and the standard binning procedures. Revised text: " SMIntegration input requires processed spatial metabolomics and transcriptomics data matrix. Since resolutions differ, higher-resolution data should be aggregated to match the lower (e.g., binning 500 nm transcriptomics by 100 to 50 μm metabolomics) [16]. To facilitate seamless integration, SMIntegration features a dedicated spatial registration module implemented using the RNiftyReg package [17], which provides an R interface to the NiftyReg library [18,19] and is part of the TractoR framework [20]. This module supports both linear (block-matching) and non-linear (free-form deformation) transformations to align the two modalities. Detailed tutorials for this registration module are provided in Supplementary File 1. Users also have the option to perform coregistration externally using the Python-based SpatialData ecosystem [21]; the aligned data can then be imported into SMIntegration in a compatible format for subsequent integrated analysis. Two input formats are supported: (1) text matrices containing feature name, spatial x/y coordinates, and values (Figure S2A); (2) Seurat objects with coordinates and abundance in designated slots (Figure S2B). Upload requirements are detailed on the help page."(Page 6, Line 131)

Figures & Supplementary Material: Updated Figure 1 and added Supplementary File 1 to serve as a complete user manual, and updated Figure S1 and Figure S3 to showcase the enhanced platform interface.

References: We have added five new references [16-20] to acknowledge the developers of the registration library and the TractoR framework.

We believe these updates effectively address the technical barriers mentioned and ensure that SMIntegration provides a complete, user-friendly pipeline from raw data to biological insight.

Comment 2: " The methods employed require various parameters to be set. For example, the number of clusters or clustering resolution. How should users set these? It would be nice to provide some (semi)automatic methods for picking the best values (or at least objective assessment of the results, allowing different values to be compared). Defaults should be justified / explained. E.g. why is the default log2FC threshold 2.6? "

Response: We sincerely thank the reviewer for these insightful comments regarding parameter transparency and objective guidance. We agree that providing a basis for parameter selection is crucial for reproducible and rigorous spatial omics analysis. We have addressed these concerns from the following aspects:

1. Objective Guidance for Clustering Parameters via PCA. In response to the suggestion for more objective methods (and as detailed in our response to Comment 5), we have integrated Principal Component Analysis (PCA) into the clustering workflow. Users can now utilize PCA variance plots to evaluate the complexity of their datasets. By observing the "elbow" of the variance curve, users can objectively determine the number of dimensions or clusters that best represent the spatial variation in their data, rather than relying on arbitrary defaults.

2. Justification of the |log_2FC| > 0.26 Default Threshold. We would like to clarify that the default threshold is 0.26 (approximately a 1.2-fold change), not 2.6. We chose this value for several reasons: 1) Biological Sensitivity: In spatial microenvironments, molecular changes often occur as subtle gradients rather than binary switches. A threshold of 0.26 allows the platform to capture these biologically significant transition zones. 2) Statistical Power: Our pixel-wise analysis treats each registered pixel as an individual observation. This high-density sampling provides substantial statistical power, ensuring that even smaller fold changes remain statistically robust (adjusted $p < 0.05$). 3) Alignment with Standards: This threshold closely mirrors the default settings of the widely used Seurat framework, ensuring that SMIntegration aligns with established community standards.

Specific changes made in the manuscript:

1. Added a new paragraph justifying the default thresholds and describing the role of PCA in guiding parameter selection. Revised text: "Five clustering methods are available (Figure 1C): Louvain (LV), LM, SLM [24], K-means after Principal Component Analysis (PCA), and K-means after Uniform Manifold Approximation and Projection（UMAP) [25,26]. To assist users in objective parameter selection, PCA is implemented as an initial visualization step to capture major spatial variation across many features. Users can leverage PCA plots to estimate the underlying complexity of the spatial domains and thus justify the selection of the number of clusters for subsequent analysis (Supplementary File 2). " (Page 7, Line 166).

2. Added a specific justification for the default log2FC threshold and highlighted its customizability. Revised text:" The threshold of 0.26 corresponds to a ~1.2-fold change, which is comparable to standard defaults in pipelines like Seurat. This allows for the detection of subtle molecular gradients and fine-scale heterogeneity in the tissue. Notably, this parameter is fully customizable, allowing users to apply more stringent filters depending on their specific biological questions and sample heterogeneity." (Page 8, Line 202).

Figure 1: We have updated the architecture diagram in Figure 1C to include PCA as part of the dimensionality reduction and clustering workflow.

Supplementary Material: We have added a new file (Supplementary File 2) demonstrating how PCA results can be used to inform the selection of cluster numbers

for the mouse brain dataset, and updated Figure S5 to showcase the refined clustering interface which now integrates these assessment tools.

Software Interface: Updated the interface of the "Clustering Analysis and Cell Annotation" module to provide a step-by-step guide on how to interpret PCA and clustering results for parameter optimization.

Comment 3: " Some of the language, to my mind, over interprets the data. For example l169 "understanding of group specific regulatory mechanisms" I don't think you can infer mechanism directly from this type of exploratory analysis. Line 234 "discovery provides a novel molecular framework" - this is a potential hypothesis that can be tested, I don't think you can say it is a 'discovery'. Line 270 "strongly suggests the presence of a finely tuned inhibitory regulation" - this is just a correlation, and not 'strong' evidence in my opinion."

Response: We fully agree with the reviewer’s perspective. As an exploratory analysis tool, the primary goal of SMIntegration is to help researchers generate biologically plausible hypotheses and identify potential associations rather than providing direct proof of underlying biological mechanisms. We have carefully reviewed the manuscript and revised the language to better reflect the exploratory nature of the findings, softening our tone where necessary.

Specific changes made in the manuscript:

Regarding the description of regulatory mechanisms: We have replaced "mechanisms" with "patterns" to avoid over-interpretation. Revised text: "By enforcing the same node layout across groups (Figure S9), users can intuitively observe changes in gene-metabolite correlation patterns, facilitating identification of candidate group-specific gene–metabolite association patterns." (Page 8, Line 215).

Regarding the "molecular framework": We have changed "discovery" to "finding" and used more cautious language to describe the suggested framework. Revised text: "This finding offers a potential molecular basis for investigating region-specific mechanisms of motor control and reward, highlighting the unique value of integrated spatial multi-omics analysis." (Page 11, Line 287).

Regarding the relationship between Bex2 and spermidine: We have removed the word "strongly" and rephrased the sentence to emphasize that the observation is a potential relationship based on correlation. Revised text: "This phenomenon reveals that the correlation between the molecules Bex2 and spermidine is highly specific to cellular function: the significant negative correlation observed in the MO region, where decreased Bex2 expression coincides with increased spermidine levels, points to the potential presence of a finely tuned inhibitory relationship—potentially mediated by spermidine accumulation—targeting cell proliferation and apoptosis." (Page 12, Line 325).

In addition to these specific points, we have conducted a thorough review of the entire manuscript to ensure that our conclusions are appropriately grounded in the data as exploratory findings. We appreciate the reviewer for helping us improve the scientific rigor of our presentation.

Comment 4: " Functional enrichment. For metabolomics this is not straightforward. Especially there will be uncertainty in the metabolite identities, as well as sparse mapping of metabolites to pathways. How are these aspects taken into account? At least the metabolite identification uncertainty should be acknowledge with a warning message. Is there a minimum number of metabolites/transcripts required to include a pathway? Mapping only a single metabolite to a pathway invalidates pathway testing. For enrichment (Fisher test), what background set is used? Use of the full set of e.g. KEGG compounds will greatly inflate p-values. What version of KEGG is used? What type of multiple testing is applied on for the pathway tests? Finally in the pathway enrichment plots, p-values are represented on a color scale 0-1 so it is hard to see any significance threshold. E.g. in figure 4D are any pathways significant? It would be clearer to visualise the log10 p-value here, with a specific mark for the cut-off. It also looks like the metabolites show higher rich factors than the transcripts. Rich factor is not defined, but if it relates to coverage of the pathway then this doesn't make sense - one would expect transcripts to have a higher coverage than metabolites."

Response: We sincerely thank the reviewer for this critical and insightful feedback regarding the challenges of functional enrichment in spatial metabolomics. We fully agree that the uncertainty in metabolite identification and the sparsity of pathway mapping present significant hurdles, and we have added a statement to emphasize that the interpretation of these data was conducted with appropriate caution.

Furthermore, we acknowledge the reviewer's concern that defining an appropriate background set for statistical testing (e.g., Fisher's exact test) is inherently difficult in MSI studies, where the detected metabolome represents only a fraction of the theoretical KEGG compound space (version 106.0). Using an overly broad background can indeed lead to inflated p-values and potentially misleading conclusions. Critically, we also recognize that the background sets for metabolites and transcripts are fundamentally different in size and composition. This inherent disparity makes the resulting p-values from separate enrichment analyses non-comparable, and their side-by-side visual presentation could be misleading.

Considering these valid concerns, we have refined our analytical strategy to prioritize biological robustness over statistical hypothesis testing based on uncertain assumptions. We have transitioned the module from a traditional "Enrichment Analysis" to a more transparent "Pathway Annotation Analysis" workflow. This approach focuses on reporting the count of annotated features (e.g., number of detected metabolites or transcripts mapped to a pathway) rather than comparing statistically derived p-values.

We believe this provides a more intuitive and comparable measure of data coverage across different omics layers.

Specific revisions include:

1. Methodological Shift: We removed the Fisher's exact test and P-value calculations. The analysis now ranks pathways based on the number of annotated differential features.

2.Quality Control: We automatically exclude overly broad pathways (e.g., “Metabolic pathways”).

3.Visualization: We replaced the p-value bubble plot with a Ranked Annotation Dot Plot (Figure 4D), where the x-axis represents the annotation count, and shapes distinguish between metabolomic and transcriptomic contributions.

Changes in Manuscript:

1. Clarified the metabolite identification level and added a cautionary statement regarding data interpretation. Revised text: " It is important to note that this approach constitutes Level 3 annotation according to current metabolomics reporting standards. Therefore, all subsequent references to metabolites in the context of these data should be interpreted with caution, as they represent putatively annotated features rather than definitively identified compounds." (Page 9, Line 239)

2. Systematically updated key terminology throughout the manuscript to align with the revised analytical logic. We have implemented a systematic terminology update throughout the entire manuscript (including the Introduction, Results, Discussion, and Figure Legends) to align with the revised analytical approach. Specifically, we have replaced the following key terms:

(1) “functional enrichment” has been replaced with “functional annotation”.

(2) In the context of pathway analysis, terms such as “enriched” and “significantly enriched” have been replaced with “annotated”.

(3) “significant co-enrichment” and “co-enrichment” has been replaced with “co-annotation”.

3. Updated the method description for the revised functional analysis module. Revised text: "*Functional Association and Annotation*. This module integrates and interprets biological functions of DEGs and DAMs (Figure 1F). It performs pathway mapping (e.g., to KEGG pathways, version 106.0) and quantifies the number of DEGs and DAMs co-annotated to each pathway. Overly broad global pathways (e.g., "Metabolic pathways") are excluded. Pathway nodes are color-coded to indicate their up- or down-

regulation, allowing users to visualize the spatial distribution of all annotated DEGs and DAMs for any given pathway (Figure S10)." (Page 9, Line 219)

4. Revised results descriptions to consistently use the updated annotation-based terminology. Revised text:

"Differential analysis identified 1484 differential genes (Figure 4B), corresponding to 212 pathways, and 193 differential mass features, corresponding to 86 pathways. There were 54 pathways shared by both differential genes and mass features (Figure 4C). " (Page 13, Line 344)

"Figure 4D shows the pathways containing these differential features. Genes and mass features highly expressed in the CA region were annotated to pathways such as Glutamatergic synaps..." (Page 13, Line 348)

"Pathways containing DEGs and DAMs highly expressed in the PAG region point to its core functions" (Page 13, Line 357)

5. Updated relevant figures and their legends to reflect the terminology and methodological changes: We have also updated Figure 4 and Supplementary Figures ((S10, S14) and its legend to reflect this change.

Comment 5: " Spatial pattern visualisation. It is very standard to use PCA to visualise spatial variation from a large number of features, but this doesn't seem to be available. Can the authors add this?"

Response: We sincerely thank the reviewer for this constructive suggestion. We agree that Principal Component Analysis (PCA) is an essential tool for visualizing spatial variation and capturing the major axes of variance in high-dimensional multi-omics data.

In response to your suggestion, we have integrated PCA into the "Pixel-level Spatial Clustering" module of SMIntegration. Specific changes made in the manuscript:

Methods Section (Pixel-level Spatial Clustering): We have updated the description to include PCA as a core method for both visualization and parameter guidance. Revised text: "Five clustering methods are available (Figure 1C): Louvain (LV), LM, SLM [24], K-means after Principal Component Analysis (PCA), and K-means after Uniform Manifold Approximation and Projection（UMAP) [25,26]. To assist users in objective parameter selection, PCA is implemented as an initial visualization step to capture major spatial variation across many features. Users can leverage PCA variance plots to estimate the underlying complexity of the spatial domains and thus justify the selection of the number of clusters for subsequent analysis (Supplementary File 2). " (Page 7, Line 166).

Figure 1: We have updated the architecture diagram in Figure 1C to include PCA as part of the dimensionality reduction and clustering workflow.

Supplementary Material: We have updated Figure S5 to showcase the refined clustering interface which now integrates these assessment tools.

We believe this addition significantly improves the analytical depth and user guidance of our platform. Thank you again for this valuable recommendation.

Comment 6: "Spatial pattern detection is done via SpaGene. Many readers won't be familiar with this, so please add a few sentences describing how the algorithm works. Other methods (e.g. Moran's I correlation) would also benefit from more detailed description, perhaps in supplementary or on the website. "

Response: We sincerely thank the reviewer for this suggestion. We agree that providing more technical details on the underlying algorithms is essential for a broader audience to understand and utilize the platform effectively.

In response, we have expanded the description of SpaGene and Moran’s I in the Methods section. Specifically, we have clarified that SpaGene is a graph-based, model-free method that identifies spatially variable features by comparing observed high-expression subnetworks against random permutations using Earth Mover’s Distance (EMD). We have also added a brief explanation of how Moran’s I is used to quantify the spatial consistency between different omics layers.

Specific changes made in the manuscript: " This module applies SpaGene [22] to detect spatially variable (SV) features using a model-free, graph-based approach. Specifically, SpaGene constructs a k-nearest neighbor (k-NN) graph based on spatial coordinates and identifies high-expression subnetworks for each gene and metabolite. The spatial pattern strength is then quantified using Earth mover’s distance (EMDg) which measures the distance between the observed high-expression distribution on the spatial graph and a null distribution derived from random permutations. Features with significant EMDg values are identified as SV features. Subsequently, non-negative matrix factorization (NMF) partitions these features into distinct spatial modules based on their pattern similarity (Figure 1B). To quantify cross-omics associations, the platform employs Moran’s I [23], a measure of spatial autocorrelation, to evaluate the consistency of distribution patterns between identified gene and metabolite modules. Users can browse features within modules (Figure S4)." (Page 6, Line 152).

Comment 7: "Line 250: "1313 genes were highly expressed in the NA region, while 427 were highly expressed in the MO region (Figure 3D)" The figure shows different numbers: 1255 and 382."

Response: We sincerely apologize for the confusion caused by this error in our description. Upon re-examining our data, we realized that the numbers 1313 and 427 stated in the text were inadvertently calculated as the sum of both high-expression genes and high-intensity metabolites in the NA and MO regions, respectively. However, our intention was to specifically describe the number of high-expression genes in these two regions. The correct gene counts, as shown in Figure 3D, are 1255 and 382. We have now corrected the text to strictly refer to the high-expression gene counts. We appreciate the reviewer's meticulous review, which helped us ensure the consistency and accuracy of our report.

Specific changes made in the manuscript: "In terms of genes, 1255 genes were highly expressed in the NA region, while 382 were highly expressed in the MO region (Figure 3D)." (Page 11, Line 306)

Comment 8: "Line 241: "SMIntegration effectively addresses this challenge through its integrated cell type annotation and pixel registration" but you say that it doesn't perform coregistration of the two datasets? Clarify what you mean by 'registration'."

Response: We thank the reviewer for identifying this ambiguity. Our original use of the term "registration" in this context was intended to describe the process of matching and assigning labels between two already aligned modalities. We have made two major updates to address this:

1.Software Update: As noted in our response to Comment 1, we have now implemented a dedicated Spatial Registration module within the SMIntegration GUI (using the RNiftyReg package). This module allows users to perform the actual coregistration of the two datasets directly within the platform.

2.Clarified Terminology: In the revised manuscript, we have updated the description of the cell-type analysis workflow. We now use more precise language—"coordinate-based cell-type assignment" and "projecting labels"—to distinguish the transfer of biological annotations from the initial spatial alignment of the data slices.

Specific changes made in the manuscript: "SMIntegration effectively addresses this challenge through its integrated workflow of coordinate-based cell-type assignment and pixel registration. By projecting SingleR-identified labels from transcriptomics onto spatially aligned metabolomics pixels, the platform allows each pixel to be treated as a cell-type-specific sample (Figure 1D)." (Page 11, Line 293).

Comment 9: "When testing the app using the demo data, in step 4 "differential analysis" I was not able to input the cluster numbers or define the regions interactively as the app would not accept inputs. The cluster numbers just disappeared when clicking elsewhere on the app. For the interactive definition, the box "select feature to plot" was permanently blank. I could not perform the differential analysis."

Response:

We sincerely thank the reviewer for taking the time to test our application and for reporting this issue. We apologize for the inconvenience and confusion you experienced during the differential analysis step.

We have carefully reproduced the entire workflow and verified that all functions perform as expected under standard operating conditions. Having examined the issue you described, we suspect that the inability to select regions or input cluster numbers may have been caused by skipping the "Cell Annotation" step after completing the "Clustering Analysis". The Differential Analysis module depends on the data generated during the Cell Annotation step (even if just using the demo or default annotation). Without this step, the downstream data structures required for ROI selection are not initialized.

To clarify the correct workflow, we have included a GIF below demonstrating the usage of the ROI selection module. You can also refer to the MP4 file in the attachments for supplementary information.

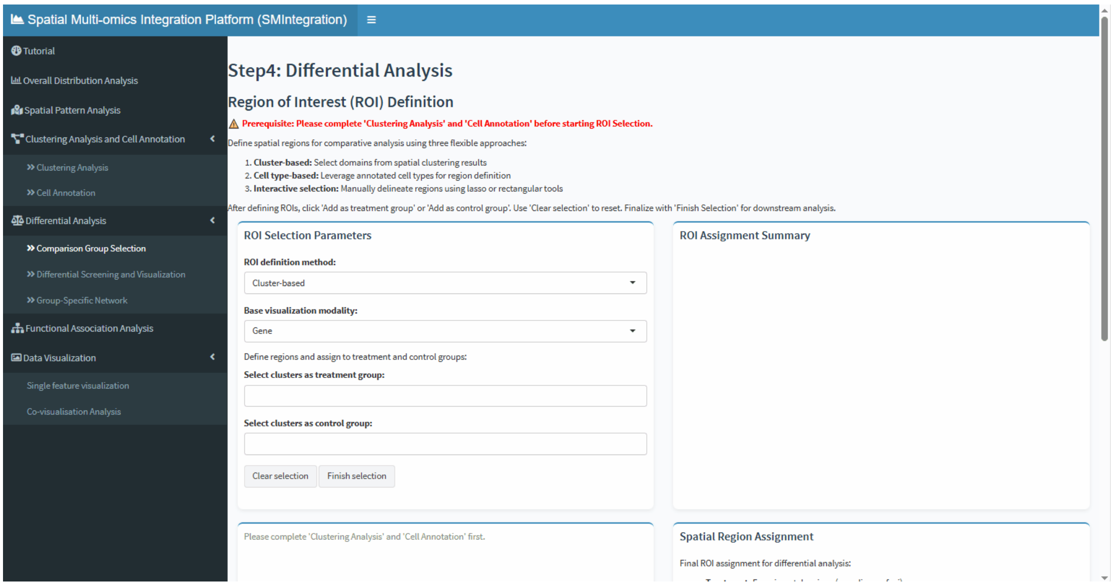

Furthermore, to enhance the user experience and prevent similar issues, we have implemented improved guidance and error-prevention mechanisms in the ROI selection module:

Explicit Step Numbering: The key prerequisite steps in the interface are now clearly labeled as Step 3.1 (Cluster Preprocessing), Step 3.2 (Cluster Analysis), and Step 3.3 (Cell Annotation) to visually outline the required workflow sequence.

Intelligent Input Validation: Within the ROI selection module, the system now performs real-time checks for the required data structures. If the necessary data is detected as missing or uninitialized, the interface dynamically displays a prompt: " Please complete 'Clustering Analysis' and 'Cell Annotation' before starting ROI Selection." This mechanism ensures users successfully finish all necessary upstream steps before attempting differential analysis.

These optimizations will ensure all users clearly understand the dependencies between modules and receive explicit guidance if an operation is blocked.

Supplementary Material: We have updated Figure S5 and S6 to showcase the changes in the prompts of the clustering analysis and cell annotation module.

Comment 10: "In my review of the github I found that the code is not commented at all. I was unable to find any R or Python script which contained comments. This is extremely poor practice in software development and needs to be rectified before publication. Other aspects such as unit tests and validation also did not appear to be present."

Response: We sincerely apologize for the lack of adequate documentation and formal validation in the initial version of our repository. We fully agree that for scientific software, transparency of code and verification of analytical logic are essential for reproducibility.

While SMIntegration is developed as a standalone Shiny application rather than a traditional R package, we recognize that the same standards of software rigor must apply. We have performed a comprehensive overhaul of the GitHub repository to meet these standards:

1. Systematic Code Commenting: We have reviewed and commented all R scripts (including UI, Server logic, and utility functions) and Python scripts. Every core function now includes detailed header comments explaining the input requirements, mathematical logic, and output formats.

2. Implementation of a Dual-Layer Validation Suite: To address the requirement for "unit tests and validation," we implemented a two-tiered validation framework:

2.1 Modular Unit Tests for the Analytical Pipeline (/validation_pipeline): Core modules (Preprocessing, Clustering, Differential Expression, Functional Association) were refactored into standalone, testable units. Using the downsampled demo dataset, we conducted targeted tests for each:

a. Preprocessing & Clustering: Validates data normalization, multi-omics integration, and Louvain clustering stability (01_test_preprocessing.R, 02_test_clustering.R).

b. Biologically-Contextualized Validation: A dedicated test (03_test_differential_analysis.R) automatically maps cell annotations and replicates the key Experimental (ACNT1/2) vs. Control (MOL1/2) group comparison from the manuscript.

c. Pathway Analysis: Confirms accurate mapping of differential features to KEGG pathways (04_test_functional_association.R).

2.2 Figure Reproduction for Output Verification (run_validation_figure.R): A regression test ensures reproducibility of final visual results. This script autonomously regenerates key manuscript figures (Figures 1-4) from archived intermediate data (RDS files), verifying visualization pipeline reliability.

For user convenience, master execution scripts (run_pipeline_validation.R and run_validation_figure.R) are provided in the root directory, enabling a single-command execution of the entire validation suite to verify both computational integrity and graphical output fidelity.

Specific changes made in the manuscript:

Software Implementation Section: Added a description of the validation protocols and code documentation efforts. Revised text: "SMIntegration is a web-based GUI implemented in R (v4.4.2) using Shinyproxy and Docker. To ensure the reliability of the integrated analytical workflow, we have implemented a systematic validation protocol. This includes (1) comprehensive inline documentation for all core analytical scripts on GitHub; and (2) a standalone, dual-layer validation suite comprising modular unit tests for core computational modules (e.g., spatial normalization, clustering, differential expression) and regression tests for figure reproducibility. "(Page 5, Line 112)

We believe these improvements significantly enhance the transparency and reliability of the SMIntegration platform.

References

[16] Chen A, Liao S, Cheng M, Ma K, Wu L, Lai Y, et al. Spatiotemporal transcriptomic atlas of mouse organogenesis using DNA nanoball-patterned arrays. Cell 2022; 185(10): 1777–1792.e21.

[17] Clayden J, Modat M, Presles B, Anthopoulos T, Daga P. RNiftyReg: Image registration using the "NiftyReg" library. R package version 2.8.4, 2024.

[18] Modat M, Cash DM, Daga P, Winston GP, Duncan JS, Ourselin S. Global image registration using a symmetric block-matching approach. J Med Imaging (Bellingham) 2014; 1: 024003.

[19] Modat M, Ridgway GR, Taylor ZA, Lehmann M, Barnes J, Hawkes DJ et al. Fast free-form deformation using graphics processing units. Comput Methods Programs Biomed 2010; 98: 278–284.

[20] Clayden JD, Maniega SM, Storkey AJ, King MD, Bastin ME, Clark CA. TractoR: Magnetic Resonance Imaging and Tractography with R. J. Stat. Soft. 2011; 44: 1–18.

Response to Reviewer Comments

1. Summary

We sincerely appreciate the reviewer's thoughtful questions regarding the algorithmic transparency and technical implementation of SMIntegration. These inquiries have prompted us to significantly clarify the platform's internal logic, particularly concerning how biological associations are computationally derived and how multi-modal data with differing resolutions are integrated. In response to your comments, we have revised the manuscript to provide a more rigorous and detailed explanation of our methodologies. The key revisions are summarized below:

1) Clarified Discovery Logic for Cell-Specific Associations: We have expanded the "Results" and "Methods" sections to systematically explain the three-step computational process underlying our cell-type analysis: (1) Coordinate Transfer, where transcriptomics-derived labels are projected onto registered metabolomics pixels; (2) Parallel Differential Testing, which independently identifies markers for each omics layer; and (3) Functional Convergence, where the algorithm automatically flags pathway co-annotation (e.g., Slc6a11 and GABA in astrocytes). This ensures the "discovery" process is presented as a reproducible, algorithmic output rather than an opaque result.

2) Detailed "Align-Register-Fuse" Integration Framework: We have substantially elaborated on the technical algorithms used to handle resolution discrepancies. The revised manuscript now explicitly describes our high-to-low aggregation (binning) strategy for resolution matching, the implementation of the RNiftyReg algorithm for robust spatial registration, and the pixel-level matrix concatenation method used for joint clustering.

3) Enhanced Documentation: To support these clarifications, we have added five new technical references acknowledging the registration libraries and updated the Methods section to serve as a comprehensive technical guide for the integration workflow.

We believe these revisions provide the necessary technical depth to validate the platform's robustness. Detailed responses to each specific comment are provided below.

2. Point-by-point response to Comments and Suggestions for Authors

Comment 1: " The authors should explain more detailed for cell type analysis of their algorithm. How to figure out the association between astrocyte enriched GABA metabolism and Slc6a11?"

Response: We thank the reviewer for this constructive comment. To clarify how SMIntegration "figures out" such associations from high-dimensional data, we have

expanded the description in the Results section (Subheading: "Cell-Specific Metabolite Analysis").

The discovery of the association between Slc6a11 and GABA was achieved through a systematic three-layer filtering algorithm rather than manual selection:

Firstly, Spatial Coordinate Transfer. The platform first registers the Stereo-seq and AFADESI-MS data. Cell-type labels from the transcriptomics layer are then projected onto the metabolomics pixels based on their shared spatial coordinates. This defines the "non-telencephalon astrocyte (NA)" region at the pixel level.

Secondly, Parallel Differential Testing. The software executes independent Wilcoxon rank-sum tests for both genes and metabolites across these defined regions. Slc6a11 and GABA were independently identified as significant "markers" for the NA region.

Thirdly, Functional Convergence Screening. The "Functional Association" module takes the list of differentially expressed genes (DEGs) and differentially abundant metabolites (DAMs) and performs a co-enrichment analysis. The algorithm automatically identifies pathways where both a DEG and a DAM are present. In this case, the system flagged the GABAergic synapse pathway, thereby establishing the functional link between the transporter gene (Slc6a11) and the metabolite (GABA).

We have revised the manuscript to emphasize this automated discovery logic, ensuring that the process of "locating" these associations is transparent and reproducible.

Revised Text in Manuscript:

1. In "Methods - Cell Type Annotation": "Cell types are annotated for transcriptomics using SingleR [27] based on reference datasets (MouseRNAseqData, HumanPrimaryCellAtlasData). Since the transcriptomics and metabolomics modalities are spatially registered, SMIntegration transfers these cell-type labels to the corresponding metabolomics pixels based on their overlapping spatial coordinates. This allows the definition of cell-type-specific regions of interest (ROIs) for subsequent cross-modal differential analysis." (Page 7, Line 187)

2. In "Results - Cell-Specific Metabolite Analysis":" Deciphering gene expression and metabolite abundance changes within specific cell types is crucial for a deeper understanding of cellular function. However, spatial metabolomics data itself lacks direct cell type annotation capabilities. SMIntegration effectively addresses this challenge through its integrated workflow of coordinate-based cell-type assignment and pixel registration. By projecting SingleR-identified labels from transcriptomics onto spatially aligned metabolomics pixels, the platform allows each pixel to be treated as a cell-type-specific sample (Figure 1D). We demonstrate this by comparing two functionally distinct glial cell populations in the mouse brain: regions dominated by non-telencephalon astrocytes (NA) versus regions dominated by Mature

oligodendrocytes (MO) (Figure 3A, Figure S7C). UMAP analysis showed clear differences in metabolite (Figure 3B) and gene (Figure 3C) expression between these two cell types.

To systematically pinpoint biological associations from the high-dimensional data, we applied a parallel discovery logic. Through differential screening, SMIntegration identified 103 differentials mass features, with 58 upregulated in NA and 45 upregulated in MO. In terms of genes, 1255 genes were highly expressed in the NA region, while 382 were highly expressed in the MO region (Figure 3D). To determine the functional synergy between these two lists, the platform's "Functional Association" module was utilized to perform automated co-annotation analysis. Notably, the gene Slc6a11 and the metabolite gamma-Aminobutyric acid (GABA) were algorithmically flagged as they both converged on the GABAergic synapse pathway (Figure S15A). Both were upregulated in the NA region (Figure S15B, C). Slc6a11 encodes a sodium-dependent transporter [36], and its absence can lead to GABA accumulation and an imbalance in neuronal excitability, affecting cognitive function [37]. These differential results are consistent with the cellular functions of astrocytes, which play a role in GABA synthesis and transmission." (Page 11, Line 291)

Comment 2: " Due to resolution difference, which algorithm has been utilized or developed for integration of spatial transcriptomics and spatial metabolomics in the web-tool?"

Response: We thank the reviewer for this important technical question. SMIntegration addresses the challenge of integrating multi-modal data with differing resolutions through a systematic "Align-Register-Fuse" computational framework:

Resolution Alignment (Aggregation/Binning): To harmonize the resolution difference (e.g., 500 nm ST vs. 50 μm SM), the platform utilizes a high-to-low aggregation algorithm. ST data are binned to match the specific grid resolution of the SM imaging pixels, ensuring that both datasets share a consistent spatial unit.

Spatial Registration (RNiftyReg): As now detailed in the revised manuscript, we have integrated a dedicated registration module implemented via the RNiftyReg package. This algorithm supports both linear and non-linear transformations, allowing for precise alignment of tissue sections even when subtle morphological distortions exist between adjacent slices.

Multimodal Integration Algorithm (Pixel-level Fusion): Once aligned, the "integration" itself is achieved through pixel-level matrix concatenation. The platform treats each registered pixel as a unified observation, combining its transcriptomic and metabolomic profiles into a high-dimensional feature vector. This fused matrix then serves as the input for joint clustering algorithms, which identify shared spatial domains by considering both modalities simultaneously.

We have updated the Methods section to provide a more rigorous description of these algorithms and their implementation.

Revised Text in Manuscript:

1. On Resolution Alignment and Registration Algorithm: " SMIntegration input requires processed spatial metabolomics and transcriptomics data matrix. Since resolutions differ, higher-resolution data should be aggregated to match the lower (e.g., binning 500 nm transcriptomics by 100 to 50 µm metabolomics) [16]. To facilitate seamless integration, SMIntegration features a dedicated spatial registration module implemented using the RNiftyReg package [17], which provides an R interface to the NiftyReg library [18,19] and is part of the TractoR framework [20]. This module supports both linear (block-matching) and non-linear (free-form deformation) transformations to align the two modalities. Detailed tutorials for this registration module are provided in Supplementary File 1. Users also have the option to perform coregistration externally using the Python-based SpatialData ecosystem [21]; the aligned data can then be imported into SMIntegration in a compatible format for subsequent integrated analysis. Two input formats are supported: (1) text matrices containing feature name, spatial x/y coordinates, and values (Figure S2A); (2) Seurat objects with coordinates and abundance in designated slots (Figure S2B). Upload requirements are detailed on the help page." (Page 6, Line 131)

References: We have added four five new references [16-20] to acknowledge the developers of the registration library and the TractoR framework.

2. On Multimodal Integration (Fusion) Algorithm, section: Pixel-level Spatial Clustering: " Integrated data combines both modalities by pixel coordinates. This integration algorithm operates by concatenating the pre-processed, scaled feature matrices from both transcriptomics and metabolomics into a unified multimodal matrix. By treating each spatially registered pixel as a shared observation containing both gene and mass feature dimensions, the platform enables joint dimensionality reduction and clustering to uncover synchronized spatial domains. A Sankey diagram compares clustering concordance (Figure S5)." (Page 7, Line 179)

References

[16] Chen A, Liao S, Cheng M, Ma K, Wu L, Lai Y, et al. Spatiotemporal transcriptomic atlas of mouse organogenesis using DNA nanoball-patterned arrays. Cell 2022; 185(10): 1777–1792.e21.

[17] Clayden J, Modat M, Presles B, Anthopoulos T, Daga P. RNiftyReg: Image registration using the “NiftyReg” library. R package version 2.8.4, 2024.

[18] Modat M, Cash DM, Daga P, Winston GP, Duncan JS, Ourselin S. Global image registration using a symmetric block-matching approach. J Med Imaging (Bellingham) 2014; 1: 024003.

[19] Modat M, Ridgway GR, Taylor ZA, Lehmann M, Barnes J, Hawkes DJ et al. Fast free-form deformation using graphics processing units. Comput Methods Programs Biomed 2010; 98: 278–284.

[20] Clayden JD, Maniega SM, Storkey AJ, King MD, Bastin ME, Clark CA. TractoR: Magnetic Resonance Imaging and Tractography with R. J. Stat. Soft. 2011; 44: 1–18.
