# Supplementary material for: SMIntegration: A web tool for comprehensive spatial metabolomics and transcriptomics integrated analysis and visualization
Source: Gigascience. 2026 Mar 24;15:giag033. doi: 10.1093/gigascience/giag033 (PMC13159472; doi:10.1093/gigascience/giag033)
Supplement: giag033_Reviewer_1_Report_original_submission — Reviewer 1 -- 11/21/2025 [file giag033_reviewer_1_report_original_submission.pdf]

Reviewer Report

Title: SMIntegration: A Web Tool for Comprehensive Spatial Metabolomics and Transcriptomics Integrated Analysis and Visualization

Version: Original Submission **Date:** 11/21/2025

Reviewer name: Reviewer 1

Reviewer Comments to Author:

This paper presents a new web based tool for integration of spatial transcriptomic and metabolomic data. These technologies are becoming more widely used and there is a need for bioinformatic approaches that can analyse them together. The tool is well conceptualised and fairly clearly described. When I tested on the authors' own example data, I was able to run most but not all the functionality of the tool. I have a few comments which I hope would improve the presentation of the tool before publication.Specific comments1. First, the software requires metabolomics and transcriptomics data which has already been coregistered and transformed to the same spatial resolution. These two tasks - especially coregistration - are challenging and a barrier to researchers intending to use the presented package. The authors point to a script on their github to coregister data, but this has zero documentation so is not a viable option. Please give better instructions and point to other options for these tasks.2. The methods employed require various parameters to be set. For example, the number of clusters or clustering resolution. How should users set these? It would be nice to provide some (semi)automatic methods for picking the best values (or at least objective assessment of the results, allowing different values to be compared). Defaults should be justified / explained. E.g. why is the default log2FC threshold 2.6?3. Some of the language, to my mind, over interprets the data. For example l169 "understanding of group specific regulatory mechanisms" I don't think you can infer mechanism directly from this type of exploratory analysis. Line 234 "discovery provides a novel molecular framework" - this is a potential hypothesis that can be tested, I don't think you can say it is a 'discovery'. Line 270 "strongly suggests the presence of a finely tuned inhibitory regulation" - this is just a correlation, and not 'strong' evidence in my opinion.4. Functional enrichment. For metabolomics this is not straightforward. Especially there will be uncertainty in the metabolite identities, as well as sparse mapping of metabolites to pathways. How are these aspects taken into account? At least the metabolite identification uncertainty should be acknowledge with a warning message. Is there a minimum number of metabolites/transcripts required to include a pathway? Mapping only a single metabolite to a pathway invalidates pathway testing. For enrichment (Fisher test), what background set is used? Use of the full set of e.g. KEGG compounds will greatly inflate p-values. What version of KEGG is used? What type of multiple testing is applied on for the pathway tests? Finally in the pathway enrichment plots, p-values are represented on a color scale 0-1 so it is hard to see any significance threshold. E.g. in figure 4D are any pathways significant? It would be clearer to visualise the log10 p-value here, with a specific mark for the cut-off. It also looks like the metabolites show higher rich factors than the transcripts. Rich factor is not defined, but if it relates to coverage of the pathway then this doesn't make sense - one would expect transcripts to have a higher coverage than metabolites.5. Spatial pattern visualisation. It is

very standard to use PCA to visualise spatial variation from a large number of features, but this doesn't seem to be available. Can the authors add this?6. Spatial pattern detection is done via SpaGene. Many readers won't be familiar with this, so please add a few sentences describing how the algorithm works. Other methods (e.g. Moran's I correlation) would also benefit from more detailed description, perhaps in supplementary or on the website.7. Line 250: "1313 genes were highly expressed in the NA region, while 427 were highly expressed in the MO region (Figure 3D)" The figure shows different numbers: 1255 and 382.8. Line 241: "SMIntegration effectively addresses this challenge through its integrated cell type annotation and pixel registration" but you say that it doesn't perform coregistration of the two datasets? Clarify what you mean by 'registration'.9. When testing the app using the demo data, in step 4 "differential analysis" I was not able to input the cluster numbers or define the regions interactively as the app would not accept inputs. The cluster numbers just disappeared when clicking elsewhere on the app. For the interactive definition, the box "select feature to plot" was permanently blank. I could not perform the differential analysis.10. In my review of the github I found that the code is not commented at all. I was unable to find any R or Python script which contained comments. This is extremely poor practice in software development and needs to be rectified before publication. Other aspects such as unit tests and validation also did not appear to be present.

Level of Interest

Please indicate how interesting you found the manuscript: Choose an item.

Quality of Written English

Please indicate the quality of language in the manuscript: Choose an item.

Declaration of Competing Interests

Please complete a declaration of competing interests, considering the following questions:

- Have you in the past five years received reimbursements, fees, funding, or salary from an organisation that may in any way gain or lose financially from the publication of this manuscript, either now or in the future?
- Do you hold any stocks or shares in an organisation that may in any way gain or lose financially from the publication of this manuscript, either now or in the future?
- Do you hold or are you currently applying for any patents relating to the content of the manuscript?
- Have you received reimbursements, fees, funding, or salary from an organization that holds or has applied for patents relating to the content of the manuscript?
- Do you have any other financial competing interests?
- Do you have any non-financial competing interests in relation to this paper?

If you can answer no to all of the above, write 'I declare that I have no competing interests' below. If your reply is yes to any, please give details below.

I declare that I have no competing interests

I agree to the open peer review policy of the journal. I understand that my name will be included on my report to the authors and, if the manuscript is accepted for publication, my named report including any attachments I upload will be posted on the website along with the authors' responses. I agree for my report to be made available under an Open Access Creative Commons CC-BY license (http://creativecommons.org/licenses/by/4.0/). I understand that any comments which I do not wish to be included in my named report can be included as confidential comments to the editors, which will not be published.

Choose an item.

To further support our reviewers, we have joined with Publons, where you can gain additional credit to further highlight your hard work (see: https://publons.com/journal/530/gigascience). On publication of this paper, your review will be automatically added to Publons, you can then choose whether or not to claim your Publons credit. I understand this statement.

Yes Choose an item.
