# Supplementary material for: SMIntegration: A web tool for comprehensive spatial metabolomics and transcriptomics integrated analysis and visualization
Source: Gigascience. 2026 Mar 24;15:giag033. doi: 10.1093/gigascience/giag033 (PMC13159472; doi:10.1093/gigascience/giag033)
Supplement: giag033_Reviewer_3_Report_original_submission — Reviewer 3 -- 12/15/2025 [file giag033_reviewer_3_report_original_submission.pdf]

Reviewer Report

Title: SMIntegration: A Web Tool for Comprehensive Spatial Metabolomics and Transcriptomics Integrated Analysis and Visualization

Version: Original Submission **Date:** 12/15/2025

Reviewer name: Reviewer 3

Reviewer Comments to Author:

This manuscript presents a web application for the integrated analysis of spatial metabolomics and spatial transcriptomics data, which will be of great interest to many researchers. However, the tool currently lacks several important data preprocessing steps. Users must perform these steps using other software, which demands substantial programming skills and compromises user-friendliness. My specific comments are as follows:Major:1. Data preprocessing for spatial metabolomics data was done using the Cardinal package, and for spatial transcriptomics data using Seurat in this manuscript. This significantly reduces user-friendliness and requires substantial programming skills to use SMIntegration, making this tool difficult for many users. Please consider integrating the data preprocessing steps directly into SMIntegration.2. Please consider adding a registration function in SMIntegration using the SpatialData package, as this is one of the most critical steps for integrated spatial metabolomics and transcriptomics data analysis.3. Line 143: LogNormalization does not work well for spatial metabolomics data due to its highly sparse nature. More common methods are TIC normalization and root mean squared normalization.Minor:1. Line 90: Add the word "spatial" (e.g., "spatial metabolomics").2. Line 100: Your workflow does not include the complete processing steps. Please consider removing the word "preprocessing."3. Line 159: Why is the default threshold for the fold-change value so small?4. Line 189: The authors appear to confuse "mass features/peaks" with "metabolites." Please replace "metabolites" with "mass peaks" or "mass features" throughout the manuscript and in Figure 1.5. Line 188: SManalyst identifies metabolites only through monoisotopic ion matching, which is considered Level 3 metabolite identification. The authors should state this clearly in the manuscript and exercise caution when interpreting the data throughout the manuscript.

Level of Interest

Please indicate how interesting you found the manuscript: Choose an item.

Quality of Written English

Please indicate the quality of language in the manuscript: Choose an item.

Declaration of Competing Interests

Please complete a declaration of competing interests, considering the following questions:

- Have you in the past five years received reimbursements, fees, funding, or salary from an organisation that may in any way gain or lose financially from the publication of this manuscript, either now or in the future?
- Do you hold any stocks or shares in an organisation that may in any way gain or lose financially from the publication of this manuscript, either now or in the future?
- Do you hold or are you currently applying for any patents relating to the content of the manuscript?
- Have you received reimbursements, fees, funding, or salary from an organization that holds or has applied for patents relating to the content of the manuscript?
- Do you have any other financial competing interests?
- Do you have any non-financial competing interests in relation to this paper?

If you can answer no to all of the above, write 'I declare that I have no competing interests' below. If your reply is yes to any, please give details below.

I declare that I have no competing interests

I agree to the open peer review policy of the journal. I understand that my name will be included on my report to the authors and, if the manuscript is accepted for publication, my named report including any attachments I upload will be posted on the website along with the authors' responses. I agree for my report to be made available under an Open Access Creative Commons CC-BY license (http://creativecommons.org/licenses/by/4.0/). I understand that any comments which I do not wish to be included in my named report can be included as confidential comments to the editors, which will not be published.

Choose an item.

To further support our reviewers, we have joined with Publons, where you can gain additional credit to further highlight your hard work (see: https://publons.com/journal/530/gigascience). On publication of this paper, your review will be automatically added to Publons, you can then choose whether or not to claim your Publons credit. I understand this statement.

Yes Choose an item.
